# Conducting household surveys on reproductive health in urban settings: lessons from Karachi, Pakistan

**DOI:** 10.1186/s12874-021-01216-x

**Published:** 2021-02-18

**Authors:** Mir Baz Khan, Sidrah Nausheen, Imtiaz Hussain, Kristy Hackett, Kaneez Zehra, Khalid Feroze, David Canning, Iqbal Shah, Sajid Bashir Soofi

**Affiliations:** 1 Department of Pediatrics & Child Health, Center of Excellence in Women & Child Health, Aga Khan University, Stadium Road, PO Box 3500, Karachi, 74800 Pakistan; 2 Department of Obstetrics & Gynecology, Aga Khan University, Karachi, Pakistan; 3 Department of Global Health, Harvard T.H. Chan School of Public Health, Boston, USA

**Keywords:** Household surveys, Challenges, Loss-to-follow up, Implementation, Sampling frame

## Abstract

**Background:**

Data collection is the most critical stage in any population health study and correctly implementing fieldwork enhances the quality of collected information. However, even the most carefully planned large-scale household surveys can encounter many context-specific issues. This paper reflected on our research team’s recent experience conducting surveys for a quasi-experimental evaluation of a reproductive health program in urban areas of Karachi, Pakistan. We aim to describe the issues encountered and lessons learned from this process, and present some potential solutions for conducting future household surveys in similar urban environments.

**Methods:**

The study followed a three-stage random sampling design. Initially, a Geographical Information System (GIS) was used to construct the sampling frame with union council (UC) area mapping and cluster demarcation followed by random selection of clusters in the selected UCs within the intervention and control sites. The second stage involved a complete household listing in selected clusters and the final stage was a random sampling of households with eligible women.

**Result:**

This paper describes the issues that were encountered including technical problems related to GIS demarcation of cluster boundaries and hand-held devices for computer assisted personal interviews (CAPI), household listing, interviewing respondents on sensitive topics and their expectations, and ensuring privacy during the survey.

**Conclusion:**

This study identifies a number of unique barriers to conducting household surveys in Karachi and highlights some key lessons for survey research in urban settlements. GIS mapping technology is a cost-effective method for developing sampling frames in resource-constrained settings. Secondly, the strategy of interviewing women immediately after the cluster is listed may be applied to make it easier to re-locate selected respondents and to reduce loss-to-follow up. Understanding local norms and developing culturally appropriate strategies to build trust with communities may significantly improve survey participation. Researchers should hire experienced female enumerators and provide continuous training on best practices for interviewing women on sensitive reproductive health topics in urban communities.

**Supplementary Information:**

The online version contains supplementary material available at 10.1186/s12874-021-01216-x.

## Background

The United Nations (UN) has recognized universal access to reproductive healthcare as a priority for global health, and one of the Sustainable Development Goals (SDGs) is to ensure universal access to sexual and reproductive health (SRH) services, including family planning, with a particular focus on the use of modern contraceptive methods (indicator 3.7.1) [1]. Women’s reproductive health services in low- and middle-income countries (LMICs) have not fully met global human rights standards [2]. Family planning programs in several LMICs are fragile due to constrained existing policies and dependence on donor funding. The unmet need for contraception is the highest among the poorest couples [3]. Improving access to family planning in such settings can help women and couples meet their reproductive goals and improve maternal and child health [3].

For decades, family planning has been a contentious issue in Pakistan, which has the highest total fertility rate (3.8 children per woman) in South Asia after Afghanistan. While knowledge of family planning is nearly universal (98–99%), the overall contraceptive prevalence rate (CPR) is only 34% in Pakistan [4, 5]. The major obstacles to contraceptive use are lack of awareness of specific modern methods, perception of husband’s preferences and attitudes related to family planning, health concerns associated with contraceptive use, and perceived access to family planning services [6]. Moreover, in Pakistani society, having open discussions about sexual preferences is taboo.

Several issues impede reliable data collection among marginalized groups [7, 8] including the sensitive nature of SRH topics and questions, and in some cases, women’s negative experiences related to intimate or non-intimate physical or sexual violence [9]. Strict religious identity affects some women’s willingness to discuss contraception with others. Likewise, strong institutionalized religious doctrines in combination with cultural beliefs in urban communities, and men’s role as the overall head of household makes women less likely to share their experiences and practices related to family planning [10]. Ensuring quality family planning data therefore remains a challenge in many LMICs [11].

In any research process, data collection is the first stage of gathering information with respect to respondents’ needs, goals, and attitudes toward the problem area. In the process of obtaining information from respondents, researchers may encounter unanticipated challenges. Enumerators working in LMICs often encounter respondents who are reluctant to participate in the interview, which makes it difficult to build rapport [12]. A number of factors may influence a respondent’s willingness to participate. For example, the response of the interviewee depends on the type of clothes worn (formal or casual) during the field visit. Enumerators may also face issues persuading a respondent in a joint family system to participate, and convincing some respondents to discuss sensitive topics such as sexuality [13]. The respondent’s literacy and understanding of the questions consequently affect the process of data collection. If respondents perceive a risk of breaches in confidentiality, they may be reluctant to reveal personal information to the enumerator during the interview [14, 15]. Similarly, social and cultural taboos in some religious and cultural groups prevent women from discussing sexual and reproductive health issues with someone other than close family and friends. A study of women from constituencies of highly religious groups in urban Zimbabwe reported women’s refusal to participate in interviews related to SRH because of their religious beliefs. In the context of Pakistan, strong societal taboos linked with moral values, gender roles, and religious opposition, restrict women from openly discussing SRH services in religious communities [16, 17]. There are also a cultural tendency to avoid sharing personal information with untrusted outsiders [18, 19]. Studies from other LMICs have shown that lack of trust is a challenge particularly in settings where previous research provided no direct benefit or feedback to respondents [20, 21]. Moreover, respondents’ busy schedules and lengthy questionnaires may lead to refusal or incomplete surveys. Understanding local norms and developing culturally appropriate strategies to build trust within communities significantly reduces refusals [20, 21]. Additionally, researchers in South Africa reported that urban settlements do not have adequate space for interviews, therefore ensuring privacy was challenging . Furthermore, densely populated urban settings pose logistical as well as technical difficulties for researchers such as identifying eligible respondents, poor transportation and infrastructure, and lack of political and administrative willingness to support independent researchers [19].

### Evaluation of the willows reproductive health program

The present study was part of an evaluation of the Willows Reproductive Health Program [22], a large community-based reproductive health program providing reproductive health information and education to women of reproductive age in Karachi, Pakistan. The program included both prospective and retrospective assessments of separate intervention and control sites. These sites included communities with adequate provision of family planning services and typically house women of low socio-economic status. The initial stage of the program involved a registration period in which program field workers enrolled all eligible women living in the defined intervention area. The field educators then carried out home visits informing women about the benefits of family planning, provided information on the range of available contraceptive methods, and referred women to locally-based healthcare services, when required, and coordinated with providers to ensure the delivery of high-quality family planning services. The program also applied an algorithm that determined women most in need of family planning counseling, and field educators prioritized these women to receive education, information, and referrals. The non-users with high risk of unintended pregnancy, ineffective users of traditional methods, and unsatisfied users of modern methods were given highest priority for home visits. The retrospective assessment was carried out between 2013 and 2015 and prospective assessment from 2017 to 2020 in urban areas of Karachi, Pakistan. This paper reflects our research team’s recent experiences carrying out these assessments for a quasi-experimental evaluation of the Willows reproductive health program. We aim to describe the issues encountered and lessons learned from this process, and recommend some potential solutions for future SRH surveys in similar urban dwellings.

## Methodology

### Parent study design

In this paper, we describe lessons learned during the undertaking of a larger impact evaluation, which consisted of two components. Component 1 was a baseline prospective assessment conducted prior to implementation of a reproductive health program, and component 2 was a cross-sectional retrospective survey in a site where the program has ended.

### Study sites and population

The evaluation included both prospective and retrospective assessments of four separate intervention and control sites in urban settlements of Karachi, Pakistan: Jamshed Town, Yousuf Goth, Korangi Town, and PIB colony and Dalmia/Shanti communities. The program was implemented from April 2013 through September 2015 in Korangi Town (for retrospective assessment), and from June 2017 to March 2020 in Jamshed Town (for prospective assessment). We selected control sites to match the intervention areas in terms of socio-demographic characteristics, such as type of area (urban or peri-urban), population size, ethnic composition, and dominant language. Women were eligible if they were aged 16–44 years for prospective assessment and 16–49 years for retrospective assessment, married, usual members of the household, and spoke at least one of the four most common languages (Urdu, Pashto, Sindhi, or English).

Jamshed town is the most populated (0.73 million) municipality in the East district of Karachi with a majority Muslim population. The town is populated by diverse ethnic groups including Muhajirs, Punjabis, Sindhis, Kashmiris, Seraikis, Pashtuns, Balochis, Memons, Bohras, and Ismailis, and is also home to minority groups such as Christians, Parsis, and Hindus. Yousuf Goth, with nearly 1 million inhabitants, is a peri-urban setting in the neighborhood of Malir district with a majority Muslim population of various ethnic groups. Jamshed town and Yousuf Goth share similar socio-demographic characteristics and consist of large slum areas as well as some upper-middle income neighborhoods. Both areas have dynamic working-class populations and have public/private schools, malls and shopping plazas. Annual religious festivals and variability in weather patterns influence seasonal migration within and between districts and provinces. For example, families of slum areas often move to interior Sindh districts to harvest crops from April to July each year. Some families live temporarily in rented houses in selected settings and frequently shift between nearby communities.

On the other hand, Korangi town with a population of 2.4 million is a peri-urban neighborhood in East Karachi with a multi-ethnic population including Muhajirs, Sindhi, Balochi, Pashtuns, and Gilgiti. This is an industrial area and is home to low and middle-income families from Afghanistan and Bangladesh who migrated for the purpose of employment in the garment and leather factories. PIB and Dalmia/Shanti Nagar have majority Muslim populations of 1 million and 0.1 million, respectively, and are peri-urban areas located in Gulshan town. In both areas, school enrollment is low and many children work as helpers in factories. Some women work in garment factories, while others work as housekeepers, and many have low-paying private school teaching jobs.

### Sampling strategy and sample size

Both prospective and retrospective surveys followed a three-stage random sampling design. Initially, Geographical Information System (GIS) technology was used to construct the sampling frame with distinct area mapping and cluster demarcation of the intervention and control sites. GIS technology was used because Pakistan lacks reliable and updated statistics related to structure and household numbers in union councils of urban slums. Secondly, it is a more cost-effective method than in-person visits to validate cluster boundaries, and minimizes time spent searching for reliable statistics and developing sampling frames in person. Based on the geographical demarcation by GIS, 283 clusters were formed in the intervention site and 200 clusters in the control site for the prospective assessment, and 548 clusters in the intervention site and 160 in the control sites for the retrospective assessment. Each cluster was created on a cadastral scale consisting of 60–100 structures. The second stage involved random selection of clusters in the intervention and comparison sites. A total of 105 clusters from the intervention and 100 from control site for the prospective assessment, and 110 clusters in each site for the retrospective assessment were randomly selected for inclusion in the study.

The second stage involved a complete household listing in selected clusters and random sampling of eligible women. The data management unit of Aga Khan University (AKU) developed an android application program for the household listing activity. All households were included in the listing, and the questionnaire sought to determine eligible women who were between the ages of 16–44 years for the prospective component and 16–49 for the retrospective component. Field teams also collected pertinent details on the household location, including Global Positioning System (GPS) coordinates, addresses, written directions, and the name of the household head. The application was tested multiple times to fix bugs and queries prior to the household listing. The household listing enrolled 8179 households in the intervention and 6406 households in the control sites for the prospective assessment, and 9010 in the intervention and 8182 in control sites for the retrospective assessment to generate a sampling frame for selection of households (secondary sampling units).

The final stage involved random selection of women from each cluster. A sample size of ~ 2000 women from each site was required to achieve sufficient statistical power. The study team used the household listing to identify 2019 eligible women in the intervention area and 2147 eligible women in the control sites for prospective survey; and 2750 eligible women each from the intervention and control sites for the retrospective assessments using a computerized process. The calculated sample size was powered to detect a 5% (percentage point) difference in critical value of CPR in intervention sites compared to control sites at 0.05 significance with 90% probability of exceeding the critical value on a two-sided test. This process was carried out in STATA Software for Statistics and Data Science using a uniform [0, 1] random number generator with a fixed seed. Women were ranked by the number drawn. The lowest random draws from eligible women in the household were selected to participate in the survey. The randomly selected women were uploaded to a CommCare survey application for interview by enumerators assigned to each cluster. If there were more than one eligible woman in a selected household, the CommCare application randomly selected one.

### Survey questionnaires (Additional files 1 & 2)

We finalized the questionnaire for the retrospective and prospective assessments using country-specific standard data collection instruments such as the Pakistan demographic and health survey (PDHS). The survey questionnaire covered a range of topics on women’s reproductive health, including marital status, contraceptive knowledge and use, childbearing, and abortion and access to safe abortion services. It sought insights on issues related to uptake, continuation and discontinuation of modern contraceptive methods. The assessments included a contraceptive calendar to collect a month-by-month history of certain key reproductive events (i.e. births, pregnancies, termination of pregnancies, and family planning use) for the five-year calendar period preceding the date of interview. It also captured information on change in contraceptive methods, and reasons for switching and discontinuation [5]. The survey questionnaire, originally devised in English, was translated into Urdu (the national language) and then backtranslated into English for quality assurance purposes. We used a digital data collection process using the CommCare application; this is useful for on-time data entry and quality checking; however, it requires many resources and accessories such as a power bank and internet connectivity for uploading data. We conducted two pilot-tests prior to data collection and modified the questionnaire based on enumerator feedback. In the initial week of data collection, enumerators were asked to complete only one questionnaire per day, which was increased to two questionnaires per day in the second week, and then a minimum of three questionnaires per day for the rest of the survey period.

### Data quality assurance

The study team implemented a number of strategies to ensure quality fieldwork. These included regular quality checks in the CommCare desktop application to identify inconsistencies and outliers in the data. CommCare is a software platform developed by Dimagi Inc. that allows non-programmers to design electronic surveys and run them via mobile applications on android devices. Our application had built-in quality checks to minimize errors and ensure proper skip patterns in the survey. The enumerators were trained to sync completed forms for review by the team leader immediately following each section of the interview. This means that completed survey forms were uploaded to a central server so that the team leader could access the data on her tablet and address any errors in real time. Team leaders also visited a randomly selected 5% sample of households to verify collected information; however, no such discrepancies were found.

We hired all female enumerators and team leaders based on the local norms in Karachi; typically females are permitted to enter the household and talk to women, but males are not. We developed a protocol for re-visiting cases with major errors such as when an enumerator skipped collecting pregnancy-related information even though the respondent had a birth in the last 30 months (as identified in the contraceptive calendar module). The data management unit documented the data cleaning notes and ran rounds of STATA cleaning codes to fix case specific errors following discussions with the field manager, then re-ran the STATA quality checks to confirm that all issues were resolved. In addition, enumerator refresher trainings were conducted weekly to discuss common issues with data and fieldwork, and to review updated field protocols where appropriate. In order to minimize loss to follow up between the household listing and re-visits for the survey, when a selected woman could not be found at their household, the enumerator made three separate attempts to contact her on different days. After each interview or contact attempt, enumerators completed an ‘interviewer contacts’ form in CommCare, which documented the status of each case (e.g. completed, not eligible, moved away, re-scheduled, etc.). To improve response rates, enumerators also scheduled interviews on days/times most convenient for participants (for example, on weekends or evenings, outside of typical working hours).

### Ethical approval

The Ethical Review Committee of the AKU (4964-Ped-ERC-17) and the National Bioethics Committee of Pakistan approved the study. The enumerator read aloud the consent form before asking survey questions to eligible women and signed the consent form on their behalf. The enumerator also provided a hard copy of consent form to the respondent.

## Results

This study documents various issues encountered during data collection and potential solutions. These are broadly categorized into four main themes with further sub-categories (Table 1). The four main themes are: (1) technical issues related to GIS usage and computer assisted personal interviews (CAPI); (2) household listing issues; (3) respondent issues, and (4) field issues.

**Table 1:** Challenges encountered and mitigation measures during a survey of women in urban Karachi

Challenges encountered	Mitigation measures
**1. Technical issues: GIS usage and computer assisted personal interviews (CAPI)**	
**i.** ***GIS Mapping:*** Difficulty in cluster boundary interpretation and household identification at each survey point, especially where dense structures are present.	• Physically validating the boundaries through field visits.
**ii.** Low internet signals in ground floors of tower buildings sometimes made it difficult to sync data.	• Having dedicated staff available during field activities to deal with unexpected tablet issues in real time. • Being able to download data later upon returning to the office.
**iii.** Reporting a technical query and waiting for a solution from staff with access to central server was time consuming.	
**2. Household Listing issues**	
**i.** ***Long duration between household listing and survey***: Enumerators forgot site details and landmarks, respondent migration was higher, door markings were more difficult to identify.	• Started data collection soon after household listing.
**3. Respondent issues**	
**i.** ***Sensitive information:*** Respondents’ perceptions and beliefs make it difficult to discuss some topics. Asking sensitive questions about sexuality can be controversial.	• Refresher training sessions conducted on how to discuss sensitive topics with respondents.
**ii.** ***Security issues*:** Respondents’ fear about child kidnapping and theft and linked it with previous such incidents.	• Took into confidence influential gate keepers in the community and district administration.
**iii.** ***Unwillingness to participation:*** Certain community sub-groups i.e. Urdu speaking and Pashto were reluctant to participate in the study.	• Coordinated with community leaders (especially men) personally; used gatekeeper script.
**iv.** ***Language barriers:*** Some respondents expressed difficulties understanding Urdu language.	• Assigned new enumerators who could communicate in their native language.
**v.** ***Length of questionnaire and participants’ schedules***: Engaging the respondent for an hour or more in a busy personal schedule created barriers to successful data collection.	• Rescheduled such cases as per the availability of the respondents, including weekends and after working hours.
**vi.** ***Participant expectations:*** Many participants expected extra healthcare services, or another way requested material benefits from enumerators.	• Enumerators were trained to explain indirect benefits e.g. sharing research findings with key stakeholders and informing policies that may benefit the respondents in long run.
**4. Field issues**	
**i.** ***Environmental issues:*** Interviews conducted in dark rooms with no electricity, bad odors, dirty streets, in extreme temperatures; enumerators reported feelings of isolation. **ii.** Presence or nearness of family members **iii.** Loud background noises outside	• Worked in groups in neighborhoods where enumerators felt uncomfortable. • Supervisors waited nearby and were available to support if needed. • Scheduled a revisit after confirming another time when most of the family members were away from the home.
**iv**. ***Ensuring privacy:*** Some interviews couldn’t be done inside the house (especially in joint family systems).	• Revisit/reschedule cases as per respondent’s availability.

### Technical issues related to GIS and CAPI

We found that using GIS technology to demarcate clusters, particularly in urban slums, was an efficient approach to establishing a sampling frame; however, its use is subject to issues around cluster boundary interpretation and household identification, particularly for narrow and congested streets. This approach required extra time and additional human resources to validate the boundaries through in-person field visits. We also worked closely with program staff to ensure that the boundaries around areas intended for program implementation were accurately demarcated. This was a time-intensive, but critical step in the process.

Collecting data using electronic devices had many advantages but was also prone to challenges such as power outages, low connectivity in some areas, and slow uploading speed. We ensured that ample tablets were available as backup in such circumstances. When data issues/queries emerged in the field, waiting to receive a response from the technical support team at AKU (who had access to the central server) was a time-consuming procedure. In areas of low connectivity, enumerators struggled with the intermittent internet to sync the data, especially when conducting interviews on ground floors of tower buildings. Nevertheless, the application was programmed such that the survey itself could be run without any connectivity, and syncing could occur later (i.e. each evening upon return to the office). AKU’s dedicated research staff were also on board to deal such queries in the field.

### Household listing issues

Karachi is a metropolitan city comprised of quickly growing urban dwellings and settlements. Residential apartments and multi-story buildings are common. In a dynamic population like our study sample, having a long gap between the household listing and the household survey can lead to high loss-to-follow-up, and recall errors among enumerators, such as forgetting important sites and other neighborhood landmarks. A majority of the housing areas do not have formal addresses, which makes it difficult to identify and track individual households.

To address this challenge, the team marked a unique identification number (ID) on the front door of each household during household listing with the permission of participants. This was helpful to relocate and confirm the households randomized for data collection during the household survey. We trained two separate teams to conduct the household listing and household survey. Initially, our plan was to complete the entire household listing in all selected clusters before beginning the household survey. However, there was a shift in strategy mid-course when the study team identified a need to reduce the time lag between the two activities. In our revised field protocol, the household listing and survey teams worked in tandem so that interviews began immediately after each cluster was listed, rather than listing all clusters before beginning the household survey; this modification proved critical in minimizing loss to follow-up and maintaining an efficient data collection process.

### Respondent issues

The study revealed that respondent issues were the most important barriers to high quality data collection (Table 1). In Karachi, people’s religious beliefs affect their willingness to discuss SRH topics. In addition, perceived security threats and gender-based household dynamics made some women reluctant to participate in surveys. These findings are similar to those reported in other studies [10]. Respondents were initially suspicious of enumerators and viewed them as strangers due to recent thefts and child kidnapping incidents in the neighborhood. We found that discussing sensitive topics such as women’s socioeconomic status (which raised concerns regarding robbery) and sexual and contraceptive history made fieldwork challenging and led to high refusal rates if not approached with sensitivity. Likewise, certain community sub-groups (e.g. Muhajirs and Pashtuns) were reluctant to participate for these reasons. To counter this challenge and to build strong rapport, male field supervisors coordinated with male community leaders personally using a gatekeeper script to initiate the discussion, and we trained an all-female team of enumerators on strategies to improve mutual trust prior to interviews. As a result, refusal rates were low (Jamshed town 9.8%, Yousuf Goth 2.5%, Korangi town 5%, and PIB & Dalmia/Shanti Nagar 4.3%.).

In some instances, respondents expressed difficulties understanding Urdu language and had to be assigned to a new enumerator who could communicate in their native language. To maximize inclusion in our study sites, we ensured that our enumerator team included women who spoke at least one of the four most common languages in Karachi: English, Urdu, Pushto, or Sindhi.

We found that engaging respondents for more than an hour conflicted with women’s busy daily schedules, which created problems for successful data collection. This also increased participant discomfort and unwillingness to participate in the data collection process. In these cases, we rescheduled the remaining portion of the interviews at a time more convenient for respondents (e.g. on Sundays, outside of working hours, etc.).

Another issue this study revealed was that some respondents demanded material benefits, extra healthcare services or sometimes lodged complaints against the healthcare system when visited for surveys, thus enumerators had to be properly trained to deal with such situations. Enumerators were trained to anticipate such demands and explain that a research report will be generated from the study findings and disseminated to concerned stakeholders. This report might inform policies that could benefit respondents in the long run. We also provided a tea mug to all respondents as a token of appreciation for their time and effort.

### Field issues

This study also highlighted the difficulties of conducting long interviews in a dense, urban environment. Our protocol was to conduct the interview privately in women’s homes, but this was sometimes difficult because other family members were present and did not want to leave. Selecting an alternative private interview location was a challenge in some areas since data collection did not take place in a room, particularly in joint family systems, but rather in an open space (for example, at the doorstep where loud background noises and warm temperatures posed additional challenges). In addition, ensuring privacy away from other family members and neighbors was a challenge. We addressed this issue by scheduling a revisit after confirming another time when most family members were away from the home.

The data collection period was affected by some seasonal events such fasting and Eid holidays, and field activities were postponed for 2 months in retrospective sites due to political events and general elections in July, 2018. These data collection protocol adaptations have serious time and budget implications, but we view them as necessary investments to ensure high quality data.

## Discussion

This study demonstrates how conducting surveys in urban Karachi presents unique challenges that can influence the data collection process. However, we found that appropriate mitigation measures to address the environmental and socio-cultural context enabled successful data collection in this setting. Lessons learned may be usefully applied in similar urban settings in other LMICs.

Using GIS technology to demarcate clusters for our sampling frame is a unique and efficient approach of present study. However, the process is time-intensive, as accurate interpretation of cluster boundaries and household identification, particularly within neighborhoods with congested and narrow streets, can be challenging. Working with experienced field teams and program staff who know the area well is critical to success. This finding is consistent with other studies conducted in LMICs, where researchers lacked accurate and detailed spatial images, and encountered challenges tracing the exact location of zoning areas [23, 24]. This unique approach differs from methods used to construct sampling frames in other surveys, such as the 2017–18 Pakistan Demographic and Health Survey (PDHS), which used a complete list of enumeration blocks (EBs) created for the Pakistan Population and Housing Census 2017. The Pakistan Bureau of Statistics (PBS) has formulated EBs into three categories of income groups i.e. low, middle and high, keeping in view the living standard of the majority of the people.

We demonstrate that using electronic data collection methods in LMICs has many advantages; however, a robust technical support protocol must be in place to ensure efficient response from technicians who have access to the central server. This finding is supported by available studies from other LMICs [25, 26], which reported that implementation is challenging both administratively and technically. Such challenges can be reduced by building the technical capacity of enumerators using the training of trainer (ToT) model, where team leaders are trained first, and can oversee data collection and help with troubleshooting to reduce errors during fieldwork.

Our study revealed a number of respondent-related issues during the data collection process. Like other LMICs, we also found considerable seasonal migration among residents, and faced difficulties identifying listed respondents in selected clusters. A study conducted in Bolivia, Kenya, and South Africa showed similar challenges with attrition rates between survey rounds; the interviewers tracked, followed and re-interviewed 84% of women who moved [27]. In our study, coordinating two teams to work in tandem and begin interviews immediately after each cluster’s household listing was completed helped to minimize attrition between data collection activities.

Some respondents were reluctant to participate in our study because discussing family planning, especially use of contraceptive methods and abortion services is highly stigmatized among some Muslim religious groups. However, we also found that highly religious Christian families had similar reservations about discussing such topics. In line with our findings, previous studies report that sexual and reproductive issues are sensitive subjects, rarely discussed in certain communities, and both religious traditions strongly influence the uptake of family planning [28, 29]. This points to the important role of religious leaders as potential agents of change in conservative societies and suggests the need to engage them in efforts to promote sexual and reproductive health.

Respondents’ perceptions around personal security made them reluctant to share personal information in this setting. To reassure respondents and clarify misunderstandings regarding data collection and use, researchers should collaborate with influential gatekeepers in the community and district administration. This finding is consistent with a study in South Africa where respondents perceived enumerators with suspicion, denied access to houses, and viewed study participation as a security risk [30]. Some even complained to local police authorities despite receiving official letters authorizing the research. These issues were exacerbated when enumerators discussed sensitive topics such as family planning and their reproductive histories with respondents of particular religious groups. Another study also reported that both Muslim and Christian groups frequently oppose family planning [31]. To help mitigate these challenges, we recruited female enumerators who were familiar with local social and cultural norms, and provided focused training on strategies to build rapport and trust so that participants felt comfortable sharing sensitive information.

In our study some respondents experienced difficulty understanding Urdu language. We recruited skilled enumerators fluent in multiple native languages. This strategy is endorsed by other research teams who encountered language barriers while collecting data and used bilingual enumerators fluent in local languages [19, 30, 32]. This highlights the importance of recruiting and training a diverse team of enumerators to ensure that all potential respondents have the same ability to communicate with the research team. Misinterpretation of risks and biases could be reduced through training and mock exercises with enumerators.

We adopted a strategy where the training did not stop after the initial two-week session; rather, the team adapted to learnings from the field throughout the data collection process, and jointly developed strategies to address them as they emerged. We held weekly meetings to discuss the progress of every enumerator and reviewed data errors as a team. The field manager identified discrepancies through daily data quality checks and discussed strategies to resolve these queries throughout the data collection period.

The usefulness of household surveys to capture accurate information depends heavily on the survey’s content and length [33]. For example, participants may rush through the data collection process and provide incomplete or inaccurate information. In our study, we rescheduled interviews to avoid this scenario. Furthermore, lengthy questionnaires can lead to enumerator fatigue, but we did not encounter this difficulty in our study. This might be because we did not overburden our enumerators with difficult daily targets [13].

An important learning from our fieldwork was that some respondents expect monetary benefits or other direct benefits such as improved healthcare access. However, the enumerators explained that participants would receive no direct benefits, in keeping with our approved ethical protocol. This finding likely reflects the fact that people receive assistance from many local and international non-governmental organizations (NGOs) involved in charity programs in urban settings. Studies in Pakistan and other LMICs [7, 34, 35] reported similar findings, where both rural and urban communities had strong expectations of unconditional benefit packages when enumerators visit homes. Researchers can show their appreciation by providing a small non-monetary gift (e.g. tea mug) at the end of the interview.

The interview environment is a crucial aspect of the data collection process [2]. Our study found that in some instances, interviews had to be rescheduled to ensure privacy, especially in households with joint families living in crowded spaces. Some respondents understandably felt nervous when discussing sensitive information such their sexual activity and contraceptive use. In this environment, securing a private interview room was a challenge. Previous literature reported that getting access to participants, managing privacy, and obtaining accurate responses to sensitive topics can be difficult [7, 35, 36]. This suggests that in some settings, interviews must be conducted at a neutral, private venue that is suitable and secure for both the respondent and enumerator [2].

To our knowledge, this is the first study to lay out fieldwork issues at different stages of the data collection process in urban environments of Karachi, Pakistan. Our study also had some limitations. First, due to competing household tasks and busy schedules, it is possible that some respondents rushed through the survey. Secondly, we had no way to track respondents who could not be reached after three visits, and were thus unable to capture information on this potentially important sub-group. Finally, we implemented our study by following strict standard protocols to ensure high quality data; this substantially increased the time and cost of data collection. This underlines the need to prioritize high quality data over cost and time complications by planning and budgeting accordingly from the outset substantially increase the time and cost of data.

## Conclusion

Several important lessons can inform future implementation of household surveys in urban populations. First, the use of GIS technology to develop sampling frames is an innovative and cost-effective method in resource-constrained settings like urban Karachi. Second, having a dedicated data management team to monitor electronic data collection in real time facilitates efficient detection of errors and inconsistences, and greatly enhances data quality. Third, the strategy of interviewing women immediately after listing households in each cluster makes it easier to re-locate selected respondents and reduces loss-to-follow up. Fourth, understanding local norms and developing culturally appropriate strategies to engage participants is essential to build trust with communities and may significantly reduce refusals. Training female enumerators preferably from the same locality is ideal since they are can easily build rapport and approach reproductive health topics with sensitivity and respect. Lastly, ensuring privacy in joint family households may be achieved by rescheduling interviews for another time when most family members are not at home. Findings of this study will help to improve the quality and efficiency of future household surveys in urban settings.

## Supplementary Information

**Additional file 1:.** Prospective Survey Questionnaire.

**Additional file 2:.** Retrospective Survey Questionnaire.

## Data Availability

The datasets generated and/or analysed during the current study are available from the corresponding author on reasonable request.

## Abbreviations

GIS: Geographical Information System
SDG: Sustainable Development Goals
LMICs: Low and Middle Income Countries
SRH: Sexual and Reproductive Health
AKU: Aga Khan University
CPR: Contraceptive Prevalence Rate
CAPI: Computer Assisted Personal Interviews) PDHS (Pakistan Demographic and Health Survey
EBs: Enumeration Blocks
