# Supplementary material for: Conducting household surveys on reproductive health in urban settings: lessons from Karachi, Pakistan
Source: BMC Med Res Methodol. 2021 Feb 18;21:38. doi: 10.1186/s12874-021-01216-x (PMC7890958; doi:10.1186/s12874-021-01216-x)
Supplement: Supplementary file 1 — Additional file 1:. Prospective Survey Questionnaire. [file 12874_2021_1216_MOESM1_ESM.pdf]

WILLOWS EVALUATION STUDY - PAKISTAN

WOMEN'S BASELINE SURVEY, VERSION 1.0

PAKISTAN | | | | | | | | | | | | | | | | | | |

Principal Investigator: Dr Sajid Soofi, The Aga Khan University, Pakistan

بنیادی تحقیق کار: ڈاکٹر ساجد بشیرصوفی، آغا خان یونیورسٹی کراچی، پاکستان

CONSENT

INSTRUCTIONS: TAKE OUT A PAPER COPY OF THE BASELINE WOMEN CONSENT FORM. READ THE CONSENT FORM ALOUD, OR ASK THE WOMAN TO READ THROUGH IT. AT THE END OF THE CONSENT FORM, WHEN IT IS TIME TO COLLECT THE RESPONDENT'S SIGNATURE, ASK THE FOLLOWING QUESTION:

بیس لائن سروے کی ایک کاغذی کاپی نکال کر بلند آواز سے اجازت نامہ کے فارم کو پڑھیں،یا خاتون سے کہیں کے وہ خود اس کو پڑھیں۔ اجازت نامہ کے فارم کے اختتام پر جب خاتون جواب دہندہ انٹرویو کے لیے رضامندی کا اظہار کرے تو دستخط لینے سے پہلے یہ سوالات پوچھیں۔

CO1	Do you consent to participate in this study?<br>کیا آپ اس مطالعہ میں شرکت کرنا چاہتی ہیں؟	YES ہاں<br>NO نہیں	.... 1<br>........................... 2	 → END

CONSENT FORM SIGNATURES

DAY		MONTH		YEAR			
				2	0	1	

RESPONDENT SIGNATURE OR THUMBPRINT جواب دہندہ کے دستخط یا انگو ٹھے کا نشان

SIGNATURE OF RESPONDENT INDICATES INFORMED CONSENT WAS PROVIDED.

جواب دہندہ کے دستخط یا انگوٹھے کا نشان اس بات کا ثبوت ہے کہ جواب دہندہ مطالعہ میں شمولیت کے لیے رضامند ہیں۔

DAY		MONTH		YEAR			
				2	0	1	

ENUMERATOR SIGNATURE (انٹرویو لینے والے) کے دستخط

SIGNATURE OF ENUMERATOR CONFIRMS THAT INFORMED CONSENT WAS PROVIDED.

(انٹرویو لینے والے) کے دستخط اس بات کا ثبوت ہوں گے کہ معلوماتی اجازت نامہ کا فارم مستند ہے۔

RESPONDENT IDENTIFICATION

جواب دہندہ کی نشاندہی

CLUSTER NUMBER کلسٹر نمبر .... [ ][ ][ ][ ][ ]
(Select at the time of Case Selection)

HOUSEHOLD ADDRESS گھرانہ کا ایڈرس ______________________________
(Display automatically from listing info.) ______________________________

RESPONDENT CONTACT INFORMATION

جواب دہندہ سے رابطہ کی معلومات

FULL NAME OF WOMAN
خاتون کا پورا نام: ______________________________

HUSBAND NAME OF THE WOMAN
خاتون کے شوہر کا نام: ______________________________

PHONE NO. OF HUSAND
شوہر کا فون نمبر: ______________________________

INTERVIEWER CONTACTS/VISITS

انٹرویو لینے والے کے رابطوں/دوروں کی تعداد

	1	2	3	FINAL CONTACT<br>حتمی رابطہ
DATE تاریخ:<br><br>INTERVIEWER'S NAME<br>انٹرویو لینے والے کا نام:<br>RESULT* نتیجہ:	______<br><br>______<br>______	______<br><br>______<br>______	______<br><br>______<br>______	DAY دن: [ ][ ]<br>MONTH مہینہ: [ ][ ]<br>YEAR سال: [ ][ ][ ][ ]<br>INT. NO. [ ][ ][ ]<br>RESULT* نتیجہ: [ ]
NEXT CONTACT: اگلا رابطہ:<br>DATE تاریخ:<br>TIME وقت:	 ______<br>______	 ______<br>______		رابطوں کی مجموعی تعداد<br>TOTAL NUMBER OF CONTACTS [ ]

*RESULT CODES:
نتیجہ کے کوڈز:

1 COMPLETED	مکمل 1	4 REFUSED	انکار کر دیا 4
2 NOT AVAILABLE	دستیاب نہیں 2	5 PARTLY COMPLETED	جزوی طور پر مکمل 5
3 POSTPONED	ملتوی کر دیا 3	6 INCAPACITATED	معذور 6

کچھ اور 7
7 OTHER ______________________________
SPECIFY

LANGUAGE OF QUESTIONNAIRE** [ ][ ]
سولانامہ میں استعمال کی گئی زبان

LANGUAGE OF INTERVIEW** [ ][ ]
انٹرویو میں استعمال کی گئی زبان

NATIVE LANGUAGE OF RESPONDENT** [ ][ ]
جواب دہندہ کی مقامی زبان

LANGUAGE OF QUESTIONAIRE 01 URDU 02 ENGLISH

LANGUAGE WOMEN SPEAKS AT HOME** ....
خاتون جو زبان گھر میں بولتی ہے۔

** URDU=1	PUNJABI=2	SINDHI=3	PUSTO=4	BALUCHI= 5	ENGLISH=6	BARUHI=7	SARAIKI=8
اردو	پنجابی	سندھی	پشتو	بلوچی	انگلش	براحوی	سرائیکی

OTHER SPECIFY دیگر وضاحت کریں ______________________________

INTRODUCTION

In this survey I will ask you questions about your health and well-being. The questions in this survey usually take less 60 minutes. All of the answers you give will be confidential and will not be shared with anyone other than members of our research team that will analyze the information collected from all women together. You don't have to be in the survey, but we hope you will agree to answer the questions since your views are important. If I ask you any question you don't want to answer, just let me know and I will go on to the next question or you can stop the interview at any time.

اس سروے میں ہم آپ اور آپکی صحت سے متعلق سوالات پوچھیں گے۔ جس میں عام طور پر 60 منٹ سے کم وقت لگ سکتے ہیں۔ آپ کے دئیے گئے تمام جوابات کو انتہائی راز داری میں رکھا جائے گا اور اس کی شراکت داری کسی بھی غیر متعلقہ شخص سے نہیں کی جائے گی ماسوائے اس ریسرچ کے ممبران کے ۔آپ کی اس سروے میں شمولیت صرف اتنی ہے کہ آپ رضامندی کے ساتھ جوابات دیں جو کہ صحت کے حوالے سے انتہائی اہم ہیں۔اگر آپ اس سروے میں حصہ نہیں لیتی ، تب بھی ہم یہ امید کرتے ہیں کہ آپ ان سوالوں کے جوابات دینے کے لئے رضا مند ہو جائینگی کیوں کہ آپ کی رائے ہمارے لئے بہت اہم ہے۔ اگر آپ کسی سوال کا جواب دینا نہ چاہیں تو منع کرسکتی ہیں اپ سے اگلے سوال پوچھ لیں جائیں گے یا پھر آپ چاہیں تو مکمل طور پر انٹرویو بھی روک سکتی ہیں۔

NO.	QUESTIONS AND FILTERS	CODING CATEGORIES	SKIP
001	RECORD THE TIME INTERVIEW BEGINS.<br>انٹرویو کو شروع کرتے وقت کا ٹائم ریکارڈ کریں<br>____________________	HOURSگھنٹے [ ][ ]<br>MINUTESمنٹس [ ][ ]	

1. HOUSEHOLD SCHEDULE

						IF AGE 15 OR OLDER	IF AGE 5 YEARS OR OLDER		IF AGE 5- 30YEARS		IF AGE 0-4 YEARS
LINE NO.	USUAL RESIDENTS NAMES<br>معمول کے رہایش پزیر افراد کے نام	RELATIONSHIP TO THE RESPONDENT<br>(نام) کا گھرجوابدہندہ	SEX<br>جنس	YEAR OF BIRTH<br>پیدائش کا سال	AGE<br>عمر	MARITAL STATUS<br>ازواجی حیثیت	EVER ATTENDED SCHOOL<br>کبھی بھی اسکول گئے ہوں		CURRENT/RECENT SCHOOL ATTENDANCE<br>حالیہ اسکول جارہے ہوں		BIRTH REGISTRATION<br>پیدائش کی رجسٹریشن
101	102	103	104	105	106	107	108	109	110	111	112
	Please give me the names of the persons who usually live in your household, starting with yourself.<br>برائے مہربانی مجھے عام طور پر آپ کے گھر میں رہنے والوں کے نام بتائیں، آپ اپنے آپ سے شروعات کریں۔<br>تمام تفصیلات لینے کے بعد لسٹ بنالیں جس میں رشتہ ،جنس ہر فرد کی پوچھ کر سوال 113 113C اورA بھریں،واضح رہے کہ لسٹ مکمل ہو۔ پھر تمام مناسب سوالات جو 105 سے 112 تک ہیں ہر فرد کے لیے علیحدہ بھریں۔<br>AFTER LISTING THE NAMES AND RECORDING THE RELATIONSHIP AND SEX FOR EACH PERSON, ASK QUESTIONS 113A-113C AT THE END OF THE LISTING SECTION TO BE SURE THAT THE LIST IS COMPLETE THEN ASK APPROPRIATE QUESTIONS 105-112 FOR EACH PERSON.	What is the relationship of (NAME) to the head of the household?<br>(نام) کا گھر کے سربراہ سے کیا رشتہ ہے<br>SEE CODES BELOW.<br>کوڈ کے لیے نیچے دیکھیں	Is (NAME) male or female?<br>کیا (نام) مرد ہے یا عورت؟	In what year was (NAME) born?<br>(نام) کونسے سال میں پیدا ہواتھا/تھی ؟	How old was (NAME) on his/her last birthday?<br>(نام) کی آخری سالگرہ پر کیا عمر تھی؟	What is (NAME)'s current marital status?<br>1 = MARRIED<br>2 = DIVORCED/ SEPARATED<br>3 = WIDOWED<br>4 = NEVER-MARRIED<br>8 = DON'T KNOW<br>9 = REFUSED<br>(نام) کی ازواجی حیثیت<br>شادی شدہ 1<br>طلاق 2 یافتہ/علیحدہ<br>بیوہ 3<br>کبھی شادی نہیں کی 4<br>انکار کردیا-9<br>معلوم نہیں 8<br>انکار کردیا -9	Has (NAME) ever attended school?<br>کیا (نام) کبھی بھی سکول گی/گئے ہیں؟ نیچے کوڈ دیکھیں ۔ اگر جواب نہیں ہے تو سوال نمبر 112 پر جائے	What is the highest level of school (NAME) has attended?<br>SEE CODES BELOW.<br>What is the highest grade (NAME) completed at that level?<br>SEE CODES BELOW.<br>سب سے بڑی کلاس کون سی تھی (نام) نے کون سی آخری کلاس پڑھی تھی؟	Did (NAME) attend school at any time during the (2016-2017) school year?<br>کیا (نام) نے کبھی 2016 سے 2017 کے درمیان اسکول میں پڑھا ہے؟	During this/that school year, what level and grade [is/was] (NAME) attending?<br>اس دوران (نام) نے کون سا گریڈ /کلاس پڑھی ہے تھی؟<br>SEE CODES BELOW.<br>نیچے کوڈ دیکھیں	Does (NAME) have a birth certificate?<br>اگر نہیں تو تفتیش کرےکیا (نام) کا کوئی پیدائشی سرٹیفیکٹ ہے<br>IF NO, PROBE:<br>Has (NAME)'s birth ever been registered with the civil authority?<br>کیا (نام) کی کبھی بھی سول اتھاڑی میں رجسٹریشن ہے<br>1 HAS CERTIFICATE OBS.<br>2 HAS CERTIFICATE NOT OBS.<br>3 REGISTERED<br>4 NEITHER<br>5 DON'T KNOW<br>1 سرٹیفکیٹ دیکھا<br>2 سرٹیفکیٹ نہیں<br>3 رجسٹرڈ ہے<br>4 کبھی نہیں<br>5 معلوم نہیں
			M F	YEAR	IN YEARS		Y N	LEVEL GRADE	Y N	LEVEL GRADE	
01			1 2				1 2 NEXT LINE		1 2 NEXT LINE		
02			1 2				1 2 NEXT LINE		1 2 NEXT LINE		
03			1 2				1 2 NEXT LINE		1 2 NEXT LINE		
04			1 2				1 2 NEXT LINE		1 2 NEXT LINE		
05			1 2				1 2 NEXT LINE		1 2 NEXT LINE		
06			1 2				1 2 NEXT LINE		1 2 NEXT LINE		
07			1 2				1 2 NEXT LINE		1 2 NEXT LINE		
08			1 2				1 2 NEXT LINE		1 2 NEXT LINE		
11			1 2				1 2 NEXT LINE		1 2 NEXT LINE		
12			1 2				1 2 NEXT LINE		1 2 NEXT LINE		
13			1 2				1 2 NEXT LINE		1 2 NEXT LINE		
14			1 2				1 2 NEXT LINE		1 2 NEXT LINE		
15			1 2				1 2 NEXT LINE		1 2 NEXT LINE		

1. HOUSEHOLD SCHEDULE

LINE NO.	USUAL RESIDENTS NAMES<br>معمول کے رہایش پزیر افراد کے نام	RELATIONSHIP TO THE RESPONDENT<br>(نام) کا گھر جوابدہندہ	SEX<br>جنس	YEAR OF BIRTH<br>پیدائش کا سال	AGE<br>عمر	IF AGE 15 OR OLDER<br>MARITAL STATUS<br>ازواجی حیثیت	IF AGE 5 YEARS OR OLDER<br>EVER ATTENDED SCHOOL<br>کبھی بھی اسکول گئے ہوں		IF AGE 5-30YEARS<br>CURRENT/RECENT SCHOOL ATTENDANCE<br>حالیہ اسکول جارہے ہوں		IF AGE 0-4 YEARS<br>BIRTH REGISTRATION<br>پیدائش کی رجسٹریشن
101	102	103	104	105	106	107	108	109	110	111	112
	Please give me the names of the persons who usually live in your household, starting with yourself.<br>برائے مہربانی مجھے عام طور پر آپ کے گھر میں رہنے والوں کے نام بتائیں، آپ اپنے آپ سے شروعات کریں۔<br>تمام تفصیلات لینے کے بعد لسٹ بنائیں جس میں رشتہ ،جنس ہر فرد کی پوچھ کر سوال 113 113C اورA بھریں۔واضح رہے کہ لسٹ مکمل ہو۔ پھر تمام مناسب سوالات جو 105 سے 112 تک ہیں ہر فرد کے لیے علیحدہ بھریں۔<br>AFTER LISTING THE NAMES AND RECORDING THE RELATIONSHIP AND SEX FOR EACH PERSON, ASK QUESTIONS 113A-113C AT THE END OF THE LISTING SECTION TO BE SURE THAT THE LIST IS COMPLETE THEN ASK APPROPRIATE QUESTIONS 105-112 FOR EACH PERSON.	What is the relationship of (NAME) to the head of the household?<br>(نام) کا گھر کے سربراہ سے کیا رشتہ ہے<br>SEE CODES BELOW.<br>کوڈ کے لیے نیچے دیکھیں	Is (NAME) male or female?<br>کیا (نام) مرد ہے یا عورت؟	In what year was (NAME) born?<br>(نام) کونسے سال میں پیدا ہوا تھا/تھی ؟	How old was (NAME) on his/her last birthday?<br>(نام) کی آخری سالگرہ پر کیا عمر تھی؟	What is (NAME)'s current marital status?<br>1 = MARRIED<br>2 = DIVORCED/ SEPARATED<br>3 = WIDOWED<br>4 = NEVER-MARRIED<br>8 = DON'T KNOW<br>9 = REFUSED<br>(نام) کی ازواجی حیثیت<br>شادی شدہ 1<br>طلاق 2<br>یافتہ/علیحدہ<br>بیوہ 3<br>کبھی شادی نہیں کی 4<br>انکار کردیا-9<br>معلوم نہیں 8<br>انکار کردیا -9	Has (NAME) ever attended school?<br>کیا (نام) کبھی بھی سکول گئ/گئے ہیں؟ نیچے کوڈ دیکھیں ۔ اگر جواب نہیں ہے تو سوال نمبر 112 پر جائیے۔	What is the highest level of school (NAME) has attended?<br>SEE CODES BELOW.<br>What is the highest grade (NAME) completed at that level?<br>SEE CODES BELOW.<br>سب سے بڑی کلاس کون سی تھی (نام) نے کون سی آخری کلاس پڑھی تھی؟	Did (NAME) attend school at any time during the (2016-2017) school year?<br>کیا (نام) نے کبھی 2016 سے 2017 کے درمیان اسکول میں پڑھا ہے؟	During this/that school year, what level and grade [is/was] (NAME) attending?<br>اس دوران (نام) نے کون سا گریڈ /کلاس پڑھی ہے تھی؟<br>SEE CODES BELOW.<br>نیچے کوڈ دیکھیں ۔	Does (NAME) have a birth certificate?<br>اگر نہیں تو تفتیش کریے کیا (نام) کا کوئی پیدائشی سرٹیفیکٹ ہے<br>IF NO, PROBE:<br>Has (NAME)'s birth ever been registered with the civil authority?<br>کیا (نام) کی کبھی بھی سول اتھاڑی میں رجسٹریشن ہے<br>1 HAS CERTIFICATE OBS.<br>2 HAS CERTIFICATE [illegible]<br>3 REGISTERED<br>4 NEITHER<br>5 DON'T KNOW<br>سرٹیفکیٹ دیکھا 1<br>سرٹیفکیٹ نہیں 2<br>رجسٹرڈ ہے 3<br>کبھی نہیں 4<br>معلوم نہیں 5
16			1 2				1 2 → NEXT LINE		1 2 → NEXT LINE		
17			1 2				1 2 → NEXT LINE		1 2 → NEXT LINE		
18			1 2				1 2 → NEXT LINE		1 2 → NEXT LINE		
19			1 2				1 2 → NEXT LINE		1 2 → NEXT LINE		
20			1 2				1 2 → NEXT LINE		1 2 → NEXT LINE		

TICK HERE IF CONTINUATION SHEET USED ☐

113A) Just to make sure that I have a complete listing: are there any other persons such as small children or infants that we have not listed?

YES

ت کا یقین کر لیں کہ میں نے لسٹنگ مکمل کر لی ہے، کیا کوئی ایسا فرد یا بچہ ہے جو اس لسٹ میں موجو

CODES FOR Q. 103: RELATIONSHIP TO HEAD OF HOUSEHOLD

01 = HEAD	08 = BROTHER OR SISTER بہن/ بھائی
02 =HUSBAND شوہر	09 = OTHER RELATIVE دیگر رشتہ دار
03 = SON OR DAUGHTER بیٹی	10 = ADOPTED/FOSTER/ گود لیا ہو /رضاعی/
04 = SON-IN-LAW OR DAUGHTER-IN-LAW	STEPCHILD سوتیلی اولاد
	11 = NOT RELATED کوئی رشتہ نہیں
05 = GRANDCHILD نواسے /	88 = DON'T KNOW معلوم نہیں
06 = PARENT	99 = REFUSED انکار کردیا
07 = PARENT-IN-LAW سرالی	

CODES FOR Qs. 109 AND 111: EDUCATION

LEVEL	GRADE	
1 = PRIMARY پرائمری	00 = LESS THAN 1 YEAR COMPLETED	08 = CLASS 8
2 = MIDDLE مڈل	01 = CLASS 1	09 = CLASS 9
3 = SECONDARY میٹرک	02 = CLASS 2	10 = MATRIC, CLASS 10
4 = HIGHER ہائیر	03 = CLASS 3	11 = CLASS 11
8 = DON'T KNOW معلوم	04 = CLASS 4	16= MASTER'S DEGREE OR MBBS,PhD, MPHIL, BSc (4 YEARS)
9 = REFUSED انکار	05 = CLASS 5	88 = Don't know
	06 = CLASS 6	99 = Refused
	07 = CLASS 7	

2. HOUSEHOLD CHARACTERISTICS

NO.	QUESTIONS AND FILTERS	CODING CATEGORIES	SKIP
201	Now I would like to ask you questions about your household. What is the main source of drinking water for members of your household?<br>اب میں آپ کے گھر کے بارے میں آپ سے سوالات کرنا چاہونگی۔<br>میں گھر کے افراد کے لئے پینے کے پانی کا سب سے اہم ذریعہ کیا ہے؟	1 PIPED WATER — عام سپلائی /لائن کا پانی 1<br>2 WELL OR BOREHOLE — کنواں /زیرزمین پانی 2<br>3 TANKER TRUCK/CART — ٹینکر /ٹرک/گاڑی(مثلاً گدھاگاڑی سےپانی کی فراہمی) 3<br>4 RAIN/SURFACE WATER (RIVER/DAM) — بارش /سطح کا پانی(دریا/ڈیم) 4<br>5 BOTTLED/SACHET WATER — بوتل کا پانی 5<br>96 OTHER (SPECIFY) — دیگر وضاحت 96<br>99 REFUSED — انکار کردیا 99	
202	What kind of toilet facility do members of your household usually use?<br>گھر کے افراد عام طور پر کس قسم کا لٹیرین استعمال کرتے ہیں؟	1 FLUSH OR POUR FLUSH TOILET — فلش سیوریج کے نظام سے جڑا ہوا ہے 1<br>2 PIT LATRINE — گڑھے والا لیٹرین 2<br>3 BUCKET TOILET — بالٹی /ٹب والا لیٹرین 3<br>4 PUBLIC TOILET (KVIP) — سرکاری /شہریوں کے لیے بنا لیٹرین 4<br>5 NO FACILITY/BUSH/FIELD/SEA — کوئی سہولت نہیں/ کھلے میدان میں /جھاڑیوں میں /سمندر میں 5<br>96 OTHER (SPECIFY) — دیگر وضاحت کریں 96<br>99 REFUSED — انکار کردیا 99	5 → 205
203	Do you share this toilet facility with other households?<br>کیا آپ کے گھر کا لیٹرین دوسرے گھرانے کے افراد بھی استعمال کرتے ہیں؟	1 YES — ہاں 1<br>2 NO — نہیں 2<br>99 REFUSED — انکار کر دیا 99	2 → 205
204	How many households use this toilet facility?<br>کتنے گھرانے اس لیٹرین کو استعمال کرتے ہیں؟	NO. OF HOUSEHOLDS — گھروں کی تعداد 0 [ ][ ]<br>88 DON'T KNOW — معلوم نہیں 88<br>99 REFUSED — انکار کردیا 99	

205 Does your household have: کیا آپ کے گھر میں مندرجہ زیل سہولیات ہیں:

			ہاں YES	نہیں NO	معلوم نہیں DK	انکار کردیا RF
1	ELECTRICITY	بجلی 1	1	2	88	99
2	RADIO	ریڈیو 2	1	2	88	99
3	TELEVISION	ٹی وی 3	1	2	88	99
4	A cellular/mobile phone?	موبائل فون 4	1	2	88	99
5	HOW MANY CELL PHONES?	موبائل فونز کی تعداد 5	[ ][ ]		88	99
6	A telephone (landline)?	تار والا زمینی فون 6	1	2	88	99
7	A refrigerator?	ریفریجرٹر 7	1	2	88	99
8	Almirah/Cabinet?	الماری/کیبنٹ 8	1	2	88	99
9	Chair?	کرسی 9	1	2	88	99
10	Room cooler?	روم کولر 10	1	2	88	99
11	Air conditioner?	ایئرکنڈیشنر 11	1	2	88	99
12	Washing machine?	کپڑے دھونے کی مشین 12	1	2	88	99
13	Water pump?	پانی کی موٹر 13	1	2	88	99
14	Bed?	پلنگ 14	1	2	88	99
15	A wall clock?	گھڑی 15	1	2	88	99
16	Sofa?	صوفہ 16	1	2	88	99
17	Camera?	کیمرہ 17	1	2	88	99
18	Sewing machine?	سلائی مشین 18	1	2	88	99
19	Computer?	کمپوٹر 19	1	2	88	99
20	Internet connection?	انٹر نیٹ کنکشن 20	1	2	88	99

NO.	QUESTIONS AND FILTERS	CODING CATEGORIES	SKIP
206	What type of fuel does your household mainly use for cooking?<br>آپ کے گھر میں عام طور پرکھانا پکانے کے لیے کونسا ایندھن استعمال ہوتا ہے؟	1 ELECTRICITY OR GAS — بجلی یا گیس 1<br>2 WOOD, CROP RESIDUE, SAWDUST, OR ANIMAL WASTE — لکڑی، فصل کے باقیات، لکڑی کا چورہ یا جانوروں کا گوبر ، 2<br>3 KEROSENE OR CHARCOAL — مٹی کا تیل/ لکڑی کوئلہ 3<br>95 NO FOOD COOKED IN HOUSEHOLD — گھر میں کھانا نہیں بنتا 95<br>96 OTHER (SPECIFY) — دیگر وضاحت 96<br>99 REFUSED — انکار کردیا 99	95 → 209
207	Is the cooking usually done in the house, in a separate building, or outdoors?<br>کیا عام طور پر کھانا گھر کے اندر تیار ہوتا ہے؟یا الگ سے کسی عمارت میں ، یا گھر کے باہر کسی اور جگہ؟	1 IN THE HOUSE — گھر میں 1<br>2 IN A SEPARATE BUILDING — علیحدہ گھر میں 2<br>3 OUTDOORS — گھر سے باہر 3<br>96 OTHER (SPECIFY) — دیگر وضاحت کریں 96<br>99 REFUSED — انکار کر دیا 99	→ 209
208	Do you have a separate room which is used as a kitchen?<br>کیاآپ کے گھر میں کھانا بنانے کے لئے علیحدہ سے کمرہ ہے جسے (باورچی خانہ)کے طور پر استعمال کیا جا تا ہے؟	1 YES — ہاں 1<br>2 NO — نہیں 2<br>99 REFUSED — انکار کردیا 99	
209	MAIN MATERIAL OF THE FLOOR.<br>فرش میں استعمال ہونے والا میٹریل :<br>RECORD OBSERVATION, IF POSSIBLE OR ENQUIRE WHEN OBSERVATION IS NOT POSSIBLE<br>اگر ممکن ہوتومشاہدہ کریں<br>اگر ممکن نہ ہو تو معلومات حاصل کریں۔	1 NATURAL FLOOR (EARTH/SAND/ MUD OR DUNG) — قدرتی فرش (زمین/ریت/مٹی/چکنی مٹی یا گوبر) 1<br>2 RUDIMENTARY FLOOR (WOOD PLANK/ PALM/BAMBOO) — ہموار فرش/پالش والا لکڑی کا فرش/ کجھور کے پتے/بانس 2<br>3 FINISHED FLOOR (CEMENT/ CERAMIC/CARPET/VINYL/TILES) — مکمل فرش)سیمنٹ/ سیرامک ٹائل/کارپیٹ/ ویناٸل 3<br>96 OTHER (SPECIFY) — دیگر وصاحت 96<br>99 NOT OBSERVED / NOT REPORTED — مشاہدہ نہیں کیا/رپورٹ نہیں کیا 99	

No.	Question	Response	Skip
210	MAIN MATERIAL OF THE ROOF<br>گھرکی چھت پر استعمال ہونے والا میٹریل:<br>RECORD OBSERVATION, IF POSSIBLE<br>OR ENQUIRE WHEN OBSERVATION IS NOT POSSIBLE<br>اگر ممکن ہوتومشاہدہ کریں<br>اگر ممکن نہ ہو تو معلومات حاصل کریں-	1 NATURAL ROOFING (NONE OR THATCH/PALM LEAF OR SOD/GRASS) کوئی چھت نہیں/ کھجور کے پتے /خشک پتے 1<br>2 RUDIMENTARY ROOFING (RUSTIC MAT/PALM/BAMBOO/ WOOD PLANKS/CARDBOARD) سرکنڈی /گھانس ابتدائی /بنیادی چھت جمانے والے میٹ کھجور کی ننڈیاں/بانس/ لکڑی کے تختے 2<br>3 FINISHED ROOFING (METAL/TILE/CERAMIC/CEMENT) صاف ستھری بنی چھت/ فولادی شیٹ /ٹائلز/سریہ سیمنٹ سے بنی چھت 3<br>96 OTHER (SPECIFY) دیگر وضاحت کریں 96<br>99 NOT OBSERVED / NOT REPORTED مشاہدہ نہیں کیا/رپورٹ نہیں کیا 99	
211	MAIN MATERIAL OF THE EXTERIOR WALLS.<br>بیرونی دیواروں کا بنیادی میٹریل:<br>RECORD OBSERVATION, IF POSSIBLE<br>OR ENQUIRE WHEN OBSERVATION IS NOT POSSIBLE<br>اگر ممکن ہوتومشاہدہ کریں<br>اگر ممکن نہ ہو تو معلومات حاصل کریں-	1 NATURAL WALLS (NONE/CANE/PALM/TRUNKS/DIRT/MUD/ BAMBOO/STICKS/MUD) قدرتی دیوار) کوئی دیوار نہیں/ بانس /کھجور کی بانس لکڑی کے تنے/ مٹی/ 1<br>2 RUDIMENTARY WALLS (UNBAKED BRICKS/MUD/CARTON /PLASTIC/BAMBOO/STONE/UNCOVERED/PLYWOOD/CARDBOARD /REUSED WOOD) بنیادی /ابتدائی دیوار)کچی اینٹیں/مٹی/ گتے /پلاسٹک پتھر مٹی کے ساتھ مکمل کھلا ہوا موٹے گتے استعمال شدہ لکڑی 2<br>3 FINISHED WALLS (BAKED BRICKS/TENT/STONE WITH LIME/ CEMENT/BRICKS/COVERED ADOBE/WOOD PLANKS/SHINGLES) مکمل صاف بنی دیوار) پکی اینٹیں/ ٹینٹ/ پتھر چونے یا سیمنٹ کے ساتھ/ سیمنٹ/ اینٹیں/ مکمل طور پر احاطہ کی ہوئی لکڑی کے تختے /دیوار سازی/ لکڑی کے تختے 3<br>96 OTHER (SPECIFY) دیگر وضاحت کریں 96<br>99 NOT OBSERVED/NOT REPORTED مشاہدہ نہیں کیا/رپورٹ نہیں کیا 99	
212	How many rooms in this household are used for sleeping?<br>اس گھر میں سونے کے لیے کتنے کمرے استعمال ہوتے ہیں؟	ROOMS کمرے [ ][ ]<br>NOT OBSERVED / NOT REPORTED مشاہدہ نہیں کیا/رپورٹ نہیں کیا 99	
213	Does any member of this household own:<br>کیا گھر کےکسی بھی فرد کے پاس یہ چیزیں موجود ہیں:	YES NO ہاں نہیں	
	1 A watch? گھڑی 1	1 2 1 2	
	2 A bicycle? سائیکل 2	1 2 1 2	
	3 A motorcycle or motor scooter? موٹر سائیکل/موٹرسکوٹر 3	1 2 1 2	
	4 A motorking? موٹر کنگ 4	1 2 1 2	
	5 An animal-drawn cart (oxcart)? جانور سے جڑی گاڑی (مثلاً گدھا گاڑی) 5	1 2 1 2	
	6 A car or truck? کار/ٹرک/بس 6	1 2 1 2	
	7 A boat with a motor? موٹر والی بوٹ 7	1 2 1 2	
	8 A boat without a motor? بغیر موٹر والی بوٹ 8	1 2 1 2	
214	Does any member of this household own any agricultural land?<br>کیا اس گھر کے کسی فرد کی ملکیت میں زرعی زمین ہیں؟	1 YES ہاں 1<br>2 NO نہیں 2<br>88 DON'T KNOW معلوم نہیں 88<br>99 REFUSED انکار کردیا 99	2, 88, 99 → 216
215	How much agricultural land do members of this household own?<br>اگر ہاں تو اس گھر کے افراد کی ملکیت میں کتنی زرعی زمین ہے؟<br>RECORD IN UNITS RESPONDENT USES.<br>یونٹ میں درج کریں -<br>IF 99.5 OR MORE ACRES, RECORD IN HECTARES<br>100 ACRES= 1 HECTARE<br>اگر 99.5 یا اس سے زیادہ ایکڑز، توہیکٹرز میں درج کریں<br>100ایکڑز ہیکٹر<br>IF 95 OR MORE HECTARES, CIRCLE '9995'.<br>اگر 95 یا اس سے زیادہ ہیکٹرز، تو9995 کے گرد دائرہ بنائیں	HECTARES ہیکٹرز 1 [ ][ ].[ ]<br>ACRES ایکڑز 2 [ ][ ].[ ]<br>PLOTS پلاٹ 3 [ ][ ].[ ]<br>95 OR MORE HECTARES 95یااس سے زیادہ ہیکٹررز ###<br>DON'T KNOW معلوم نہیں ###<br>REFUSED انکار کردیا ###	
216	Does any member of this household have a bank account?<br>کیا اس گھرانے کے کسی فرد کا بینک اکاؤنٹ ہے ؟	1 YES ہاں 1<br>2 NO نہیں 2<br>88 DON'T KNOW معلوم نہیں 88<br>99 REFUSED انکار کردیا 99	
217	Does someone in your household own your home?<br>کیا اس گھر کا مالک آپ کے گھرانے کا کوئی فرد ہے؟	1 YES ہاں 1<br>2 NO نہیں 2<br>88 DON'T KNOW معلوم نہیں 88<br>99 REFUSED انکار کردیا 99	

3. RESPONDENT'S BACKGROUND

NO.	QUESTIONS AND FILTERS	CODING CATEGORIES	SKIP
303	In which region or country were you born?<br>آپ کس ملک یا ملک علاقے میں پیدا ہوئی تھی؟	1 PUNJAB پنجاب 1<br>2 SINDH سندھ 2<br>3 KHYBER PUKHTUNKHAWA خیبر پختونخواں 3<br>4 BALOCHISTAN بلوچستان 4<br>5 GILGIT-BALTISTAN گلگت بلتستان 5<br>6 FATA فاٹا 6<br>7 Azad Kashmir آزاد کشمیر 7<br>8 Afghanistan افغانستان 8<br>9 Bangladesh بنگلہ دیش 9<br># India انڈیا 10<br># Other Country specify دیگر وضاحت 11<br>99 REFUSED انکار کردیا 99	
304	For how long have you resided in this neighborhood/area?<br>آپ کتنے عرصے سےاس محلے/علاقے میں یا اس سے ملحق علاقے میں رہ رہی ہیں ؟	MONTHS مہینہ [ ][ ]<br>YEARS سال [ ][ ]<br>DON'T KNOW ....معلوم نہیں 888<br>REFUSED ....انکار کردیا 999	
305	What is your religion?<br>آپکا تعلق کس مذہب سے ہے؟	1 ISLAM اسلام 1<br>2 HINDUISM ہندو 2<br>3 CHRISTIANITY عیسائیت 3<br>4 BUDDHISM بدھ مت 4<br>5 TRADITIONAL/SPIRITUALIST روایتی روحانی پیشوہ 5<br>6 NO RELIGION کوئی مذہب نہیں 6<br>96 OTHER (SPECIFY) دیگر وضاحت کریں 96<br>99 REFUSED انکار کردیا 99	
PK_3_1	How often do you attend religious services?<br>آپ اپنی مذہبی رسومات اکثر کتنی بار ادا کرتی ہیں	1 MORE THAN ONCE PER WEEK ہفتے میں ایک سے زیادہ مرتبہ 1<br>2 ONCE PER WEEK ہفتے میں ایک بار 2<br>3 NEARLY EVERY WEEK تقریباً ہر ہفتے 3<br>4 2-3 TIMES PER MONTH مہینے میں دو سے تین مرتبہ 4<br>5 ABOUT ONCE PER MONTH مہینے میں ایک دفعہ 5<br>6 SEVERAL TIMES PER YEAR سال میں کئی بار 6<br>7 ONCE OR TWICE A YEAR سال میں ایک یا دو بار 7<br>8 LESS THAN ONCE A YEAR سال میں ایک بار سے بھی کم 8<br>9 NEVER کبھی نہیں 9<br>99 REFUSED انکار کردیا 99	→306
PK_3_2	How often do you offer your prayer?<br>اکثر آپ نماز کتنی مرتبہ ادا کرتی ہیں؟<br>**ONLY ASKED IF 305 = 1**<br>صرف پوچھیں اگر 305 کا جواب `1 ہے	ONCE PER DAY.......................................دن میں ایک بار 1<br>TWICE PER DAY.......................................دن میں دو بار 2<br>THREE TIMES PER DAY.........................دن میں تین بار 3<br>FOUR TIMES PER DAY...........................دن میں 4 بار 4<br>FIVE TIMES PER DAY.............................دن میں 5 بار 5<br>OCCASIONALLY کبھی کبھی 6<br>NEVER کبھی نہیں 77<br>REFUSED..انکار 99	
306	What is your tribe or ethnic group?<br>آپ کا قبیلہ/قومیت کیا ہے؟	1 URDU SPEAKING اردو اسپیکنگ 1<br>2 SINDHI سندھی 2<br>3 PUSHTON پشتون 3<br>4 BALUCHI بلوچی 4<br>5 BARAHVI براہوی 5<br>6 SARAIKI سرائیکی 6<br>7 HINDKO ہندکو 7<br>8 PUNJABI پنجابی 8<br>96 OTHER دیگر (وضاحت کریں) 96<br>99 REFUSED انکار کردیا 99	

4. REPRODUCTION

NO.	QUESTIONS AND FILTERS	CODING CATEGORIES	SKIP
401	Now I will read you a list of statements, and I would like for you to tell me how many of the statements are true for you. You should not tell me which statements are true, only how many. By giving only the number of statements that are true for you, this will preserve your privacy as I will not know which statements are true for you. After I read the list, please tell me how many of these statements are true for you. First, we will complete an example:<br><br>اب میں کچھ سوالات کی فہرست پڑھوں گی اور آپ سے یہ گزارش ہے کہ آپ مجھے بتائیں کہ ان میں سے کتنی باتیں آپ کے بارے میں صحیح ہیں .آپ کو مجھے یہ نہیں بتانا چاہیئے کہ ان میں سے کون سی بات صحیح ہے، بلکہ صرف یہ بتانا ہے کہ ان میں سے کتنی باتیں صحیح ہیں۔ صرف ان کی تعداد بتانی ہے کہ آپ کے بارے میں ان میں سے کتنی باتیں صحیح ہیں۔ ان میں سے کل کتنے سوالوں کے جوابات ہاں میں ہیں آپ کے جوابات کی مکمل رازداری رکھی جائے گی اور مجھے یہ نہیں معلوم ہو گاکہ کون سا جواب ہاں میں یا نہ میں تھا ۔ اس کہ بعد میں لسٹ پڑھوں گی اور آپ نوٹ کریں گی کہ کس سوال کا جواب ہاں میں ہے پہلے ہم کچھ مثالوں کے زریعے سمجھ لیتے ہیں کہ کیسے کرنا ہے مثلاً<br><br>1. I have three children. — 1 میرے 3 بچے ہیں ۔<br>2. I am 22 years old. — 2 میری عمر 22 سال ہے۔<br>3. I have received antenatal care during a pregnancy. — 3 میں نے دوران۔ حمل معائنہ /چیک اپ کروایا ہے<br>4. I have had a tetanus toxoid vaccination. — 4 میں نے تشنج سے بچاوکا حفاظتی ٹیکہ لگوایا ہے۔<br><br>How many of these statements are true for you?<br>آپ کے بارے میں اوپر بیان کئے گئے جوابات میں سے کتنے صحیح ہیں ؟	NUMBER OF STATEMENTS بیان کی تعداد [ ]<br><br>REFUSED انکار کردیا 99	
402	Do you have any questions about how to answer these questions?<br>کیا اس سلسلے میں آپ کوئی سوال کرنا چاہیں گی ؟	1 YES ہاں 1<br>2 NO نہیں 2	1 → ANSWER QUESTIONS
403	Now we will complete the first real list of statements about your health. I will read you the list of statements, and I would like for you to tell me how many of these statements are true for you.<br><br>اب میں اپنے اصل بیان جو کہ صحت کے بارے میں ہے کچھ سوال کرونگی ۔ میں آپ کو بیانات کی فہرست پڑھ دونگی اور میں آپ سے پوچھنا چاہونگی کہ ان میں سے کتنے بیان آپ کے لئے درست ہیں.<br><br>1. I have heard of an illness called (Polio)<br>1 میں نے پولیو کی بیماری کے بارے میں سنا ہے<br>2. I have received a medical injection in the past 2 years<br>2 مجھے گزشتہ 2 سالوں میں طبی انجکشن لگا ہے<br>3. I have ever smoked cigarettes<br>3 میں نے کبھی سگریٹ نوشی کی ہے<br>4. I had malaria as a child<br>4 مجھے بچپن میں ملیریا ہوچکا ہے<br>5. I have had an induced abortion (ended a pregnancy on purpose)<br>5 میں نے کسی وجہ سے حمل ضائع کروایا(حمل کو کسی خاص وجہ سے ختم کرواناInduced Abortion)<br>How many of these statements are true for you?<br>ان سوال میں سے کتنے سوالوں کے جوابات ہاں میں ہیں ؟	NUMBER OF STATEMENTS بیان کی تعداد [ ]<br><br>99 REFUSED انکار کردیا 99	

4. REPRODUCTION

NO.	QUESTIONS AND FILTERS	CODING CATEGORIES	SKIP
404	Here is the next list of statements about your health. Please tell me how many of these statements are true for you.<br>اب میرے پاس آپ کی صحت سے متعلق سوالات کی ایک اور فہرست ہے ۔ برائے مہربانی مجھے بتائیے گا کہ آپ کے بارے میں ان میں سے کتنے جوابات صحیح ہیں ؟<br>1. I have had a cold in the last year<br>1 گزشتہ سال مجھے زکام ہوا تھا.<br>2. I have heard of an illness called diabetes (high blood sugar)<br>2 میں نے ایک بیماری جس کا نام ذیابیطس ہے(ہائی بلڈشوگر) کے بارے میں سنا ہے<br>3. I know someone who has told me they have High Blood Pressure<br>3 میں کسی کو جانتی ہوں جس نے مجھے بتایا ہے کہ اسے ہائی بلڈ پریشر ہے<br>4. I have been diagnosed with cancer<br>4 مجھے کینسر کی بیماری تشخیص ہوئی ہے۔<br>5. I have had an induced abortion (ended a pregnancy on purpose)<br>5 میں نے حمل کو اپنی مرضی سےضائع کروایا ہے۔(حمل کو کسی مقصد کی وجہ سے ختم کروانا)<br>How many of these statements are true for you?<br>ان سوالوں میں سے کتنوں کے جوابات ہاں میں ہیں ؟	NUMBER OF STATEMENTS بیان کی تعداد [ ]<br>99 REFUSED انکار کردیا 99	
	Now I would like to ask some questions about marriage and sexual activity in order to gain a better understanding of some important life issues.<br>اب میں آپ سے شادی اور ازدواجی ملاپ کے بارے میں کچھ سوالات پوچھنا چاہونگی تا کہ آپ کی زندگی کے کچھ اہم معاملات کو بہتر طریقہ سے سمجھ سکوں۔		
405	How old were you when you first got married?<br>پہلی شادی کے وقت آپ کی عمر کتنی تھی؟	AGE IN COMPLETED YEARS عمر مکمل سالوں میں [ ][ ]<br>88 DON'T KNOW معلوم نہیں 88<br>99 REFUSED انکار کردیا 99	
406	How old were you when you had sexual intercourse for the very first time?<br>کس عمر میں آپ نے پہلی دفعہ جنسی ملاپ کیا تھا؟	NEVER HAD SEXUAL INTERCOURSE کبھی جنسی ملاپ نہیں کیا 0<br>AGE IN YEARS عمر سالوں میں [ ][ ]<br>88 DON'T KNOW معلوم نہیں 88<br>99 REFUSED انکار کردیا 99	0 → END OF SURVEY
407	When was the last time you had sexual intercourse?<br>(RECORD IN UNITS RESPONDENT USES) *If less than 12 months ago, answer must be recorded in months, weeks, or days. Enter 0 days for today.*<br>آخری بار آپ نے کب جنسی ملاپ کیا تھا ؟<br>(جواب دہندہ جس یونٹ کو بتائے وہ ریکارڈ کریں)<br>اگر مدت12 ماہ سے کم ہو تو ضروری ہے کہ جواب کو مہینوں ، ہفتوں یا دنوں میں ریکارڈ کریں اور اگر آج ہی کیا ہو تو دن 00لکھیں۔	[ ][ ] Days ago اتنےدن پہلے<br>[ ][ ] Weeks ago اتنےہفتوں پہلے<br>[ ][ ] Months ago اتنے مہینوں پہلے<br>[ ][ ] Years ago اتنے سالوں پہلے<br>88 DON'T KNOW معلوم نہیں 88<br>99 REFUSED انکار کردیا 99	
408	Now I would like to ask about all the Live births you have had during your life. Have you ever given birth?<br>اب میں آپ سے پیدا ہونے والے زندہ بچوں کی پیدائش کے بارے میں پوچھنا چاہونگی۔ کیا کبھی آپ بچے کی پیدائش کے مرحلہ سے گزری ہیں؟	1 YES ہاں 1<br>2 NO نہیں 2<br>99 REFUSED انکار کردیا 99<br>88 DON'T KNOW معلوم نہیں 88	2 → 420
409	How old were you when you gave birth for the first time?<br>جب پہلی با راپ کے بچہ کی پیدائش ہوئی تواس وقت آپ کی عمر کیا تھی ؟	AGE IN YEARS عمر سالوں میں [ ][ ]<br>88 DON'T KNOW معلوم نہیں 88<br>99 REFUSED انکار کردیا 99	
410	Do you have any sons or daughters to whom you have given birth who are now living with you?<br>آپ کے کوئی بیٹے یا بیٹیاں ایسی ہیں جن کی پیدائش آپ سے ہوئی ہو اور اس وقت وہ آپکے ساتھ رہتے ہوں؟	1 YES ہاں 1<br>2 NO نہیں 2<br>99 REFUSED انکار کردیا 99	2 → 412

4. REPRODUCTION

NO.	QUESTIONS AND FILTERS	CODING CATEGORIES	SKIP
411	How many sons live with you?<br>ماشاالله کتنے بیٹے آپ کے ساتھ رہتے ہیں؟<br>And how many daughters live with you?<br>اورماشاالله کتنی بیٹیاں آپ کےساتھ رہتی ہیں؟<br>IF NONE, RECORD '00'.<br>اگر کوئی نہیں تو 00 درج کریں<br>IF REFUSED, RECORD '99'<br>اگر انکار کردیاہے تو 99 ریکارڈ کریں	SONS AT HOME گھر میں رہنے والے بیٹوں کی تعداد<br>DAUGHTERS AT HOME گھر میں رہنے والی بیٹیوں کی تعداد	
412	Do you have any sons or daughters to whom you have given birth who are alive but do not live with you?<br>کیا آپ کے کوئی ایسے بیٹے اور بیٹیاں ہیں جنکی پیدائش آپ سے ہوئی ہو اور وہ زندہ ہوں لیکن وہ آپ کے ساتھ رہتےنہیں ہوں؟	1 YES ہاں 1<br>2 NO نہیں 2<br>99 REFUSED انکار کردیا 99	414
413	How many sons are alive but do not live with you?<br>آپ کے ایسے کتنےبیٹے ہیں جو حیات ہیں لیکن آپ کے ساتھ نہیں رہتے ہیں؟<br>And how many daughters are alive but do not live with you?<br>ماشاالله آپ کی ایسی کتنےبیٹیاں ہیں جو حیات ہیں لیکن آپ کے ساتھ نہیں رہتی ؟<br>IF NONE, RECORD '00'. اگر کوئی نہیں تو 00 درج کریں<br>IF REFUSED, RECORD '99' اگر انکار کردیاہے تو 99 ریکارڈ کریں	SONS ELSEWHERE<br>بیٹے جن کی رہائش کہیں اور ہے<br>DAUGHTERS ELSEWHERE<br>بیٹیاں جن کی رہائش کہیں اور ہے	
414	Have you ever given birth to a boy or girl who was born alive but later died?<br>کیا آپ کے اپنے کسی ایسے بیٹے یا بیٹی کی پیدائش ہوئی تھی جو پیدائش کے وقت تو زندہ تھا /تھی مگر بعد میں اس کا انتقال ہو گیا ہو ؟<br>IF NO, PROBE: Any baby who cried or showed signs of life but did not survive?<br>اگر نہیں، تو پوچھیں کوئی بھی ایسا بچہ جو رویا ہو یا جس میں زندگی کی علامات موجود ہوں ، لیکن اس کا انتقال ہو گیا ہو ؟	1 YES ہاں 1<br>2 NO نہیں 2<br>99 REFUSED انکار کردیا 99	416
415	How many boys have died?<br>کتنے بیٹوں کا انتقال ہو چکا ہے؟<br>And how many girls have died?<br>کتنی بیٹیوں کا انتقال ہو چکا ہے؟	BOYS DEAD مرنے والے بیٹوں کی تعداد<br>GIRLS DEAD مرنے والی بیٹیوں کی تعداد	

4. REPRODUCTION

NO.	QUESTIONS AND FILTERS	CODING CATEGORIES	SKIP
416	SUM ANSWERS TO 411, 413, AND 415, AND ENTER TOTAL.<br>سوال نمبر 413،411 اور 415، کا حاصل جمع لکھیں<br>IF NONE, RECORD '00'.<br>اگر کوئی نہیں تو 00 درج کریں<br>IF REFUSED, RECORD '99'<br>اگر انکار کردیاہے تو 99 ریکارڈ کریں	TOTAL BIRTHS زندہ اور مردہ بچوں کی مجموعی تعداد [ ][ ]	
417	CHECK 416: سوال نمبر 416 کو چیک کریں:<br>Just to make sure that I have this right: you have had in TOTAL _____ births during your life. Is that correct?<br>کیاآپ مجھے اس بات کی یقین دہانی کر اسکتی ہیں کہ یہ درست ہے کہ آپ کی زندگی میں ------------ بچوں کی پیدائش ہوئی ہے؟<br>YES ہاں ☐ ↓	NO نہیں ☐ →	411
418	CHECK 416: سوال نمبر 416 کو چیک کریں:<br>ONE OR MORE BIRTHS<br>ایک یا ایک سے زیادہ بچوں کی پیدائش ☐ ↓	NO BIRTHS ☐ →<br>کوئی پیدائش نہیں ہوئی	420
419	C<br>FOR EACH BIRTH SINCE JANUARY 2015 **(Column 1)**, ENTER 'B' IN THE MONTH OF BIRTH IN THE CALENDAR. WRITE THE NAME OF THE CHILD TO THE LEFT OF THE 'B' CODE. FOR EACH BIRTH,ASK THE NUMBER OF MONTHS THE PREGNANCY LASTED AND RECORD 'P' IN EACH OF THE PRECEDING MONTHS ACCORDING TO THE DURATION OF PREGNANCY. (NOTE: THE NUMBER OF 'P's MUST BE ONE LESS THAN THE NUMBER OF MONTHS THAT THE PREGNANCY LASTED.)<br>جنوری 2015 سے لے کر اب تک ہونے والی ہر پیدائش (کالم 1)،کیلنڈر میں پیدائش کے مہینہ کے خانہ میں (B) لکھیں۔B کوڈ کے الٹے ہاتھ والے خانہ میں بچے کانام لکھیں۔<br>ہر پیدائش کے لیے حمل کےآخری مہینہ تک کی تعداد ،ماہواری کی آخری تاریخ پوچھیں اور حمل کی مدت کے مطابق ہر گزشتہ مہینے میں P درج کریں۔(نوٹ،P کی تعداد لازمی طورپر زچگی کہ مہینوں کی تعداد سے ایک نمبر کم ہوگی۔		
420	Now I would like to ask you some questions about pregnancy.<br>Are you pregnant now?<br>اب میں آپ سے حمل سے متعلق کچھ سوالات پوچھنا چاہتی ہوں. کیا آپ اس وقت حمل سے ہیں؟	1 YES ہاں 1<br>2 NO نہیں 2<br>88 DON'T KNOW / UNSURE معلوم نہیں/غیر یقینی 88<br>99 REFUSED انکار کردیا 99	If 418=1, go to 424.<br>If 418=0, go to 425.
421	How many months pregnant are you?<br>RECORD NUMBER OF COMPLETED MONTHS.<br>اس وقت آپ کے حمل کا کونسا مہینہ چل رہا ہے(مکمل مہینوں کی تعداد لکھیں)<br>C<br>ENTER 'P's IN THE CALENDAR, BEGINNING WITH THE MONTH OF INTERVIEW AND FOR THE TOTAL NUMBER OF COMPLETED MONTHS.<br>کیلینڈر میں P درج کریں، انٹرویو کے مہینے کے ساتھ شروعات کریں اور مکمل مہینوں کی مجموعی تعداد لکھیں۔	MONTHS مہینہ [ ][ ]<br>88 DON'T KNOW معلوم نہیں 88<br>99 REFUSED انکار کردیا 99	
422	When you got pregnant, did you want to get pregnant at that time?<br>جس وقت آپ حاملہ ہوگئی تھیں،تو کیا اس وقت آپ حاملہ ہونا چاہتی تھیں؟	1 YES ہاں 1<br>2 NO نہیں 2<br>88 DON'T KNOW / UNSURE معلوم نہیں/غیر یقینی 88<br>99 REFUSED انکار کردیا 99	→ 425 (1)

4. REPRODUCTION

NO.	QUESTIONS AND FILTERS	CODING CATEGORIES	SKIP
423	Did you want to have a baby later on or did you not want any more children?<br>کیا آپ بچہ کی پیدائش کچھ دنوں بعد چاہتی تھیں یا آپ اس سے زیادہ مزید کوئی اور بچہ نہ	1 LATER — بعد میں 1<br>2 NO MORE/NONE — اور نہیں چاہتی/نہیں 2<br>88 DON'T KNOW / UNSURE — معلوم نہیں/غیر یقینی 88<br>99 REFUSED — انکار کردیا 99	→ 425
424	Have you given birth within the last six months?<br>کیا آپ نے گزشتہ 6 ماہ کے دوران کسی بچے کو جنم دیا ہے؟	1 YES — ہاں 1<br>2 NO — ہیں 2<br>99 REFUSED — انکار کردیا 99	
425	Have you ever had a pregnancy that miscarned, was aborted, or ended in a stillbirth?<br>کیا کبھی اپ کا کوئی حمل جو ضائع ہو گیا ہو ،یا حمل ضائع کروایا گیا ہو یا حمل مردہ بچے کی ولادت پر ختم ہو ا ہو؟	1 YES — ہاں 1<br>2 NO — ہیں 2<br>99 REFUSED — انکار کردیا 99	→ 501
426	When did the last such pregnancy end?<br>گزشتہ ایسے کسی بھی حمل کا اختتام کس مہینے ،سال میں ہوا؟<br>(RECORD IN UNIT RESPONDENT USES)<br>جواب دہندہ کے بتائے ہوئے یونٹ کے مطابق ریکارڈ کریں	DAY AGO — دنوں پہلے [ ][ ]<br>WEEKS AGO — ہفتوں پہلے [ ][ ]<br>MONTHS AGO — مہینوں پہلے [ ][ ]<br>YEAR AGO — سالوں پہلے [ ][ ]<br>88 DON'T KNOW — 88 معلوم نہیں88 معلوم نہیں<br>99 REFUSED — 99 انکار کر دیا	
427	How many pregnancies were miscarried, aborted, or ended in a stillbirth?<br>مجموعی طور پر کل کتنے حمل خود بخود ضائع ہوئے ، حمل کو کسی وجہ سے ضائع کروایا گیا یا حمل کا اختتام مردہ بچے کی ولادت پرہوا ہو؟	NUMBER OF PREGNANCY LOSSES — حمل ضائع ہونے کی تعداد [ ][ ]<br>99 REFUSED — انکار کردیا 99	
428	How many pregnancies ended in a stillbirth?<br>کتنے مردہ بچوں کی پیدائش ہوئی؟	NUMBER OF STILLBIRTHS — مردہ بچوں کی تعداد [ ][ ]<br>00 NONE — کوئی نہیں 00<br>99 REFUSED — انکار کردیا 99<br>88 DON'T KNOW / UNSURE — معلوم نہیں/غیر یقینی 88	
429	How many pregnancies were miscarried?<br>ی بار حمل خود بخود ضائع ہو	NUMBER OF MISCARRIAGES — اسقاط حمل کی تعداد [ ][ ]<br>00 NONE — کوئی نہیں 00<br>99 REFUSED — انکار کردیا 99	
430	How many pregnancies were terminated by induced abortion?<br>کتنی بار حمل کو اپنی مرضی سےخارج کروایا ؟	NUMBER OF ABORTIONS — حمل ضائع کروانے کی تعداد [ ][ ]<br>00 NONE — کوئی نہیں 00<br>99 REFUSED — انکار کردیا 99	00 → 444
431	When was the last induced abortion?<br>آخری حمل کب ضائع کروایا گیا ؟<br>(RECORD IN UNIT RESPONDENT USES)<br>جواب دہندہ کے بتائے ہوئے یونٹ ریکارڈ کریں	[ ][ ] Days ago — دنوں کی تعداد<br>[ ][ ] Weeks ago — ہفتوں کی تعداد<br>[ ][ ] Months ago — مہینوں کی تعداد<br>[ ][ ] Years ago — سالوں کی تعداد<br>DON'T KNOW 88 — معلوم نہیں<br>REFUSED 99 — انکار کردیا	

4. REPRODUCTION

NO.	QUESTIONS AND FILTERS	CODING CATEGORIES	SKIP
432	From whom/where did you receive induced abortion services the last time?<br><br>آخری بار کس سے/کہاں سے اپنی مرضی سے حمل ضائع کروانے کی سہولت/خدمات حاصل کیں؟<br>SELECT ALL THAT APPLY<br>(تمام پر نشان لگائیں جو لاگو ہوتے ہیں)	PUBLIC SECTOR پبلک /سرکاری سیکٹر<br>11 GOVT. HOSPITAL/RHSC سرکاری ہسپتال 11<br>12 RURAL HEALTH CENTER, MCH دیہی ہیلتھ سینٹر 12<br>13 MCH.. ایم سی ایچ 13<br>14 FAMILY HEALTH CENTRE/ FWW فیملی ہیلتھ سیکٹر 14<br>15 MOBILE SERVICE CAMP موبائل سروس کیمپ 15<br>16 LADY HEALTH WORKER لیڈی ہیلتھ ورکر 16<br>17 LH VISITOR لیڈی ہیلتھ وزیٹر 17<br>18 BASIC HEALTH UNIT بنیادی ہیلتھ یونٹ 18<br>19 MALE MOBILIZER مرد موبائلیزر 19<br>20 FWA ایف-ڈبلو-اے 20<br>21 OTHER PUBLIC دیگر سرکاری وضاحت کریں 21<br>PRIVATE/NGO MEDICAL SECTOR پرائیوٹ میڈیکل سیکٹر<br>22 PRIVATE/NGO/HOSPITAL/CLINIC پرائیوٹ /ہسپتال /کلینک 22<br>23 PHARMACY, CHEMISTS فارمیسی /کیمسٹ 23<br>24 PRIVATE DOCTOR پرائیوٹ ڈاکٹر 24<br>25 HOMEOPATH ہومیوپیتھ 25<br>26 DISPENSOR/ COMPOUNDER ڈسپنسر /کمپاؤنڈر 26<br>28 MOBILE SERVICE CAMP موبائل سروس کیمپ 28<br>27 OTHER PRIVATE MEDICAL دیگر پرائیوٹ میڈیکل 27<br>OTHER SOURCE دیگر ذرائع<br>31 SHOP (NOT PHARMACY/CHEMIST) دکان /فارمیسی /کیمیسٹ 31<br>32 FRIEND/RELATIVE دوست /رشتہ دار 32<br>33 HAKIM حکیم 33<br>34 DAI,TRAD. BIRTH ATTENDANT دائی یا پرانے طریقوں سے ولادت کروانے والی 34<br>96 OTHER (SPECIFY) دیگر وضاحت کریں 96<br>88 DON'T KNOW معلوم نہیں 88<br>99 REFUSED انکار کردیا 99	
433	From whom did you learn about this person/place abortion services (primary source of information)?<br><br>کس سے سن کر آپ نے اس جگہ /شخص کا انتخاب کیا؟	1 NEIGHBOR پڑوسی 1<br>2 FRIEND دوست 2<br>3 MOTHER ماں 3<br>4 MOTHER-IN-LAW ساس 4<br>5 HUSBAND شوہر 5<br>6 OTHER RELATIVE دوسرے رشتہ دار 6<br>7 DOCTOR /PHYSICIAN ڈاکٹر / فزیشن 7<br>8 NURSE/MIDWIFE/LHV WORKING AT HEALTH FACILITY<br>نرس/مڈوائف/LHV/مرکز صحت پہ کام کرنے والے 8<br>9 NURSE/MIDWIFE MAKING A HOME VISIT<br>نرس/مڈوائف/LHV/جس نے گھر پر دورہ کیاہو 9<br>10 LHW MAKING A HOME VISIT<br>ایل ایچ ڈبلیو جنہوں نے گھر پر دورہ کیاہو 10<br>11 PHARMACIST فارمیسی والے سے 11<br>12 NO ONE کوئی نہیں 12<br>96 OTHER (SPECIFY) ____________________ دیگر وضاحت کریں 96<br>88 DON'T KNOW معلوم نہیں 88<br>99 REFUSED انکار کردیا 99	
434	Who influenced you the most when deciding on this abortion?<br><br>اپنی مرضی سے اس حمل کو ضائع کروانے کے فیصلے پر کون سب سے زیادہ اثر ا	1 NEIGHBOR پڑوسی 1<br>2 FRIEND دوست 2<br>3 MOTHER ماں 3<br>4 MOTHER-IN-LAW ساس 4<br>5 HUSBAND شوہر 5<br>6 OTHER RELATIVE دوسرے رشتہ دار 6<br>7 DOCTOR /PHYSICIAN ڈاکٹر / فزیشن 7<br>8 NURSE/MIDWIFE/LHV WORKING AT HEALTH FACILITY<br>نرس/مڈوائف/LHV/مرکز صحت پہ کام کرنے والے 8<br>9 NURSE/MIDWIFE MAKING A HOME VISIT<br>نرس/مڈوائف/LHV/جس نے گھر پر دورہ کیاہو 9<br>10 LHW MAKING A HOME VISIT<br>ایل ایچ ڈبلیو جنہوں نے گھر پر دورہ کیاہو 10<br>11 PHARMACIST فارمیسی والے سے 11<br>12 NO ONE کوئی نہیں 12<br>96 OTHER (SPECIFY) ____________________ دیگر وضاحت کریں 96<br>88 DON'T KNOW معلوم نہیں 88<br>99 REFUSED انکار کردیا 99	

4. REPRODUCTION

NO.	QUESTIONS AND FILTERS	CODING CATEGORIES	SKIP
435	What was done to have the pregnancy terminated?<br>کس طریقہ سے آپ کا حمل ضائع کیا گیا تھا؟	1 SURGICALLY سرجری 1<br>2 INJECTION انجکشن 2<br>3 TOOK MEDICINES/MEDICATION ABORTION (MA)<br>دوائیں کھائیں/حمل ضائع کرنے والی دوائیں کھائیں(ایم اے) 3<br>4 INSERTED HERBS OR OBJECT IN WOMB, SPECIFY<br>بچہ دانی میں جڑی بوٹی رکھی یا کو ئی چیز ،وضاحت کریں 4<br>5 TOOK HOMEMADE CONCOCTION, SPECIFY<br>گھر میں بنی اجزاء ملا کر کوئی دوائی ، وضاحت کریں 5<br>96 OTHER (SPECIFY) دیگر وضاحت کریں 96<br>99 REFUSED انکار کردیا 99	1, 2 → 439<br>3 → 437<br>4, 5, 96, 99 → 439
436	What was the name of the surgical procedure used to end the pregnancy?<br>حمل ختم کرنے کے لیے جو سرجری کا طریقہ استعمال کیا اسے کیا کہتے ہیں ؟	1 VACUUM ASPIRATION (MVA or EVA) ،Vacuum<br>ویکیوم سے کھینچ کر باہر نکالا(MVA/EVA) 1<br>2 DILATION AND CURETTAGE D&C/ D&E رحم کے اندر سے صفائی 2<br>SHARP CURETTAGE<br>3 IUD INSERTION آئی یو ڈی (چھلہ)کو رحم کے اند رکھنا 3<br>96 OTHER, SPECIFY دیگر وضاحت کریں 96<br>88 DON'T KNOW معلوم نہیں 88<br>99 REFUSED انکار کردیا 99	→439
437	What kind of medication did you use to end the pregnancy?<br>آپ نے حمل ختم کرنے کے لیے کون سی دوائیاں استعمال کیں؟	2 MISOPROSTOL ALONE (ST MOM) میسوپروسٹول 2<br>3 ORAL CONTRACEPTIVE PILLS منہ سے کھانے والی مانع حمل ادویات 3<br>96 OTHER, SPECIFY دیگر وضاحت کریں 96<br>88 DON'T KNOW معلوم نہیں 88<br>99 REFUSED انکار کردیا 99	96, 88, 99 → 439
438	In total, how many tablets or pills did you take to end the pregnancy?<br>مجموعی طور پر ،آپ نے حمل کو ختم کرنے کے لیے کتنی گولیاں استعمال کی تھیں؟	NUMBER OF PILLS گولیوں کی تعداد [ ][ ]<br>88 DON'T KNOW معلوم نہیں 88<br>99 REFUSED انکار کردیا 99	
439	After termination, did you experience any health problems?<br>(SELECT ALL THAT APPLY)<br>حمل کے ختم ہونے کے بعد صحت سے متعلق آپ کو کونسی مشکلات پیش آئیں	1 HEAVY BLEEDING خون کا زیادہ اخراج 1<br>2 VOMITING/NAUSEA الٹیاں /جی متلانا 2<br>3 ABDOMINAL PAIN پیٹ کا درد 3<br>4 FEVER بخار 4<br>5 FOUL-SMELLING DISCHARGE/INFECTION<br>اندام نہانی سے بدبو دار پانی کا اخراج/انفیکشن 5<br>6 NO HEALTH PROBLEM صحت کا کوئی مسئلہ نہیں 6<br>96 OTHER, SPECIFY دیگر وضاحت کریں 96<br>99 REFUSED انکار کردیا 99	6 → End Section
440	Did you seek care or treatment for any of these problems?<br>صحت کے ان مسائل کے لیے آپ نے کوئی علاج کروایا	1 YES ہاں 1<br>2 NO نہیں 2<br>99 REFUSED انکار کردیا 99	2, 99 → End Section

4. REPRODUCTION

NO.	QUESTIONS AND FILTERS	CODING CATEGORIES	SKIP
441	From whom or where did you receive treatment for your symptoms?<br>علامات ظاہر ہونے کی صورت میں آپ نےکس سے اور کہاں سے علاج کروایا؟<br>SELECT ALL THAT APPLY<br>(تمام پر نشان لگائیں جو لاگو ہوتے ہیں)	PUBLIC SECTOR پبلک /سرکاری سیکٹر<br>11 GOVT. HOSPITAL/RHSC سرکاری ہسپتال 11<br>12 RURAL HEALTH CENTER, MCH دیہی ہیلتھ سینٹر 12<br>13 MCH.. ایم سی ایچ 13<br>14 FAMILY HEALTH CENTRE/ FWW فیملی ہیلتھ سیکٹر 14<br>15 MOBILE SERVICE CAMP موبائل سروس کیمپ 15<br>16 LADY HEALTH WORKER لیڈی ہیلتھ ورکر 16<br>17 LH VISITOR لیڈی ہیلتھ وزیٹر 17<br>18 BASIC HEALTH UNIT بنیادی ہیلتھ یونٹ 18<br>19 MALE MOBILIZER مرد موبائلیزر 19<br>20 FWA ایف-ڈبلو-اے 20<br>21 OTHER PUBLIC دیگر سرکاری وضاحت کریں 21<br>PRIVATE/NGO MEDICAL SECTOR پرائیوٹ میڈیکل سیکٹر<br>22 PRIVATE/NGO/HOSPITAL/CLINIC پرائیوٹ /ہسپتال /کلینک 22<br>23 PHARMACY, CHEMISTS فارمسی /کیمسٹ 23<br>24 PRIVATE DOCTOR پرائیوٹ ڈاکٹر 24<br>25 HOMEOPATH ہومیوپیتھ 25<br>26 DISPENSOR/ COMPOUNCER ڈسپنزر /کمپاوڈر 26<br>28 MOBILE SERVICE CAMP موبائل سروس کیمپ 28<br>27 OTHER PRIVATE MEDICAL دیگر پرائیوٹ میڈیکل 27<br>**OTHER SOURCE** دیگر ذرائع<br>31 SHOP (NOT PHARMACY/CHEMIST) دکان /فارمیسی /کیمیسٹ 31<br>32 FRIEND/RELATIVE دوست /رشتہ دار 32<br>33 HAKIM حکیم 33<br>34 DAI,TRAD. BIRTH ATTENDANT دائی یا پرانے طریقوں سے ولادت کروانے والی 34<br>96 OTHER (SPECIFY) دیگر وضاحت کریں 96<br>____________________<br>88 DON'T KNOW معلوم نہیں 88<br>99 REFUSED انکار کردیا 99	
442	Who influenced you the most when deciding on this place?<br>کس کی رائے سے اثر انداز ہو کر آپ نے اس جگہ کا انتخاب کیا ؟	1 NEIGHBOR پڑوسی 1<br>2 FRIEND دوست 2<br>3 MOTHER ماں 3<br>4 MOTHER-IN-LAW ساس 4<br>5 HUSBAND شوہر 5<br>6 OTHER RELATIVE دوسرے رشتہ دار 6<br>7 DOCTOR /PHYSICIAN ڈاکٹر / فزیشن 7<br>8 NURSE/MIDWIFE/LHV WORKING AT HEALTH FACILITY<br>نرس/مڈوائف/LHVمرکز صحت پہ کام کرنے والے 8<br>9 NURSE/MIDWIFE MAKING A HOME VISIT<br>نرس/مڈوائف/LHVجس نے گھر پر دورہ کیاہو 9<br>10 LHW MAKING A HOME VISIT<br>ایل ایچ دبلیو جنہوں نے گھر پر دورہ کیاہو 10<br>11 PHARMACIST فارمیسی والے سے 11<br>12 NO ONE کوئی نہیں 12<br>96 OTHER (SPECIFY) ____________________دیگر وضاحت کریں 96<br>88 DON'T KNOW معلوم نہیں 88<br>99 REFUSED انکار کردیا 99	

<table>
<tr><th colspan="4">4. REPRODUCTION</th></tr>
<tr><th>NO.</th><th>QUESTIONS AND FILTERS</th><th>CODING CATEGORIES</th><th>SKIP</th></tr>
<tr><td>443</td><td>Did you receive any family planning counseling during this visit?

کیا آپ نے اس دورے کے دوران خاندانی منصوبہ بندی کے حوالہ سے کوئی مشاورت حاصل کی؟</td><td>1 YES — ہاں 1
2 NO — نہیں 2
88 DON'T KNOW — معلوم نہیں 88
99 REFUSED — انکار کردیا 99</td><td></td></tr>
<tr><td>444</td><td colspan="2">C
FOR EACH PREGNANCY THAT DID NOT END IN A LIVE BIRTH IN JULY 2015 (Col. 1) OR LATER, ENTER 'T' IN THE CALENDAR IN THE MONTH THAT THE PREGNANCY TERMINATED AND 'P' FOR THE REMAINING NUMBER OF COMPLETED MONTHS OF PREGNANCY.

IF THERE ARE MORE THAN FOUR PREGNANCIES THAT DID NOT END IN A LIVE BIRTH, USE AN ADDITIONAL QUESTIONNAIRE STARTING ON THE SECOND LINE.
ہر وہ زچگی جسمیں زندہ بچے کی ولادت جولائی 2015
کالم 1یا اس کے بعد ہوئی ہو، یا جس مہینے زچگی ضائع ہوگئی ہو تو T پر نشان کریں۔ P پر نشان کریں۔اور جنتے مہینے مکمل حمل ٹھرا رہا اسے
اگر 4 سے زیادہ بار زچگی ہوئی ہے اور زندہ بچے کی ولادت نہیں ہوئی ،اس کے لیے ایک علیحدہ سوالنامہ پُر کریں اور اس کی دوسری لائن سے شروع کریں۔
نوٹ: 1- انٹرویو لینے والے مہینے سے شروع کرتے ہوئے جولائی 2015 تک کی معلومات حاصل کریں۔</td><td></td></tr>
<tr><td colspan="4">(1) Year of fieldwork is assumed to be 2017. For fieldwork beginning in 2018 or 2019, the year should be adjusted.</td></tr>
</table>

5. FAMILY PLANNING

5. خاندانی منصوبہ بندی

NO.	QUESTIONS AND FILTERS	CODING CATEGORIES	SKIP
501	Now I would like to talk about family planning - the various ways or methods that a man and woman can use to delay or avoid a pregnancy. Have you heard of:<br><br>اب میں آپ سے خاندانی منصوبہ بندی کے مختلف طریقوں کے بارے میں بات کرنا چاہتی ہوں۔ جو مرد یا عورت حمل میں تاخیر یا حمل سےبچنے کے لیے استعمال کرسکتے ہیں۔<br>کیا آپ نے ان کے بارے میں سنا ہے:		
1	Female Sterilization. PROBE: Women can have an operation to avoid having any more children.<br><br>خاتون کی نل بندی: مزید بچوں کی پیدائش روکنے کے لئے خاتون کا آپریشن ہو سکتا ہے۔	YES ……………… ……… …. …. ……. ………… ہاں 1<br>NO ……………… ……… …. …. ……. ………… نہیں 2<br>REFUSED ……… ……… …. …. ……. ……انکار کردیہ 99	
2	Male Sterilization. PROBE: Men can have an operation to avoid having any more children.<br><br>مرد کی نل بندی: مزید بچوں کی پیدائش روکنے کے لیے مرد کا آپریشن ہوسکتا ہے ؟	YES ……………… ……… …. …. ……. ………… ہاں 1<br>NO ……………… ……… …. …. ……. ………… نہیں 2<br>REFUSED ……… ……… …. …. ……. ……انکار کردیہ 99	
3	IUD. PROBE: Women can have a loop or coil placed inside them by a doctor or a nurse<br><br>چھلہ: عورتوں کو ڈاکٹر یا نرس کی طرف سے ان کے اندر رکھی ہونی لوپ یا کوائل ہو سکتی ہے.	YES ……………… ……… …. …. ……. ………… ہاں 1<br>NO ……………… ……… …. …. ……. ………… نہیں 2<br>REFUSED ……… ……… …. …. ……. ……انکار کردیہ 99	
4	Injectables. PROBE: Women can have an injection by a health provider that stops them from becoming pregnant for one or more months.<br><br>ٹیکہ:خواتین کو صحت کی سہولیات فراہم کرنے والے کی طرف سے ایک ٹیکہ لگایا جاسکتا ہے جو انہیں ایک یا ایک سے زیادہ مہینے کے لیے حاملہ ہونے سے روکتا ہے.	YES ……………… ……… …. …. ……. ………… ہاں 1<br>NO ……………… ……… …. …. ……. ………… نہیں 2<br>REFUSED ……… ……… …. …. ……. ……انکار کردیہ 99	
5	Implants. PROBE: Women can have one or more small rods placed in their upper arm by a doctor or nurse which can preventy pregnancy for one or more years.<br><br>ایملانٹ: خواتین ایک یا ایک سے زیادہ چھوٹے سائز کی پلاسٹک راڈز ڈاکٹر یا نرس کی مدد سے بازو کی جلد کے نیچے رکھ سکتی ہے جو کہ ایک یا ایک سے زیادہ سال حمل ٹھر نے سے بچاتی ہے؟	YES ……………… ……… …. …. ……. ………… ہاں 1<br>NO ……………… ……… …. …. ……. ………… نہیں 2<br>REFUSED ……… ……… …. …. ……. ……انکار کردیہ 99	
6	Pill. PROBE: Women can take a pill every day to avoid become pregnant.<br>گولیا ں: خاتون حمل سے بچنے کے لیے روز گولی کھا سکتی ہے.	YES ……………… ……… …. …. ……. ………… ہاں 1<br>NO ……………… ……… …. …. ……. ………… نہیں 2<br>REFUSED ……… ……… …. …. ……. ……انکار کردیہ 99	
7	Condom. PROBE: Men can put a rubber sheath on their penis before sexual intercourse to prevent pregnancy.<br><br>کنڈوم"ساتھی": حمل سے بچانے کے لیے مرد ہم بستری سے پہلے اپنے عضوتناسل پر ربڑ (کنڈوم) سے بنا غلاف چڑھا سکتا ہے	YES ……………… ……… …. …. ……. ………… ہاں 1<br>NO ……………… ……… …. …. ……. ………… نہیں 2<br>REFUSED ……… ……… …. …. ……. ……انکار کردیہ 99	

5. FAMILY PLANNING

5. خاندانی منصوبہ بندی

NO.	QUESTIONS AND FILTERS	CODING CATEGORIES	SKIP
8	Lactational Amenorrhea Method (LAM).<br>PROBE: Women can exclusively breastfeed their children until 6 months of age to avoid pregnancy.<br>لیکیٹیشنل امینوریا طریقہ کار(LAM) خواتین صرف اور صرف اپنا دودھ 6 ماہ تک بچوں کو پلاکر حمل کو روک سکتی ہیں	YES .... ہاں 1<br>NO .... نہیں 2<br>REFUSED ....انکار کردیہ 99	
9	Rhythm/Calendar Method. PROBE: To avoid pregnancy, women do not have sexual intercourse on the days of the month they think they can get pregnant.<br>ریدھم/کیلنڈر کا طریقہ: حمل سے بچنے کے لیے خواتین مہینے کے ان دنوں میں جنسی تعلقات سے اجتناب کرتی ہیں جن میں وہ سمجھتی ہیں کہ حاملہ ہوسکتی ہیں.	YES .... ہاں 1<br>NO .... نہیں 2<br>REFUSED ....انکار کردیہ 99	
10	Withdrawal. PROBE: Men can be careful and pull out before climax.<br>باہر نکالنا: مرد انزال سے پہلے عضو تناسل کو باہر نکال کر محطاط ہوسکتے ہیں	YES .... ہاں 1<br>NO .... نہیں 2<br>REFUSED ....انکار کردیہ 99	
11	Emergency Contraception. PROBE: As an emergency measure, within five days after they have unprotected prevent sexual intercourse, women can take special pills to pregnancy.<br>فوری طور پر حمل کو ضائع کرنے والی دوا: ہنگامی طور پر غیر محفوظ جنسی تعلقات کے بعد پانچ دن کے اندر ،خواتین حمل کی روک تھام کے لیے خاص گولیاں کھا سکتی ہیں.	YES .... ہاں 1<br>NO .... نہیں 2<br>REFUSED ....انکار کردیہ 99	
12	Have you heard of any other ways or methods that women or men can use to avoid pregnancy?<br>کیا آپ نے کسی دوسرے طریقے کے بارے میں سنا ہے جو کہ خواتین یا مرد حمل سے بچنے کے لیے استعمال کرسکتے ہیں.	YES . ہاں 1<br>__________________<br>(SPECIFY) دیگروضاحت کریں<br>NO .... نہیں 2<br>REFUSED ....انکار کردیہ 99	
502	IF "NO" TO ALL OF 501, SKIP.<br>Where did you learn about **any** of these family planning methods?<br>SELECT ALL THAT APPLY<br>اگر 501 میں سب پر نہیں کا جواب ہوتو 502 کو چھوڑ دیں<br>آپ نے کہاں سے ان میں سے کسی بھی مانع حمل کے طریقے کے بارے میں معلومات حاصل کی ان تما م جوابات کو نشان کریں جو بتائے گئے	NEIGHBOR ہمسایہ 1<br>FRIEND دوست 2<br>RELATIVE رشتہ دار 3<br>HUSBAND شوہر 4<br>DOCTOR ڈاکٹر 5<br>NURSE/MIDWIFE WORKING AT HEALTH FACILITY 6<br>نرس/میڈوائف جو مرکز صحت میں کام کرتی ہے<br>NURSE/MIDWIFE MAKING A HOME VISIT 7<br>نرس/میڈوارف جو گھر میں آتی ہے<br>LADY HEALTH WORKER لیڈی ہیلتھ ورکر 8<br>FIELD WORKER MAKING A HOME VISIT 9<br>فیلڈورکر جو گھر کا دورہ کرتی ہے<br>PHARMACIST فارمسٹ 10<br>SCHOOL اسکول 11<br>NEWSPAPER/ MAGAZINE /BOOK اخبار یا میگزین/کتاب 12<br>TELEVISION /RADIO ٹی وی/ریڈیو 13<br>INTERNET انٹرنیٹ 14<br>SOCIAL MEDIA سوشل میڈیا 15<br>NO ONE کوئی نہیں 16<br>BILLBOARD بل بورڈ 17<br>OTHER ______________ دیگر وضاحت کریں 96<br>(SPECIFY)<br>DON'T KNOW .... معلوم نہیں 88<br>REFUSED . انکار کردیا 99	

5. FAMILY PLANNING

5. خاندانی منصوبہ بندی

NO.	QUESTIONS AND FILTERS	CODING CATEGORIES	SKIP
503	According to Pakistani law, under what condition(s) is it legal to have an induced abortion?<br>CIRCLE ALL THOSE MENTIONED SPONTANEOUSLY BY THE RESPONDENT<br>پاکستانی قانون کے مطابق کن حالات میں اپنی مرضی سے حمل کو ضائع کرنا قانونی طور پر جائزہے ؟<br>جواب دہندہ کی طرف سے دیئے گئے تمام جوابات پر نشان لگائیں؟	On request درخواست پہ 1<br>Economic or social reasons اقتصادی اور سماجی وجوہات پر 2<br>Foetal impairment بچہ دانی میں خرابی 3<br>Rape زنا کاری 4<br>Incest قریبی رشتوں میں جنسی ملاپ 5<br>Intellectual or cognitive disability of the woman خاتون کی عقلی یا عملی معذوری 6<br>Mental Health دماغی حالت خراب ہو 7<br>Physical Health جسمانی صحت خراب ہو 8<br>Under no conditions/ NOT LEGAL کسی صورت میں نہیں/غیر قانونی 9<br>Mother's life in danger ماں کی جان کو خطرہ ہو 10<br>OTHER (SPECIFY) ______ دیگر وضاحت کریں 96<br>DON'T KNOW .... معلوم نہیں 88<br>REFUSED انکار کردیا 99	9 → 505<br>88 → 505<br>99 → 505
504	According to Pakistani law, up to how many weeks of pregnancy is it permitted to have an induced abortion?<br>پاکستانی قانون کے مطابق حمل ٹھہر نےکے کتنے ہفتے بعد حمل کو ضائع کرنے کی اجازت ہے ؟	WEEKS ہفتے [ ][ ]<br>DON'T KNOW معلوم نہیں 88<br>REFUSED انکار کردیا 99	
505	What methods for induced abortion have you ever heard of?<br>SELECT ALL THAT APPLY.<br>حمل ضائع کروانے کے وہ کونسے طریقے ہیں جن کے بارے میں آپ نے کبھی سنا ہے؟<br>تمام جوابات پر نشان لگائیں جو لاگو ہوتے ہیں۔	SURGERY/OPERATION سرجری/آپریشن 1<br>TABLETS/PILLS/MEDICATION ABORTION (MA) گولیاں/دوائیوں کے ذریعے اسقاط حمل<br>HOMEMADE CONCOCTION گھریلو اجزاء 3<br>INSERTING HERBS OR OBJECT IN WOMB بچہ دانی میں جڑی بوٹیاں یا اور چیز یں رکھنا 4<br>MASSAGE مالش کا طریقہ 5<br>NONE کوئی نہیں 6<br>INJECTION انجکشن 7<br>OTHER ______ (SPECIFY) دیگر وضاحت 96<br>REFUSED انکار کردیا 99	
506	Have you ever been given information about having an induced abortion for an unwanted pregnancy?<br>کیا آپ کو کبھی کسی نےغیرارادی زچگی کو ضائع کرنےکے طریقوں کے بارے میں بتایا ؟	YES ہاں 1<br>NO نہیں 2<br>DON'T KNOW معلوم نہیں 88<br>REFUSED انکار کردیا 99	2, 88, 99 → 508
507	Where did you get information about induced abortion?<br>SELECT ALL THAT APPLY<br>آپ نے اپنی مرضی سے حمل ضائع کروانے کے بارے میں معلومات کہاں سے حاصل کی؟<br>تمام جوابات پر نشان لگائیں جو لاگو ہوتے ہیں۔	NEIGHBOR ہمسایہ 1<br>FRIEND دوست 2<br>RELATIVE رشتہ دار 3<br>HUSBAND شوہر 4<br>DOCTOR ڈاکٹر 5<br>NURSE/MIDWIFE WORKING AT HEALTH FACILITY نرس/میڈوائف جو مرکز صحت میں کام کرتی ہے 6<br>NURSE/MIDWIFE MAKING A HOME VISIT نرس/میڈوارف جو گھر میں آتی ہے 7<br>LADY HEALTH WORKER لیڈی ہیلتھ ورکر 8<br>FIELD WORKER MAKING A HOME VISIT فیلڈورکر جو گھر کا دورہ کرتی ہے 9<br>PHARMACIST فارمسٹ 10<br>SCHOOL اسکول 11<br>NEWSPAPER/ MAGAZINE /BOOK اخبار یا میگزین/کتاب 12<br>TELEVISION /RADIO ٹی وی/ریڈیو 13<br>INTERNET انٹرنیٹ 14<br>SOCIAL MEDIA سوشل میڈیا 15<br>NO ONE کوئی نہیں 16<br>OTHER ______ (SPECIFY) دیگر وضاحت کریں 96<br>DON'T KNOW .... معلوم نہیں 88<br>REFUSED انکار کردیا 99	

<table>
<tr><th colspan="5">5. FAMILY PLANNING
5. خاندانی منصوبہ بندی</th></tr>
<tr><th>NO.</th><th>QUESTIONS AND FILTERS</th><th colspan="2">CODING CATEGORIES</th><th>SKIP</th></tr>
<tr><td>508</td><td>In the past 2 years has any health worker come to talk with you about preventing pregnancy or terminating an unintended pregnancy?
پچھلے دو سالوں میں کیا کسی صحت کے کارکن نے دورہ کر کے آپ کو حمل سے بچاؤ یا غیر ارادی حمل کو ختم کرنے کےبارے میں بات چیت کی؟</td><td colspan="2">YES ہاں 1
NO نہیں 2
DON'T KNOW معلوم نہیں 88
REFUSED انکار کردیا 99</td><td>2, 88, 99 → 524</td></tr>
<tr><td>509</td><td>What organization(s) was/were the health worker(s) from?
کس ادارے سے وہ صحت کا کارکن آیا تھا /آئی تھی؟
SELECT ALL THAT APPLY
تمام جوابات پر نشان لگائیں جو لاگو ہوتے ہیں۔</td><td colspan="2">WILLOWS ویلوز 1
GOVERNMENT گورنمنٹ 2
OTHER, SPECIFY دیگر وضاحت 96
DON'T KNOW معلوم نہیں 88
REFUSED انکار کردیا 99</td><td></td></tr>
<tr><td></td><td></td><td>WILLOWS</td><td>OTHERS</td><td></td></tr>
<tr><td>510</td><td>CHECK 509: WILLOWS IF 1 & OTHERS IF 2 OR 96
In the last 2 years, how many times did someone come to visit to provide counseling or information?
509چیک کریں اگر ویلوس کا 1 یا 2 یا دیگر کا 96 ہو
پچھلے دو سالوں میں معلومات یا مشورے فراہم کرنے کتنی بار کوئی آیا تھا؟</td><td>1 TIME ایک دفعہ 1 (SKIP TO 512)
2 TIMES دو دفعہ 2
3-5 TIMES تین سے پانچ مرتبہ 3
MORE THAN 5 TIMES پانچ سے زیادہ مرتبہ 4
DON'T KNOW معلوم نہیں معلوم نہیں 88
REFUSED انکار کردیا 99</td><td>1 TIME ایک دفعہ 1 (SKIP TO 512)
2 TIMES دو دفعہ 2
3-5 TIMES تین سے پانچ مرتبہ 3
MORE THAN 5 TIMES پانچ سے زیادہ مرتبہ 4
DON'T KNOW معلوم نہیں 88
REFUSED انکار کردیا 99</td><td></td></tr>
<tr><td>511</td><td>Did the same person come to visit you more than once?
کیا وہی شخص آپ سے ایک سے زیادہ دفعہ ملنے آئی /آیا؟</td><td>YES ہاں 1
NO نہیں 2
DON'T KNOW معلوم نہیں 88
REFUSED انکار کردیا 99</td><td>YES ہاں 1
NO نہیں 2
DON'T KNOW معلوم نہیں 88
REFUSED انکار کردیا 99</td><td></td></tr>
<tr><td>512</td><td>What gender was the person(s) who came to visit?
ان لوگوں کی جنس کیا تھی جو آپ سے ملنے آئے تھے؟</td><td>MALE مرد 1
FEMALE عورت 2
BOTH MALE AND FEMALE مرد اور عورت دونوں 3
DON'T KNOW معلوم نہیں 88
REFUSED انکار کردیا 99</td><td>MALE مرد 1
FEMALE عورت 2
BOTH MALE AND FEMALE مرد اور عورت دونوں 3
DON'T KNOW معلوم نہیں 88
REFUSED انکار کردیا 99</td><td></td></tr>
<tr><td>513</td><td>What topics did the person(s) talk with you about during the home visit?
گھر کے دورے کے دوران اس شخص نے آپ کے ساتھ کن موضوعات پر بات کی؟
SELECT ALL THAT APPLY
تمام جوابات پر نشان لگائیں جو لاگو ہوتے ہیں۔
DO NOT READ RESPONSES OUT LOUD.
جوابات کواونچی آواز میں پڑھ کر سنانے کی ضرورت نہیں</td><td>PREGNANCY حمل 1
FAMILY PLANNING خاندانی منصوبہ بندی 2
ABORTION اپنی مرضی سے حمل ضائع کروانا 3
SEXUALLY TRANSMITTED) INFECTIONS (STIs) ایس - ٹی -آئی(جنسی میلاپ سے منتقل شدہ انفیکشن 4
PAP SMEAR پیپ سمیئر 5
BREAST EXAM چھاتی کا معائنہ 6
INFERTILITY 7
OTHER (SPECIFY) دیگر وضاحت 96
DON'T KNOW معلوم نہیں 88
REFUSED انکار کردیا 99</td><td>PREGNANCY حمل 1
FAMILY PLANNING خاندانی منصوبہ بندی 2
ABORTIONS اپنی مرضی سے حمل ضائع کروانا 3
SEXUALLY TRANSMITTED) INFECTIONS (STIs) ایس - ٹی -آئی(جنسی میلاپ سے منتقل شدہ انفیکشن 4
PAP SMEAR پیپ سمیئر 5
BREAST EXAM چھاتی کا معائنہ 6
INFERTILITY 7
OTHER (SPECIFY) دیگر وضاحت 96
DON'T KNOW معلوم نہیں 88
REFUSED انکار کردیا 99</td><td></td></tr>
<tr><td>514</td><td>Were you counseled or provided information on using family planning methods?
کیا آپ کو خاندانی منصوبہ بندی کے طریقوں کے بارے میں مشورہ یا معلومات فراہم کردی گئی تھی؟</td><td>YES ہاں 1
NO نہیں 2
DON'T KNOW معلوم نہیں 88
REFUSED انکار کردیا 99
(2, 88, 99: SKIP TO 516)</td><td>YES ہاں 1
NO نہیں 2
DON'T KNOW معلوم نہیں 88
REFUSED انکار کردیا 99
(2, 88, 99: SKIP TO 516)</td><td></td></tr>
</table>

5. FAMILY PLANNING

5. خاندانی منصوبہ بندی

NO.	QUESTIONS AND FILTERS	CODING CATEGORIES		SKIP
515	Which method(s) were you counseled/given information on?<br>CIRCLE ALL MENTIONED.<br>کن طریقوں کے بارے میں آپ سے مشاورت/معلومات دی گئی؟<br>(جتنے جواب آئیں سب کو نشان لگائیں)	FEMALE STERILIZATION 1<br>خاتون کی نس بندی<br>MALE STERILIZATION 2<br>مرد وں کی نس بندی<br>IUD آئی یوڈی 3<br>INJECTABLES ..... انجیکشن 4<br>IMPLANTS .... امپلانٹس 5<br>PILL گولیاں 6<br>CONDOM کنڈوم 7<br>EMERGENCY CONTRACEPTION 11<br>ایمرجنسی مانع حمل<br>STANDARD DAYS/CALENDAR METH 12<br>دنوں کے حساب کا طریقہ/کیلنڈر<br>LACTATIONAL AMEN. METHOD 13<br>دودھ پلانے کے دوران<br>WITHDRAWAL ہٹانے والاطریقہ 15<br>NONE نہیں 98<br>DON'T KNOW/REMEMBER 88<br>معلوم نہیں /یاد نہیں<br>REFUSED انکار کردیا 99<br>OTHER (Specify) دیگر وضاحت کریں 96	FEMALE STERILIZATION 1<br>خاتون کی نس بندی<br>MALE STERILIZATION 2<br>مرد وں کی نس بندی<br>IUD آئی یوڈی 3<br>INJECTABLES انجیکشن 4<br>IMPLANTS امپلانٹس 5<br>PILL گولیاں 6<br>CONDOM کنڈوم 7<br>EMERGENCY CONTRACEPTI 11<br>ایمرجنسی مانع حمل<br>STANDARD DAYS/CAI .... 12<br>دنوں کے حساب کا طریقہ/کیلنڈر<br>LACTATIONAL AMEN. METHC 13<br>دودھ پلانے کے دوران<br>WITHDRAWAL ہٹانے والاطریقہ 15<br>NONE نہیں 98<br>DON'T KNOW/REMEMBER 88<br>معلوم نہیں /یاد نہیں<br>REFUSED انکار کردیا 99<br>OTHER (Specify)<br>دیگر وضاحت کریں 96	
516	Who else (besides yourself) participated in the information sessions?<br>آپ کے علاوہ اور کس نے معلوماتی گروپ سیشن سے معلومات حاصل کی؟<br>CIRCLE ALL MENTIONED.<br>(جتنے جو اب ائیں سب کو نشان لگائیں)	HUSBAND شوہر 1<br>MOTHER ماں 2<br>DAUGHTER بیٹی 3<br>SON بیٹا 4<br>FRIEND/NEIGHBOR<br>دوست/ہمسایہ 5<br>OTHERS دیگر 6<br>NO ONE ELSE کوئی اور نہیں 7<br>REFUSED انکار کردیا 99	HUSBAND ....شوہر 1<br>MOTHER ماں 2<br>DAUGHTER بیٹی 3<br>SON بیٹا 4<br>FRIEND/NEIGHBOR<br>دوست/ہمسایہ 5<br>OTHERS دیگر 6<br>NO ONE ELSE کوئی اور نہیں 7<br>REFUSED انکار کردیا 99	
517	Did she/he ever show you examples of contraceptive methods?<br>کیا انہوں نے کبھی آپکو حمل سے بچاؤ کے لیے استعمال ہونے والے طریقہ کار مثال دے کر سمجھایا؟	YES ہاں 1<br>NO نہیں 2<br>DON'T KNOW معلوم نہیں 88<br>REFUSED انکار کردیا 99	YES ہاں 1<br>NO نہیں 2<br>DON'T KNOW معلوم نہیں 88<br>REFUSED انکار کردیا 99	
518	Did she/he ever give you brochures or materials about contraceptive methods?<br>کیا انہوں نے کبھی آپکو کتابچہ یا مواد دی جس میں مانع حمل کے طریقوں کا ذکر کیا گیا ہو؟	YES ہاں 1<br>NO نہیں 2<br>DON'T KNOW معلوم نہیں 88<br>REFUSED انکار کردیا 99	YES ہاں 1<br>NO نہیں 2<br>DON'T KNOW معلوم نہیں 88<br>REFUSED انکار کردیا 99	
519	Did she/he ever discuss induced abortion with you?<br>کیا انہوں نے اپنی مرضی سے حمل ضائع کروانے کے بارے میں آپ سے بات چیت کی؟	YES ہاں 1<br>NO نہیں 2<br>DON'T KNOW /Cant Remember<br>معلوم نہیں/ یاد نہیں 88<br>REFUSED انکار کردیا 99<br>(SKIP TO 521)	YES ہاں 1<br>NO نہیں 2<br>DON'T KNOW /Cant Remember معلوم نہیں/ یاد نہیں 88<br>REFUSED انکار کردیا 99<br>(SKIP TO 521)	
520	Did she/he ever provide brochures or materials about induced abortion to you?<br>کیا انہوں نے نے اپنی مرضی سے حمل ضائع کروانے کے بارے میں کتا بچہ یا مواد آپکو دی تھی؟	YES ہاں 1<br>NO نہیں 2<br>DON'T KNOW معلوم نہیں 88<br>REFUSED انکار کردیا 99	YES ہاں 1<br>NO نہیں 2<br>DON'T KNOW معلوم نہیں 88<br>REFUSED انکار کردیا 99	

5. FAMILY PLANNING

5. خاندانی منصوبہ بندی

NO.	QUESTIONS AND FILTERS	CODING CATEGORIES		SKIP
521	Did she/he ever refer you to a health facility for family planning or reproductive health issues?<br>کیا انہوں نے کبھی آپکو خاندانی منصوبہ بندی یا تولیدی صحت کے مسائل کے لئے مرکز صحت کی طرف بھیجا تھا؟	YES ہاں 1<br>NO نہیں 2<br>DON'T KNOW معلوم نہیں 88<br>REFUSED انکار کردیا 99<br>(SKIP TO 523)	YES ہاں 1<br>NO نہیں 2<br>DON'T KNOW معلوم نہیں 88<br>REFUSED انکار کردیا 99<br>(SKIP TO 523)	
522	Did you go there based on this referral?<br>کیا آپ انکے مشورے کے مطابق وہاں گئی تھیں؟	YES ہاں 1<br>NO نہیں 2<br>DON'T KNOW معلوم نہیں 88<br>REFUSED انکار کردیا 99	YES ہاں 1<br>NO نہیں 2<br>DON'T KNOW معلوم نہیں 88<br>REFUSED انکار کردیا 99	
523	Do you think the information provided by the field worker(s) was reliable?<br>کیا آپکے خیال میں ہیلتھ ورکر کی طرف سے فراہم کردہ معلومات قابل اعتماد ہے؟	YES ہاں 1<br>SOMEWHAT کسی حد تک 2<br>NO نہیں 3<br>DON'T KNOW معلوم نہیں 88<br>REFUSED انکار کردیا 99	YES ہاں 1<br>SOMEWHAT کسی حد تک 2<br>NO نہیں 3<br>DON'T KNOW معلوم نہیں 88<br>REFUSED انکار کردیا 99	
524	In the past 2 years, have you visited a health facility for care for yourself (or your children)?<br>پچھلے دو سالوں میں آپ کسی مرکز صحت میں اپنے لیے یا اپنے بچے کے لیے گئے؟	YES ہاں 1<br>NO نہیں 2<br>REFUSED انکار کردیا 99		1 → 526
525	Why have you not visited a health facility in the past 2 years?<br>پچھلے دو سالوں میں آپ نے کسی مرکز صحت کا دورہ کیوں نہیں کیا ؟<br>SELECT ALL THAT APPLY. (ایک سے زیادہ جوابات دے سکتے ہیں)	TOO FAR بہت دور ہے 1<br>TOO EXPENSIVE بہت مہنگا ہے 2<br>NO NEED ضرورت نہیں 3<br>OTHER, SPECIFY دیگر (وضاحت کریں) 96<br>REFUSED انکار کردیا 99		→ 538
526	During any visit in the past 2 years, did any health worker/family walfare worker at the health facility speak to you about family planning methods?<br>گزشتہ 2 سالوں میں مرکز صحت یا فیملی ویلفیئر کے کسی نمائندے نے کسی بھی دورے کے دوران خاندانی منصوبہ بندی کے بارے میں آپ سے بات کی؟	YES ہاں 1<br>NO نہیں 2<br>REFUSED انکار کردیا 99		2, 99 → 538
527	Which health facility did you receive family planning information from the last time?<br>کس مرکز صحت سے آپ نے خاندانی منصوبہ بندی کی معلومات آخری ٹائم حاصل کی تھی؟<br>PROBE TO IDENTIFY THE TYPE OF FACILITY.<br>سہولت کی قسم کی شناخت کے لئے پوچھ گچھ کریں۔	PUBLIC SECTOR پبلک/سرکاری سیکٹر<br>11 GOVT. HOSPITAL/RHSC سرکاری ہسپتال 11<br>12 RURAL HEALTH CENTER, MCH دیہی ہیلتھ سینٹر 12<br>13 MCH CENTRE.. ایم سی ایچ 13<br>14 FAMILY HEALTH CENTRE/FWA فیملی ہیلتھ سینٹر/ایف ڈبلیو اے 14<br>17 HEALTH HOUSE (LHV) ایل ایچ وی کا ہیلتھ ہاؤس 17<br>18 BASIC HEALTH UNIT بنیادی ہیلتھ یونٹ 18<br>21 OTHER PUBLIC SPECIFY دیگر سرکاری وضاحت کریں 21<br>PRIVATE/NGO MEDICAL SECTOR پرائیوٹ میڈیکل سیکٹر<br>22 PRIVATE/NGO/HOSPITAL/CLINIC پرائیوٹ/ہسپتال/کلینک 22<br>OTHER PRIVATE MEDICAL دیگر پرائیوٹ میڈیکل<br>96 OTHER (SPECIFY) دیگر وضاحت کریں 96<br>88 DON'T KNOW معلوم نہیں 88<br>99 REFUSED انکار کردیا 99		

5. FAMILY PLANNING

5. خاندانی منصوبہ بندی

NO.	QUESTIONS AND FILTERS	CODING CATEGORIES	SKIP
527B	What was the name of the health facility?<br>صحت کے ادارے کا کیانام تھا؟<br>یہ سوال اس صورت میں کریں اگر 527 پر کوئی آپشن نہیں بتایا گیا ہو	______________________	
528	Who provided you with this information or counseling (during the last visit)?<br>SELECT ALL THAT APPLY<br>آپ کو یہ معلومات یا مشورے کس نے دیا تھا (آخری دورے کے دوران)؟<br>(جتنے جواب آئیں سب کو نشان لگائیں)	DOCTOR ڈاکٹر 1<br>NURSE نرس 2<br>MIDWIFE مڈوائف 3<br>LADY HEALTH WORKER/OUTREACH/ PEER EDUCATOR لیڈی ہیلتھ ورکر/ہم مرتبہ معلم 4<br>PHARMACIST فارماسسٹ 5<br>OTHER ______ (SPECIFY) دیگر وضاحت 96<br>DON'T KNOW معلوم نہیں 88<br>REFUSED انکار کردیا 99	
529	Which method(s) were you counseled on the last time?<br>CIRCLE ALL MENTIONED. (جتنے جواب آئیں سب کو نشان لگائیں)<br>آخری مرتبہ آپ کو کونسا طریقہ /طریقے کے بارے میں مشورہ دیاگیا تھا؟	FEMALE STERILIZATION خاتون کی نس بندی 1<br>MALE STERILIZATION مرد وں کی نس بندی 2<br>IUD آئی یو ڈی 3<br>INJECTABLES انجیکشن 4<br>IMPLANTS امپلانٹس 5<br>PILL گولیاں 6<br>CONDOM کونڈوم 7<br>STANDARD DAYS/CALANDER METHOD دنوں کے حساب کا طریقہ/کیلنڈر 11<br>LACTATIONAL AMEN. METHOD دودھ پلانے کے دوران 12<br>WITHDRAWAL ہٹانے والاطریقہ 14<br>OTHER ______ (SPECIFY) دیگر وضاحت 96<br>NONE نہیں 17<br>REFUSED انکار کردیا 99	
531	Did your husband participate in the counseling/information session the last time?<br>کیا آپ کے شوہر آخری بار مشاورت /معلومات کے سیشن میں آئے؟	YES ہاں 1<br>NO نہیں 2<br>REFUSED انکار کردیا 99	
532	In which language(s) were you counseled the last time?<br>SELECT ALL THAT APPLY.<br>(جتنے جواب آئیں سب کو نشان لگائیں)<br>پچھلے آخری سیشن میں کس زبان میں آپ کو مشورہ دیا گیا تھا؟	URDU اردو 1<br>SINDHI سندھی 2<br>PUSHTU پشتو 3<br>PUNJABI پنجابی 4<br>BALOCHI بلوچی 5<br>ENGLISH انگریزی 6<br>OTHER دیگر وضاحت 96<br>DON'T REMEMBER/DON'T KNOW یاد نہیں/معلوم نہیں 88<br>REFUSED انکار کردیا 99	
533	Did the person who counseled you ask questions about your health?<br>جس نے آپ کی مشاورت کی ،کیا اس نے آپ سے صحت کے متعلق بھی سوالات کیے؟	YES ہاں 1<br>NO نہیں 2<br>DON'T KNOW معلوم نہیں 88<br>REFUSED انکار کردیا 99	
534	Were you counseled on what would happen if you chose NOT to use family planning?<br>کیا آپ کو بتایا گیا تھا کہ اگر آپ خاندانی منصوبہ بندی کا استعمال نہیں کریں گی تو کیا ہوگا؟	YES ہاں 1<br>NO نہیں 2<br>DON'T KNOW معلوم نہیں 88<br>REFUSED انکار کردیا 99	

5. FAMILY PLANNING

5. خاندانی منصوبہ بندی

NO.	QUESTIONS AND FILTERS	CODING CATEGORIES	SKIP
535	Do you feel that you were given enough information about family planning?<br>کیا آپ کو لگتا ہے کہ آپ کو خاندانی منصوبہ بندی کی کافی معلومات دی گئی ہیں؟	YES ہاں 1<br>NO نہیں 2<br>DON'T KNOW معلوم نہیں 88<br>REFUSED انکار کردیا 99	
536	Are you satisfied with the FAMILY PLANNING COUNSELING services you received at your last visit?<br>کیا آپ آخری دورے کے وقت وصول کی گئی خآندانی منصوبہ بندی کی مشاورت کی خدمات سے مطمئن ہیں؟	FULLY SATISFIED مکمل طورپر تسلی بخش 1<br>SOMEWHAT SATISFIED کسی حد تک تسلی بخش 2<br>NOT SATISFIED غیر تسلی بخش 3<br>DON'T KNOW معلوم نہیں 88<br>REFUSED انکار کردیا 99	
537	Do you think the family planning information provided by this service provider is reliable?<br>کیا صحت کی سہولیات فراہم کرنے والے کی جانب سے دی گئی معلومات آپ کے لیے قابل اعتماد ہے؟	YES ہاں 1<br>SOMEWHAT کسی حد تک 2<br>NO نہیں 3<br>DON'T KNOW معلوم نہیں 88<br>REFUSED انکار کردیا 99	
538	Would you recommend services from this provider to a friend or a relative in a similar situation as you?<br>کیا صحت کی سہولیات فراہم کرنے والے کی طرف سے تجویز کی گئی خدمات کو آپ اپنی کسی دوست یا رشتہ دار کو جو آپ جیسی صورت حال سے سے دوچار ہوں دینا چاہیں گی؟	YES ہاں 1<br>NO نہیں 2<br>DON'T KNOW معلوم نہیں 88<br>REFUSED انکار کردیا 99	
539	Have you <u>ever</u> used anything or tried in any way to delay or avoid getting pregnant?<br>کیا آپ نے کبھی حمل سے بچنے یا تاخیر کے لیے کوئی طریقہ استعمال کیا؟	YES ہاں 1<br>NO نہیں 2<br>REFUSED انکار کردیا 99	 → 540 (NO, REFUSED)
540	Which method(s) have you ever used?<br>آپ نے کون کون سے طریقے استعمال کئے؟<br>CIRCLE ALL MENTIONED.<br>جوا ب دہندہ کے بتائے گئے ہر طریقے پر نشان لگائیں؟<br>IF MORE THAN ONE METHOD MENTIONED, FOLLOW SKIP INSTRUCTION FOR HIGHEST METHOD IN LIST.<br>اگر ایک سے زیادہ طریقے بتائیں ہیں تو جو فہرست میں سب سے اوپر والا طریقہ ہے اس پر نشان لگائیں؟	FEMALE STERILIZATION خاتون کی نس بندی 1<br>MALE STERILIZATION مرد وں کی نس بندی 2<br>IUD آئی‌یوڈی 3<br>INJECTABLES انجیکشن 4<br>IMPLANTS امپلانٹس 5<br>PILL گولیاں 6<br>CONDOM کونڈوم 7<br>EMERGENCY CONTRACEPTION فوری مانع حمل ادویات 9<br>STANDARD DAYS METHOD/CALANDER دنوں کے حساب کا طریقہ/کیلنڈر 11<br>LACTATIONAL AMEN. METHOD دودھ پلانے کے دوران 12<br>WITHDRAWAL ہٹانے والاطریقہ 14<br>OTHER دیگر وضاحت ______ (SPECIFY) 96<br>NO METHOD USED کوئی طریقہ استعمال نہیں کیا 17<br>REFUSED انکار کردیا 99	

5. FAMILY PLANNING

5. خاندانی منصوبہ بندی

NO.	QUESTIONS AND FILTERS	CODING CATEGORIES	SKIP
541	In choosing a contraceptive method, what feature would be most important to you?<br>مانع حمل کا طریقہ منتخب کرتے وقت آپ کس بات کو سب سے زیادہ اہمیت دیتی ہیں<br>SELECT ONE - PROBE FOR MOST IMPORTANT REASON.<br>کسی ایک سب سے زیادہ اہم وجہ کو نشان کریں	HOW EFFECTIVE IT IS AT PREVENTING PREGNANCY حمل کو روکنے میں کتنی موثر ہوگی 1<br>CAN BE USED WITHOUT ANYONE ELSE KNOWING کسی کو بتائے بغیر استعمال کرسکتی ہوں 2<br>THAT IT PROTECTS AGAINST STIs/HIV کیا یہ مجھے STIs/HIVs سے بچائے گا 3<br>NO RISK OF HARMING HEALTH صحت کے لیے نقصان دہ نہ ہو 4<br>NO EFFECT ON REGULAR MONTHLY BLEEDING ماہواری پر منفی اثرات نہ ہوں 5<br>NO UNPLEASANT SIDE EFFECTS کوئی ناخشگوار اثرات نہ ہوں 6<br>EASY TO USE استعمال میں آسان ہو 7<br>EASY TO OBTAIN آسانی سے حاصل ہوسکے 8<br>AFFORDABLE قیمت زیادہ نہیں ہے 9<br>CAN BE USED FOR A LONG TIME WITHOUT NEED TO VISIT CLINIC OR RE-SUPPLY 10<br>آسانی سے لمبے عرصے استعمال ہوسکے اور بار بار کلینک یا حاصل کرنے کے لیے جانا نہ پڑے<br>WILL BE ABLE TO GET PREGNANT WHEN I WANT جب میں چاہوں حاملہ ہو سکتی ہوں 11<br>OTHER ________ دیگر (وضاحت کریں) 96<br>(SPECIFY)<br>DON'T KNOW .... معلوم نہیں 88<br>REFUSED .... انکار کردیا 99	
542	CHECK Q. 539: سوال نمبر 539 چیک کریں:<br>EVER USED FAMILY PLANNING ☐ کبھی خاندانی منصوبہ بندی کا طریقہ استعمال کیا	NEVER USED FAMILY PLANNING ☐ کبھی خاندانی منصوبہ بندی کا طریقہ استعمال نہیں کیا →	548
543	CHECK Q. 420: سوال نمبر 420 چیک کریں:<br>NOT PREGNANT OR UNSURE ☐ حاملہ نہیں ہے یا یقین نہیں ہے	PREGNANT ☐ حاملہ ہے →	555
544	Are you or your husband currently doing something or using any method to delay or avoid getting pregnant?<br>آپ یا آپ کے شوہر اس وقت کوئی طریقہ یا کوئی حمل روکنے والی اشیاء کو استعمال کر رہے ہیں جس سے آپکے حمل روکنے میں مدد ملے؟	YES ہاں 1<br>NO نہیں 2<br>DON'T KNOW معلوم نہیں 88<br>REFUSED انکار کردیا 99	547
545	Which method(s) are you or your husband currently using?<br>فی الوقت آپ اور آپ کے شوہر کونسا طریقہ استعمال کر رہے ہیں؟<br>(تمام ممکنہ جوابات پر نشان لگائیں)<br>IF MORE THAN ONE METHOD MENTIONED, FOLLOW SKIP INSTRUCTION FOR HIGHEST METHOD IN LIST.<br>اگر ایک سے زیادہ طریقے بتائیں ہیں تو جو فہرست میں سب سے اوپر والا طریقہ ہے اس پر نشان لگائیں؟	FEMALE STERILIZATION خاتون کی نس بندی 1<br>MALE STERILIZATION مرد وں کی نس بندی 2<br>IUD آئی یو ڈی 3<br>INJECTABLES انجیکشن 4<br>IMPLANTS امپلانٹس 5<br>PILL گولیاں 6<br>CONDOM کونڈوم 7<br>EMERGENCY CONTRACEPTION (ECP) فوری مانع حمل ادویات 9<br>STANDARD DAYS /CALANDER METHOD دنوں کے حساب کا طریقہ/کیلنڈر 11<br>LACTATIONAL AMEN. METHOD دودھ پلانے کے دوران 12<br>WITHDRAWAL ہٹانے والا طریقہ 14<br>OTHER ________ دیگر (وضاحت کریں) 96<br>(SPECIFY)<br>NO METHOD USED کوئی طریقہ استعمال نہیں کیا 88<br>REFUSED انکار کردیا 99	1, 2 → 556<br>88, 99 → 546

5. FAMILY PLANNING
5. خاندانی منصوبہ بندی

NO.	QUESTIONS AND FILTERS	CODING CATEGORIES	SKIP
546	Since what month and year have you been using (CURRENT METHOD) without stopping?<br>کونسے مہینے اور سال سے آپ اس موجودہ طریقہ کار کو ترک کئے بغیر استعمال کر رہی ہیں؟	MONTH مہینہ [ ][ ]<br>YEAR سال [ ][ ][ ][ ]<br>DON'T KNOW معلوم نہیں 88/8888<br>REFUSED انکار کردیا 99/9999<br>NOT CONSISTENT USE مسلسل استعمال میں نہیں ہے 1	
547	Are you currently breastfeeding to delay or avoid getting pregnant?<br>کیا آپ فی الحال تاخیر کرنے یا حاملہ ہونے سے بچنے کے لئے دودھ پلاتی ہیں؟	YES ہاں 1<br>NO نہیں 2<br>DON'T KNOW معلوم نہیں 88<br>REFUSED انکار کردیا 99	
548	CHECK Q. 424: سوال نمبر 424 چیک کریں.<br>GAVE BIRTH IN PAST 6 MONTHS پچھلے 6 ماہ میں جنم دیا [ ] ↓	DID NOT GIVE BIRTH IN PAST 6 MONTHS پچھلے 6 ماہ میں جنم نہیں دیا [ ] →	553
549	Did you breastfeed your most recent baby at any time after giving birth?<br>پیدائش کے بعد کسی بھی وقت آپ نے اپنے حالیہ بچے کو دودھ دیاتھا؟	YES ہاں 1<br>NO نہیں 2<br>REFUSED انکار کردیا 99	2, 99 → 552
550	Are you still breastfeeding this baby?<br>کیا آپ ابھی بھی اس بچے کو دودھ پلاتی ہے ؟	YES ہاں 1<br>NO نہیں 2<br>REFUSED انکار کردیا 99	2, 99 → 552
551	Have you given the baby anything but breastmilk since he/she was born?<br>کیا بچے کی پیدائش سے اب تک اپنے دودھ کے علاوہ کچھ دیا تھا؟	YES ہاں 1<br>NO نہیں 2<br>REFUSED انکار کردیا 99	
552	Have your period returned?<br>کیا آپکو دوبارہ حیض/ماہواری آئی ہے؟	YES ہاں 1<br>NO نہیں 2<br>REFUSED انکار کردیا 99	
553	CHECK Q. 538: سوال نمبر 538 چیک کریں<br>EVER USED FAMILY PLANNING کبھی خاندانی منصوبہ بندی کا طریقہ استعمال کیا [ ] ↓	NEVER USED FAMILY PLANNING کبھی خاندانی منصوبہ بندی کا طریقہ استعمال نہیں کیا [ ] →	579

5. FAMILY PLANNING

5. خاندانی منصوبہ بندی

NO.	QUESTIONS AND FILTERS	CODING CATEGORIES	SKIP
554	CHECK Q. 543: سوال نمبر 543 چیک کریں<br><br>NOT CURRENTLY USING FAMILY PLANNING ☐ ↓<br>ابھی خاندانی منصوبہ بندی کا کوئی طریقہ استعمال نہیں کررہیں	CURRENTLY USING FAMILY PLANNING ☐ →<br>ابھی خاندانی منصوبہ بندی کا کوئی طریقہ استعمال کررہی	556
555	You mentioned that you are not currently using a family planning method. Which method(s) did you use last?<br>آپ نےبتایا کہ آپ ابھی خاندانی منصوبہ بندی کا کوئی طریقہ استعال نہیں کر رہی۔ آخری بار کونسا طریقہ استعمال کیا تھا؟<br><br>RECORD ALL MENTIONED.<br>(تمام ممکنہ جوابات پر نشان لگائیں)<br><br>IF MORE THAN ONE METHOD MENTIONED, FOLLOW SKIP INSTRUCTION FOR HIGHEST METHOD IN LIST.<br>اگر ایک سے زیادہ طریقے بیان کئے گئے ہیں تو سب سے زیادہ ا ستعمال ہونے والے طریقہ کار پرنشان لگائیں؟	FEMALE STERILIZATION خاتون کی نس بندی 1<br>MALE STERILIZATION مرد وں کی نس بندی 2<br>IUD آئی یوڈی 3<br>INJECTABLES انجیکشن 4<br>IMPLANTS امپلانٹس 5<br>PILL گولیاں 6<br>CONDOM کونڈوم 7<br>STANDARD DAYS METHOD/CALANDER دنوں کے حساب کا طریقہ/کیلنڈر 11<br>LACTATIONAL AMEN. METHOD دودھ پلانے کے دوران 12<br>WITHDRAWAL ہٹانے والاطریقہ 14<br>OTHER دیگر (وضاحت کریں) ________ 96<br>(SPECIFY)<br>NO METHOD USED کوئی طریقہ استعمال نہیں کیا 17 →<br>REFUSED انکار کردیا 99	 <br><br><br><br><br><br><br><br><br><br><br>579
556	Where did you obtain [THE LAST/CURRENT METHOD] the last time?<br>کس جگہ سے آپ نے آخری بار خاندانی منصوبہ بندی کے گزشتہ/موجودہ طریقہ کار کو حاصل کیا تھا؟<br><br>PROBE TO IDENTIFY THE TYPE OF SOURCE.<br>ذرائع کے قسم کی نشاندہی کرنے کے لئے تحقیق کریں۔	PUBLIC SECTOR پبلک /سرکاری سیکٹر<br>11 GOVT. HOSPITAL/RHSC سرکاری ہسپتال 11<br>12 RURAL HEALTH CENTER, MCH دیہی ہیلتھ سینٹر 12<br>13 MCH............ ایم سی ایچ 13<br>14 FAMILY HEALTH CENTRE/ WELFARE CENTER فیملی ہیلتھ سینٹر /فلاحی سینٹر 14<br>15 MOBILE SERVICE CAMP موبائل سروس کیمپ 15<br>16 LADY HEALTH WORKER لیڈی ہیلتھ ورکر 16<br>17 LH VISITOR لیڈی ہیلتھ وزیٹر 17<br>18 BASIC HEALTH UNIT بنیادی ہیلتھ یونٹ 18<br>19 MALE MOBILIZER مرد موبائلیزر 19<br>20 FWA ایف ڈبلو-اے 20<br>21 OTHER PUBLIC (SPECIFY) دیگر سرکاری وضاحت کریں 21<br>PRIVATE/NGO MEDICAL SECTOR پرائیوٹ میڈیکل سیکٹر<br>22 PRIVATE/NGO/HOSPITAL/CLINIC پرائیوٹ /ہسپتال /کلینک 22<br>23 PHARMACY, CHEMISTS فارمسی /کیمسٹ 23<br>24 PRIVATE DOCTOR پرائیوٹ ڈاکٹر 24<br>25 HOMEOPATH ہومیوپیتھ 25<br>26 DISPENSOR/ COMPOUNDER ڈسپنزر /کمپائونڈر 26<br>28 MOBILE SERVICE CAMP موبائل سروس کیمپ 28<br>27 OTHER PRIVATE MEDICAL دیگر پرائیوٹ میڈیکل 27<br>________ (وضاحت کریں)<br>OTHER SOURCE دیگر ذرائع<br>31 SHOP (NOT PHARMACY/CHEMIST) دکان /فارمیسی /کیمیسٹ 31<br>32 FRIEND/RELATIVE دوست /رشتہ دار 32<br>33 HAKIM حکیم 33<br>34 DAI,TRAD. BIRTH ATTENDANT دائی یا پرانے طریقوں سے ولادت کروانے والی 34<br>96 OTHER (SPECIFY) دیگر وضاحت کریں 96<br>88 DON'T KNOW معلوم نہیں 88<br>99 REFUSED انکار کردیا 99	 <br><br><br><br><br><br><br><br><br><br><br><br><br><br><br><br><br><br><br><br><br><br><br><br><br>557 (codes 31–99)
556b	What was the name of the facility?<br>سہولت حاصل کرنے والی جگہ کانام	________	

5. FAMILY PLANNING

5. خاندانی منصوبہ بندی

NO.	QUESTIONS AND FILTERS	CODING CATEGORIES	SKIP
557	How many kilometers did you have to travel to reach this (SERVICE PROVIDER) to receive (CURRENT METHOD)?<br>آپ کو اس (زیر استعمال موجودہ طریقہ وصول کرنے کے لیے)کتناکلو میٹر/میل سفر طے کرنا پڑتا ہے (سروس دینے والے کے پاس جانے کے لئے)؟<br>[Only for methods 1-11 in Question 545/555]<br>صرف 1-11 میتھڈ سوال 515/516	KM کلومیٹر [ ][ ]<br>DON'T KNOW .... 88<br>REFUSED انکار کردیا 99	
558	How many minutes did it take for you to travel to this (SERVICE PROVIDER) to receive [THE LAST/CURRENT METHOD]?<br>آپ کو (سروس مہیا کرنے والے کے پاس پہنچنے میں کتنے منٹ لگ جاتے ہیں (زیر استعمال گزشتہ/موجودہ طریقہ کار)<br>[Only for methods 1-11 in Question 545/555]<br>صرف 1-11 تک کے طریقے جو سوال 545/555میں درج ہیں	MINUTES منٹس [ ][ ][ ]<br>REFUSED انکار کردیا 99<br>DON'T KNOW معلوم نہیں 88	
559	What mode(s) of transportation did you use to travel to this (SERVICE PROVIDER) to receive [THE LAST/CURRENT METHOD]?<br>گزشتہ/موجودہ طریقے کو حاصل کرنے کے لیے سروس (نام) دینے والے کے پاس جانے کے لیے آپ نے سفر کی کونسی سہولیات استعمال کی تھیں؟<br>[Only for methods 1-11 in Question 545/555]<br>صرف 1-11 تک کے طریقے جو سوال 545/555میں درج ہیں	NONE (RECEIVED AT HOME) (گھر پرکوئی نہیں ملتا) 1<br>WALK پیدل 2<br>BICYCLE بائیسکل 3<br>MOTORCYCLE موٹر سائیکل 4<br>BUS بس 5<br>CAR / TAXI کار/ٹیکسی 6<br>OTHER دیگر (وضاحت کریں) 96<br>REFUSED انکار کردیا 99	
560	How much, in Pakistani Rupee, did you have to pay in total to obtain [THE LAST/CURRENT METHOD]?<br>گزشتہ یا موجودہ طریقہ کار کی سہولیات حاصل کرنے کے لیے آپ نے کتنی رقم خرچ کی؟<br>IF NONE ENTER '00'<br>اگر کچھ نہیں تو "00" لکھیں<br>IF REFUSED ENTER '99'<br>اگر انکار کیا تو "99" لکھیں<br>[Only for methods 1-11 in Question 545/555]<br>صرف 1-11 تک کے طریقے جو سوال 544-554 میں درج ہیں	COST OF METHOD طریقہ کار کی لاگت [ ][ ][ ][ ]<br>COST OF SERVICE دورے کی لاگت [ ][ ][ ][ ]<br>TRANSPORTATION سفر [ ][ ][ ][ ]<br>LOST WAGES اجرت [ ][ ][ ][ ]<br>CHILDCARE بچے کی دیکھ بھال [ ][ ][ ][ ]	
561	How many minutes did you have to wait at the (SERVICE PROVIDER) before you received [THE LAST/CURRENT METHOD]?<br>سروس (نام) مہیا کرنے والے کے پاس گزشتہ یا موجودہ طریقے کو حاصل کرنے کے لئے آپکو کتنے منٹس تک انتظار کرنا پڑا؟<br>IF REFUSED ENTER '9999' اگر انکار کرے تو '9999'لکھیں<br>[Only for methods 1-11 in Question 545/555]<br>صرف 1-11 تک کے طریقے جو 545-555 میں درج ہیں	MINUTES منٹس [ ][ ][ ]	
562	Did you feel that you got enough information about [THE LAST/CURRENT METHOD]?<br>آپ کے خیال میں آپکو گزشتہ /موجودہ طریقہ کار طریقوں کے بارے میں کافی معلومات دی گئی ہیں؟<br>[Only for methods 1-11 in Question 545/555]<br>صرف 1-11 تک کے طریقے جو 545-555 میں درج ہیں	YES ہاں 1<br>NO نہیں 2<br>DON'T KNOW معلوم نہیں 88<br>REFUSED انکار کردیا 99	
563	Did you receive any counseling or information from a health or family planning worker about [THE LAST/CURRENT METHOD]?<br>کیا آپ نے صحت یا خاندانی منصوبہ بندی کے سہولت کار سے گزشتہ /موجودہ طریقوں کے بارے میں مشاورت یا معلومات حاصل کی ؟<br>[Only for methods 1-11 in Question 545/555]<br>صرف 1-11 تک کے طریقے جو 545-555 میں درج ہیں	YES ہاں 1<br>NO نہیں 2<br>DON'T KNOW معلوم نہیں 88<br>REFUSED انکار کردیا 99	 → 572
564	Did the person who counseled you explain how [THE LAST/CURRENT METHOD] works to prevent pregnancy?<br>کیاانہوں نے آپ سے (گزشتہ / موجود ہ طریقوں) کے بارے میں مشاورت کی تھی کہ وہ حمل سے بچانوکے لیے کیسے کام کرتے ہیں؟<br>[Only for methods 1-11 in Question 545/555]<br>صرف 1-11 تک کے طریقے جو 545-555 میں درج ہیں	YES ہاں 1<br>NO نہیں 2<br>DON'T KNOW معلوم نہیں 88<br>REFUSED انکار کردیا 99	

5. FAMILY PLANNING<br>5. خاندانی منصوبہ بندی			
NO.	QUESTIONS AND FILTERS	CODING CATEGORIES	SKIP
565	Did the person who counseled you on [THE LAST/CURRENT METHOD] explain how to use it?<br>کیا انہوں نے آپ سے(گزشتہ /موجودہ طریقوں) کے بارے میں مشاورت کی تھی، کہ ان کو کس طرح استعمال کرنا ہے؟<br>[Only for methods 1-11 in Question 545/555]<br>صرف 11-1 تک کے طریقے جو 545-555 میں درج ہیں	YES ہاں 1<br>NO نہیں 2<br>DON'T KNOW معلوم نہیں 88<br>REFUSED انکار کردیا 99	
566	Did the person who counseled you allow you to ask questions?<br>کیا مشاورت کرنے والے نے آپ کو سوال کرنے موقع دیا تھا؟<br>[Only for methods 1-11 in Question 545/555]<br>صرف 11-1 تک کے طریقے جو 545-555 میں درج ہیں	YES ہاں 1<br>NO نہیں 2<br>DON'T KNOW معلوم نہیں 88<br>REFUSED انکار کردیا 99	
567	When you got [THE LAST/CURRENT METHOD], were you told by a health or family planning worker about other methods of family planning that you could use?<br>[Only for methods 1-10 in Question 544]<br>جب آپ نے (گزشتہ/موجودہ)طریقوں کی معلومات حاصل کی ، تو آپ کو کسی صحت کے ورکر یا خاندانی منصوبہ بندی کے کارکن نےدیگر طریقوں کے بارے میں بتایا جوکہ آپ استعمال کر سکتے ہیں؟<br>[سوالات 544 میں صرف 1-10 تک کے طریقوں کے لئے]<br>[Only for methods 1-11 in Question 545/555]<br>صرف 11-1 تک کے طریقے جو 545-555 میں درج ہیں	YES ہاں 1<br>NO نہیں 2<br>DON'T KNOW معلوم نہیں 88<br>REFUSED انکار کردیا 99	
568	When you got [THE LAST/CURRENT METHOD], were you told by a health or family planning worker about potential side effects or problems you might have with the method(s)?<br>[Only for methods 1-10 in Question 554]<br>جو نئے طریقے ہیلتھ ورکر/خاندانی منصوبہ بندی کے ورکر نے آپ کو بتائے تھے تو کیا انہوں نے اس کے ممکنہ منفی اثرات یا مشکلات کے بارے میں بھی بتایا تھا؟<br>[سوالات 554 میں صرف 1-10 تک کے طریقوں کے لئے]<br>[Only for methods 1-11 in Question 545/555]<br>صرف 11-1 تک کے طریقے جو 545-555 میں درج ہیں	YES ہاں 1<br>NO نہیں 2<br>DON'T KNOW معلوم نہیں 88<br>REFUSED انکار کردیا 99	
569	When you got [THE LAST/CURRENT METHOD], were you told what to do if you experienced side effects or problems?<br>جب آپ کو (موجودہ /گزشتہ خاندانی منصوبہ بندی کے طریقوں) کی معلومات فراہم کی گئی تھی تو کیا آپ کو یہ بتایا گیا تھا کے ممکنہ منفی اثرات یا مشکلات پیش آنے کی صورت میں آپ کو کیا کرنا ہے؟<br>[Only for methods 1-11 in Question 545/555]<br>صرف 11-1 تک کے طریقے جو 545-555 میں درج ہیں	YES ہاں 1<br>NO نہیں 2<br>DON'T KNOW معلوم نہیں 88<br>REFUSED انکار کردیا 99	
570	When you got [THE LAST/CURRENT METHOD], were you told about any benefits?<br>موجودہ طریقہ کار/گزشتہ طریقوں کے ممکنہ فوائد کے بارے میں کیا آپ کو آگاہ کیاگیا تھا؟<br>[Only for methods 1-11 in Question 545/555]<br>صرف 11-1 تک کے طریقے جو 545-555 میں درج ہیں	YES ہاں 1<br>NO نہیں 2<br>DON'T KNOW معلوم نہیں 88<br>REFUSED انکار کردیا 99	

5. FAMILY PLANNING
5. خاندانی منصوبہ بندی

NO.	QUESTIONS AND FILTERS	CODING CATEGORIES	SKIP
571	When you got [THE LAST/CURRENT METHOD], were you told of any disadvantages?<br>کیا (موجودہ/گزشتہ طریقوں) کے ممکنہ نقصانات کے بارے میں آپ کو بتایا گیا تھا؟<br><br>[Only for methods 1-11 in Question 545/555]<br>صرف 1-11 تک کے طریقے جو 555-545 میں درج ہیں	YES ہاں 1<br>NO نہیں 2<br>DON'T KNOW معلوم نہیں 88<br>REFUSED انکار کردیا 99	
576	Was the method you wanted available to you?<br>جو طریقہ کار آپ استعمال کرنا چاہتی تھیں کیا وہ طریقہ دستیاب تھا؟<br><br>[Only for methods 1-11 in Question 545/555]<br>صرف 1-11 تک کے طریقے جو 555-545 میں درج ہیں	YES ہاں 1<br>NO نہیں 2<br>DON'T KNOW معلوم نہیں 88<br>REFUSED انکار کردیا 99	1 → 585
577	What method had you wanted during that visit?<br>اس وزٹ/دورہ کے دوران آپ کو نسا طریقہ استعمال کرنا چاہتی تھیں؟<br><br>RECORD ALL MENTIONED.<br>تمام دئے گئے جوابات پر نشان لگائیں ۔	FEMALE STERILIZATION خاتون کی نس بندی 1<br>MALE STERILIZATION مرد وں کی نس بندی 2<br>IUD آئی یوڈی 3<br>INJECTABLES انجیکشن 4<br>IMPLANTS امپلانٹس 5<br>PILL گولیاں 6<br>CONDOM کونڈوم 7<br>STANDARD DAYS /CALANDER METHOD دنوں کے حساب کا طریقہ/کیلنڈر 11<br>LACTATIONAL AMEN. METHOD دودھ پلانے کے دوران 12<br>WITHDRAWAL ہٹانے والاطریقہ 14<br>OTHER ______ (SPECIFY) دیگر (وضاحت کریں) 96<br>NO METHOD USED کوئی طریقہ استعمال نہیں کیا 17<br>REFUSED انکار کردیا 99	
578	What was the **most important reason** you didn't obtain the method you wanted?<br>آپ نے منتخب کردہ طریقے کو حاصل نہ کرسکنے کی سب سے اہم وجہ کیا تھی؟	METHOD OUT OF STOCK THAT DAY اس دن وہ طریقہ سٹاک میں نہیں تھا 1<br>METHOD NOT AVAILABLE AT ALL طریقہ بلکل دستیاب نہیں ہے 2<br>PROVIDER NOT TRAINED TO PROVIDE METHOD<br>مہیا کرنے والا /والی تجربہ کار نہیں 3<br>PROVIDER RECOMMENDED ANOTHER METHOD<br>مہیا کرنے والے/والی نے کوئی اور طریقہ تجویز کیا تھا 4<br>NOT ELIGIBLE FOR METHOD اس طریقے کے اہل نہیں 5<br>DECIDED NOT TO ADOPT A METHOD<br>کوئی بھی طریقہ استعمال نہیں کرنے کا فیصلہ کیا 6<br>DECIDED TO ADOPT ANOTHER METHOD<br>دوسرا کوئی طریقہ استعمال کرنے کا فیصلہ کیا 7<br>TOO COSTLY بہت مہنگا ہے 8<br>HUSBAND REFUSED شوہر نے منع کیا 9<br>OTHER, SPECIFY دیگر وضاحت 11<br>DON'T KNOW معلوم نہیں 88<br>REFUSED انکار کردیا 99	
572	Did you feel pressured into using [THE LAST/CURRENT METHOD]?<br>موجودہ/گزشتہ طریقہ کار کے استعال سے آپ کو کسی قسم کا کوئی دباؤ محسوس ہواتھا؟	YES ہاں 1<br>NO نہیں 2<br>DON'T KNOW معلوم نہیں 88<br>REFUSED انکار کردیا 99	
573	Did you feel you could say no to [THE LAST/CURRENT METHOD]?<br>کیا آپ سمجھتی ہے کہ موجودہ /گزشتہ طریقہ کار کے استعمال کو رد کرسکتی ہیں؟	YES ہاں 1<br>NO نہیں 2<br>DON'T KNOW معلوم نہیں 88<br>REFUSED انکار کردیا 99	

5. FAMILY PLANNING

5. خاندانی منصوبہ بندی

NO.	QUESTIONS AND FILTERS	CODING CATEGORIES	SKIP
574	Has the [THE LAST/CURRENT METHOD] caused any problems?<br>کیا موجودہ /گزشتہ طریقہ کار کے استعمال سے آپ کوکوئی تکلیف یا مشکلات پیش آئی تھیں ؟	YES ہاں 1<br>NO نہیں 2<br>DON'T KNOW معلوم نہیں 88<br>REFUSED انکار کردیا 99	
575	Do you feel that you have access to a wide range of family planning methods, or only to a select few?<br>کیا آپ سمجھتی ہے کہ خاندانی منصوبہ بندی کے موجودہ /گزشتہ طریقہ کار تک آپکو کافی رسائی حاصل ہے یا صرف کچھ طریقوں تک؟	A WIDE RANGE بے شمارطریقے 1<br>A SELECT FEW چند طریقے 2<br>DON'T KNOW معلوم نہیں 88<br>REFUSED انکار کردیا 99	
579	CHECK Q. 538: سوال نمبر 538 چیک کریں:<br>NEVER USED FAMILY PLANNING ☐ خاندانی منصوبہ بندی کا کوئی طریقہ استعمال نہیں کیا ↓	EVER USED FAMILY PLANNING ☐ کبھی خاندانی منصوبہ بندی کا طریقہ استعمال کیا →	585
580	Do you feel that you have access to a wide range of family planning methods?<br>کیاآپ سمجھتی ہیں کہ آپکو خاندانی منصوبہ کے طریقوں کی وسیع حد تک رسائی حاصل ہے ؟	A WIDE RANGE وسیع حد تک 1<br>A SELECT FEW کچھ منتخب شدہ 2<br>NO ACCESS کوئی رسائی نہیں 3<br>DON'T KNOW معلوم نہیں 88<br>REFUSED انکار کردیا 99	
581	Have you ever wanted to use a method of family planning?<br>کیا آپ کبھی خاندانی منصوبہ بندی کا طریقہ استعمال کرنا چاہا ؟	YES ہاں 1<br>NO نہیں 2<br>DON'T KNOW معلوم نہیں 88<br>REFUSED انکار کردیا 99	(2, 88, 99) → 585
582	Is the method of family planning you would want to use available to you?<br>خاندانی منصوبہ بندی کا وہ طریقہ جو آپ استعمال کرنا چاہتی تھیں کیا وہ دستیاب تھا؟	YES ہاں 1<br>NO نہیں 2<br>DON'T KNOW معلوم نہیں 88<br>REFUSED انکار کردیا 99	
583	What method do you want or have you wanted most recently?<br>کونسا طریقہ آپ چاہتی ہیں یا ابھی استعمال کرنا چاہ رہی ہو؟<br><br>RECORD ALL MENTIONED.<br>تمام دئے گئے جوابات پر نشان لگائیں ۔	FEMALE STERILIZATION خاتون کی نس بندی 1<br>MALE STERILIZATION مرد وں کی نس بندی 2<br>IUD آئی-یوڈی 3<br>INJECTABLES انجیکشن 4<br>IMPLANTS امپلائنٹس 5<br>PILL گولیاں 6<br>CONDOM کونڈوم 7<br>EMERGENCY CONTRACEPTION<br>STANDARD DAYS/CALANDER METHOD دنوں کے حساب کا طریقہ/کیلنڈر 11<br>LACTATIONAL AMEN. METHOD دودھ پلانے کے دوران 12<br>WITHDRAWAL ہٹانے والاطریقہ 14<br>OTHER ______ (SPECIFY) دیگر (وضاحت کریں) 96<br>NO METHOD USED کوئی طریقہ استعمال نہیں کیا 88	

5. FAMILY PLANNING
۔5 خاندانی منصوبہ بندی

NO.	QUESTIONS AND FILTERS	CODING CATEGORIES	SKIP
584	What is the **most important reason** you have not obtained the method you wanted?<br>جو طریقہ کار آپ استعمال کرنا چاہتی ہیں وہ حاصل نہ کرنے کی سب سے اہم وجہ کیا ہے؟	METHOD OUT OF STOCK THAT DAY وہی طریقہ سٹاک نہیں تھا 1<br>METHOD NOT AVAILABLE AT ALL طریقہ بلکل دستیاب نہیں ہے 2<br>PROVIDER NOT TRAINED TO PROVIDE METHOD<br>مہیا کرنے والا /والی تجربہ کار نہیں 3<br>PROVIDER RECOMMENDED ANOTHER METHOD<br>مہیا کرنے والے/والی نے کوئی اور طریقہ تجویز کیا تھا 4<br>NOT ELIGIBLE FOR METHOD اس طریقے کے اہل نہیں 5<br>DECIDED NOT TO ADOPT A METHOD<br>کوئی بھی طریقہ استعمال نہیں کرنے کا فیصلہ کیا 6<br>DECIDED TO ADOPT ANOTHER METHOD<br>دوسرا کوئی طریقہ استعمال کرنے کا فیصلہ کیا 7<br>TOO COSTLY بہت مہنگا ہے 8<br>HUSBAND REFUSED شوہر نے منع کیا 9<br>DIFFICULT TO ACCESS HEALTH CENTER<br>ہیلتھ سینٹر تک رسائی مشکل تھی 10<br>OTHER, SPECIFY دیگر وضاحت 11<br>DON'T KNOW معلوم نہیں 88<br>REFUSED انکار کردیا 99	
585	Does your husband support you in your choices related to contraceptive use?<br>جس مانع حمل طریقہ کار کا آپ انتخاب کرتی ہیں اس کے لئے کیا آپ کے شوہر آپ کا ساتھ دیتے ہیں؟	YES ہاں 1<br>NO نہیں 2<br>DON'T KNOW معلوم نہیں 88<br>REFUSED انکار کردیا 99	
586	CHECK: چیک :<br>ANY BIRTH OR PREG. TERMINATION AFTER MONTH AND YEAR OF START OF USE OF CONTRACEPTION IN Q. 545<br>کسی بچے کی ولادت یا کسی زچگی کا ضائع ہوجانا جس کے بعد مہینے/سال کے بعد زچگی روکنے کے طریقہ کو استعمال کرنا شروع کیا ۔<br>GO BACK TO **546** PROBE AND RECORD MONTH AND YEAR AT START OF CONTINUOUS USE OF CURRENT METHOD (MUST BE AFTER LAST BIRTH OR PREGNANCY TERMINATION).<br>سوال نمبر545 پر واپس جائیں تفتیش کریں اور مہینہ سال درج کریں جب سے یہ طریقہ زیر استعمال ہے (نوٹ: یاد رہے کہ کسی بچے کی ولادت کے بعد یا کسی زچگی کے ضائع ہونے کے بعد شروع کیا ہو)	YES ہاں ☐ ↓ NO نہیں ☐ ↓	
587	CHECK Q. 546 چیک<br>JULY 2015 **(6)** OR LATER جولائی 2015 (6) یا اس کے بعد ☐ ↓<br>**C** ENTER CODE FOR METHOD USED IN MONTH OF INTERVIEW IN THE CALENDAR AND IN EACH MONTH BACK TO THE DATE STARTED USING.<br>جس مہینے میں انٹرویو کیا گیا اس مہینے میں زیرا ستعمال طریقے کو یا پھر اس سے کچھ مہینے پہلے استعمال کرنا شروع کیا کوڈ کو نشان کریں	YEAR IS 2015 **(7)** OR EARLIER سال 2015 (7) یا سے پہلے ☐ ↓<br>**C** ENTER CODE FOR METHOD USED IN MONTH OF INTERVIEW IN THE CALENDAR AND EACH MONTH BACK TO JULY 2015 (6).<br>وہ کوڈ نشان کریں جو انٹرویو کے مہینے میں زیر استعمال ہے یا پھر جولائی 2015 سے اب تک ہے	

5. FAMILY PLANNING

5. خاندانی منصوبہ بندی

NO.	QUESTIONS AND FILTERS	CODING CATEGORIES	SKIP
588	I would like to ask you some questions about the times you or your husband may have used a method to avoid getting pregnant during the last 30 months.<br>USE CALENDAR TO PROBE FOR EARLIER PERIODS OF USE AND NONUSE, STARTING WITH MOST RECENT USE, BACK TO JULY 2015. **(6)**<br>USE NAMES OF CHILDREN, DATES OF BIRTH, AND PERIODS OF PREGNANCY AS REFERENCE POINTS.<br>**C**<br>**IN COLUMN 1**, ENTER METHOD USE CODE OR '0' FOR NONUSE IN EACH BLANK MONTH.<br>ILLUSTRATIVE QUESTIONS:<br>* When was the last time you used a method? Which method was that?<br>* When did you start using that method? How long after the birth of (NAME)?<br>* How long did you use the method then?<br>**IN COLUMN 2**, ENTER CODES FOR DISCONTINUATION NEXT TO THE LAST MONTH OF USE. NUMBER OF CODES IN COLUMN 2 MUST BE SAME AS NUMBER OF INTERRUPTIONS OF METHOD USE IN COLUMN 1.<br>ASK WHY SHE STOPPED USING THE METHOD. IF A PREGNANCY FOLLOWED, ASK WHETHER SHE BECAME PREGNANT UNINTENTIONALLY WHILE USING THE METHOD OR DELIBERATELY STOPPED TO GET PREGNANT.<br>ILLUSTRATIVE QUESTIONS:<br>* Why did you stop using the (METHOD)? Did you become pregnant while using (METHOD), or did you stop to get pregnant, or did you stop for some other reason?<br>* IF DELIBERATELY STOPPED TO BECOME PREGNANT, ASK: How many months did it take you to get pregnant after you stopped using (METHOD)? AND ENTER '0' IN EACH SUCH MONTH IN COLUMN 1.<br>میں آپ سے کچھ سوالات گزشتہ 30 ماہ کے پوچھونگی جس میں آپ یا آپکا شوہر نے حمل روکنے کے لیے کوئی طریقہ استعمال کیا ہوگا۔<br>ابتدائی مدت میں استعمال اور غیر استعمال طریقوں کے لیے کلینڈر سے موازنہ کریں حالیہ اور جولائی 2015 (6)<br>سی کالم 1 میں، طریقوں کے کوڈ نشان کریں یا 0 برائے ہر وہ مہینہ جس میں کچھ استعمال نہیں کیا ۔<br>مثالی سوالات :<br>• آخری مرتبہ کب آپ نے کوئی طریقہ استعمال کیا ۔وہ کونسا طریقہ تھا؟<br>• آپ نے یہ طریقے کب استعمال کرنا شروع کیا تھا۔(نام) کی پیدائش کے کتنے عرصے بعد؟<br>• تب آپ نے یہ طریقہ کتنے عرصے استعمال کیاتھا؟<br>کالم 2 میں: یہاں پر طریقہ کو بند کرنے کے بعد اور پچھلے مہینے تک استعمال ہونے ولالے طریقے کے کوڈ پر نشان کریں،کالم 2 والے نمبر کوڈز ،کالم 1 کے کوڈز کی تشریح کرتا ہوسوال کریں گے کیوں خاتون نے استعمال روک دیا ۔اگر کسی زچگی کے بعد استعمال شروع کیا تھا تو پوچھیں کے کیا استعمال کے باوجود حمل ٹھر گیا تھا یا اپنے فیصلے پر روک دیا تھا تاکہ دوسرا حمل کرسکیں۔<br>مثالی سوالات: آپ نے یہ (نام) طریقہ کیوں استعمال کرنا روک دیا ۔ کیاآپ کے (طریقہ) استعمال کرنے کے باوجود حمل ٹھر گیا تھایا آپ نے دوسرا حمل کرنے کے لیے روک دیاتھا؟یا کوئی اور وجہ تھی ۔<br>• اگر اپنی مرضی سے روک دیا تھا حمل کے لیے تو پوچھیں کے (طریقہ) کے استعمال کو روکنے کے کتنے عرصے بعد آپ کو حمل ٹھر گیا تھا۔اور ہر مہینے کے لیے 0 کا کوڈ کالم 1 میں لکھیں۔		

6. PREGNANCY AND CHILD HEALTH

601	REVIEW THE CONTRACEPTIVE CALENDAR<br>IF THE WOMAN HAD A BIRTH IN THE PAST 30 MONTHS, ASK HER<br>THE QUESTIONS ABOUT THIS LAST BIRTH. IF NOT SKIP THIS SECTION.<br>اگر اس خاتون نے گزشتہ 30 ماہ میں کسی بچے کی ولادت کی ہے تو آخری پیدائش کے بارے میں سوالات پوچھیں؟<br>اگر کوئی نہیں تو اس سیکشن کو چھوڑ دے<br><br>ONE OR MORE BIRTHS IN THE LAST 30 MONTHS گزشتہ 30 ماہ میں ایک یا زیادہ بچوں کی پیدائش ☐ ↓<br>NO BIRTHS IN THE LAST 30 MONTHS گزشتہ 30 ماہ میں کوئی پیدائش نہیں ☐ → 701<br><br>Now I would like to ask some questions about your most recent birth.<br>اب میں آپ سے حالیہ پیدائش کے بارےمیں کچھ سوالات پوچھنا چاہونگی	
602	When you got pregnant with this last baby, did you want to get pregnant at that time?<br>جس وقت آپ کو آخری حمل ہوا تھا تب کیا آپ اس وقت حاملہ ہونے کی خواہش مند تھیں؟	YES ہاں 1 (SKIP TO 605)<br>NO نہیں 2
603	Did you want to have a baby later on, or did you not want any children?<br>کیا اپ اس وقت ایک اور بچہ پیدا کرنا چاہتی تھیں یا آپ مزید کوئی بچہ نہیں چاہتی تھیں	LATER بعد میں 1<br>NO MORE/NONE<br>مزید نہیں چاہتی/نہیں چاہتی<br>(SKIP TO 605) سوال نمبر 605 چھوڑ دیں
604	How much longer did you want to wait?<br>مزید کتنا عرصہ آپ انتظار/رکنا چاہتی تھیں؟<br><br>RECORD IN UNIT RESPONDENT USES.<br>جواب دہندہ کے جواب کو گنتی میں ریکارڈ کریں	MONTHS مہینے 1 [ ] [ ]<br>YEARS سال 2 [ ] [ ]<br>DON'T KNOW معلوم نہیں 998
605	Did you see anyone for antenatal care for this pregnancy?<br><br>اس حمل کے لیے آپ نے قبل از پیدائش دیکھ بھال کے لیے کسی سے رجوع کیا؟	YES ہاں 1<br>NO نہیں 2<br>(SKIP TO 611) سوال نمبر 611 پر جائیں
606	Whom did you see?<br>آپ نے کس سے قبل از پیدائش دیکھ بھال کے لیے کے لیے رجوع کیا تھا ؟<br>Anyone else? کوئی اور<br><br>PROBE TO IDENTIFY EACH TYPE OF PERSON AND<br><br>RECORD ALL MENTIONED.<br>تمام ممکنہ لوگوں کی نشاندہی کریں اور کوڈ پر دائرہ لگائیں	**HEALTH PERSONNEL** **ہیلتھ کے نمائندے**<br>DOCTOR ڈاکٹر 1<br>NURSE/MIDWIFE نرس/مڈوائف 2<br>OFFICER/COM. HEALTH / NURSE کمیونٹی ہیلتھ آفیسر/ نرس/لیڈی ہیلتھ ورکر 3<br>**OTHER PERSON** **دیگر نمائندے**<br>TRAD. BIRTH ATTENDANT/ TBA روایتی بچہ پیدا کروانے والی داعی ٹی بی اے 4<br>LHV/LHW صحت کےرضاکار 5<br>TRAD. HEALTH PRACTITIONER<br>TRAD. HEALTH PRACTITIONER روایتی پشہ ورطبیب 6<br>OTHER________ (SPECIFY) دیگر (وضاحت کریں) 7<br>88 DON'T KNOW معلوم نہیں 88<br>99 REFUSED انکار کر دیا 99

607	Where did you receive antenatal care for this pregnancy?<br>Anywhere else?<br>آپ نے کہاں سے دوران حمل دیکھ بھال کے لیے خدمات حاصل کیں؟<br>کہیں اور سے ؟<br><br>PROBE TO IDENTIFY THE TYPE OF SOURCE.<br>ذرائع کی نشاندہی کے لئے مزید پوچھیں۔<br><br>IF UNABLE TO DETERMINE IF PUBLIC OR PRIVATE SECTOR, WRITE THE NAME OF THE PLACE.<br>اگر سرکاری یا پرائیوٹ کی نشاندہی ممکن نہ ہو تو بتائے گئے جگے کا نام لکھ دیں؟<br>____________________<br>(NAME OF PLACE) جگہ کا نام	**HOME** **گھر**<br>RESPONDENT'S HOME جواب دہندہ کا گھر 1<br>OTHER HOME کسی اور کے گھر سے 2<br>**PUBLIC SECTOR** **پبلک /سرکاری سیکٹر**<br>GOV'T HOSPITAL سرکاری ہیلتھ/آر ایچ ایس سی/سرکاری ہیلتھ سینٹر 3<br>RHC/MCH آر ایچ سی 4<br>BHU بی ایچ یو 5<br>OTHER PUBLIC SECTOR سرکاری ہیلتھ پوسٹ 6<br>____________________<br>SPECIFY<br><br>**PRIVATE MEDICAL SECTOR** **دیگر پرائیوٹ میڈیکل**<br>PVT. HOSPITAL/ CLINIC پرائیوٹ ہسپتال /کلینک 7<br>PVT. DOCTOR پرائیویٹ ڈاکٹر 8<br>HOMEOPATH ہومیو پیتھک 9<br>DISPENSER/COMPOUNDER ڈسپنسر/کمپائوڈر 10<br>HAKIM 11<br>OTHER PRIVATE MED. SECTOR دیگر پرائیوٹ وضاحت کریں 12<br>OTHER__________ دیگر وضاحت 96<br>(SPECIFY)<br>REFUSED انکار کردیا 99
PA_6_1	Do you have a maternal health book (clinic card) for this pregnancy?<br>*If yes, ask to see it and use the book to confirm responses below.*<br>کیا آپ کے پاس دوران زچگی چیک اپ کا کارڈ ہے (کلینک ریکارڈ یا فائل) جس میں آپ کی اس زچگی کا سارا ریکارڈ موجود ہو<br>اگر ہاں ہے تو درخواست کریں کہ آپ کو دکھائیں تاکہ نیچے دئیے گئے سوالات کے لیے اس کتابچہ سے مدد لی جاسکے	YES ہاں 1<br>NO نہیں 2<br>DON'T KNOW معلوم نہیں 88<br>REFUSE انکار کردیا 99
608	How many months pregnant were you when you first received antenatal care for this pregnancy?<br>آپ کی زچگی کتنے ماہ کی تھی جب آپ نے دوران حمل دیکھ بھال کی خدمات لینا شروع کی؟	MONTHS مہینے [_ | _]<br>OBSERVED مشاہدہ کیا 1<br>REPORTED رپوٹ کیا 2<br>DON'T KNOW معلوم نہیں 88<br>REFUSED انکار کردیا 99
609	How many times did you receive antenatal care during this pregnancy (at facility or visit at home by provider)?<br>اس زچگی کے دوران کتنی بار آپ نے دیکھ بھال کے لیے وزٹ کیا یا کسی نے گھر آکر آپ کو چیک کیا؟	NUMBER OF TIMES کل تعداد [_ | _]<br>OBSERVED مشاہدہ کیا 1<br>REPORTED رپوٹ کیا 2<br>DON'T KNOW معلوم نہیں 88<br>REFUSED انکار کردیا 99
610	Did you ever receive counseling on family planning during your antenatal care?<br>کیا آپ نے کبھی قبل از پیدائش دیکھ بھال کے دوران خاندانی منصوبہ بندی پر مشاورت حاصل کی؟	YES ہاں 1<br>NO نہیں 2<br>REFUSED انکار کردیا 99

611	Did anyone assist with the delivery of this baby? Who assisted?<br>کیا اس بچے کی ولادت میں کسی نے آپ کی مدد کی ؟ کس نے کی کوئی اور ؟<br>Anyone else?<br>کسی اور نے<br>PROBE FOR THE TYPE(S) OF PERSONS(S) AND RECORD ALL MENTIONED.<br>تحقیق کریں اور تمام ممکنہ اشخاص کا اندراج کریں<br>IF RESPONDENT SAYS NO ONE ASSISTED, PROBE TO DETERMINE WHETHER ANY ADULTS WERE PRESENT AT THE DELIVERY.<br>اگر جواب دہندہ کہتی ہیں کہ کسی نے دوران ولادت مدد نہیں کی تو تحقیق کریں کہ آیا کوئی بالغ مددگار موجود تھا؟	**HEALTH PERSONNEL** **ہیلتھ کے نمائندے**<br>DOCTOR ڈاکٹر 1<br>NURSE/MIDWIFE نرس/مڈوائف 2<br>OFFICER/COM. HEALTH / NURSE کمیونٹی ہیلتھ افیسر/ نرس/لیڈی ہیلتھ ورکر 3<br>**OTHER PERSON** **دیگر نمائندے**<br>TRAD. BIRTH ATTENDANT/ TBA روایتی بچہ پیدا کروانے والی داعی ٹی بی اے 4<br>LHV/LHW صحت کے رضاکار 5<br>TRAD. HEALTH PRACTITIONER<br>TRAD. HEALTH PRACTITIONER روایتی پشہ ورطبیب 6<br>OTHER________________ (SPECIFY) دیگر (وضاحت کریں) 7<br>10 NO ONE ASSISTED کوئی نہیں 10<br>88 DON'T KNOW معلوم نہیں 88<br>99 REFUSED انکار کر دیا 99
612	Where did you give birth?<br>آپ کے بچے کی پیدائش کس جگہ ہوئی تھی؟<br>PROBE FOR THE TYPE OF SOURCE.<br>IF UNABLE TO DETERMINE IF PUBLIC OR PRIVATE SECTOR, WRITE THE NAME OF THE PLACE.<br>________________<br>(NAME OF PLACE)<br>آپکی ڈیلیوری (بچے کی ولادت) کہاں ہوئی تھی؟<br>ذرائع کی قسم کے لیے تفتیش کریں<br>اگر سرکاری یا غیر سرکاری جگے کا تعین نہ کرسکے تو بتائے گئے جگے کانام لکھیں<br>________________<br>جگہ کانام	**HOME** **گھر**<br>RESPONDENT'S HOME جواب دہندہ کا گھر 1<br>OTHER HOME کسی اور کے گھر سے 2<br>**PUBLIC SECTOR** **پبلک /سرکاری سیکٹر**<br>GOV'T HOSPITAL<br>سرکاری ہیلتھ/آر ایچ ایس سی/سرکاری ہیلتھ سینٹر 3<br>RHC/MCH آر ایچ سی 4<br>BHU بی ایچ یو 5<br>OTHER PUBLIC SECTOR سرکاری ہیلتھ پوسٹ 6<br>________________ SPECIFY<br>**PRIVATE MEDICAL SECTOR** **دیگر پرائیوٹ میڈیکل**<br>PVT. HOSPITAL/ CLINIC پرائیوٹ ہسپتال /کلینک 7<br>PVT. DOCTOR پرائیوٹ ڈاکٹر 8<br>HOMEOPATH ہومیو پیتھک 9<br>DISPENSER/COMPOUNDER ڈسپنسر/کمپائونڈر 10<br>HAKIM 11<br>OTHER PRIVATE MED. SECTOR دیگر پرائیوٹ وضاحت کریں 12<br>OTHER________________ (SPECIFY) دیگر وضاحت 96<br>REFUSED انکار کردیا 99
613	Did you have a post-natal care visit for this child at any time in the six weeks after the birth?<br>کیا آپ نے اس بچے کی پیدائش کے بعد کی دیکھ بھال کے لیے 6 ہفتوں میں کسی بھی وقت دورہ کیا؟	YES ہاں 1<br>NO نہیں 2<br>NO, BABY DIED نہیں ،بچہ مرگیا 3<br>DON'T KNOW معلوم نہیں 88<br>REFUSE انکار کردیا 99
614	Did you receive family planning counseling at any post-natal visit?<br>کیا آپ نے پیدائش کے بعد کی دیکھ بھال کے دورے کے دوران خاندانی منصوبہ کی مشاورت لی؟	YES ہاں 1<br>NO نہیں 2<br>DON'T KNOW معلوم نہیں 88<br>REFUSE انکار کردیا 99

7. MARRIAGE			
NO.	QUESTIONS AND FILTERS	CODING CATEGORIES	SKIP
PA_7_1	Besides yourself, does your husband have any other wives?<br>کیا آپ کے علاوہ آپ کے شوہر کی دوسری بیویاں بھی ہیں ؟	YES ہاں 1<br>NO نہیں 2<br>DON'T KNOW معلوم نہیں 88<br>REFUSED انکار کردیا 99	2, 88, 99 → 702
PA_7_2	Including yourself, in total, how many wives does he have?<br>آپ کو شامل کرتے ہوئے کتنی بیویاں ہیں؟	TOTAL NUMBER OF WIVES کل بیویاں [ ][ ]<br>DON'T KNOW معلوم نہیں 88<br>REFUSED انکار کردیا 99	
PA_7_3	Are you the first, second, ... wife?<br>آپ پہلی ہیں ،یا دوسری ------- بیوی ہیں ؟<br>IF REFUSED, ENTER '99'<br>اگرانکار کردیا تو 99 لکھیں۔	RANK نمبر [ ][ ]	
702	Does your husband currently live with you?<br>آپ کے شوہر ابھی آپ کے ساتھ رہتے ہیں ؟	YES ہاں 1<br>NO نہیں 2<br>REFUSED انکار کردیا 99	→ 704<br><br>→ 704
703	How long has your husband been away?<br>کتنے عرصے سے آپ کے شوہرآپ سے دور ہیں ؟<br>RECORD IN UNIT RESPONDENT USES.<br>جواب دہندہ سے معلومات لے کر ہفتوں ، مہینو اور سالو میں درج کریں	MONTHS مہینہ [ ][ ]<br>WEEKS ہفتے [ ][ ]<br>YEARS سال [ ][ ]<br>DON'T KNOW معلوم نہیں 88<br>REFUSED انکار کردیا 99	
704	Have you been married once or more than once?<br>کیا آپکی شادی ایک ہی دفعہ ہوئی ہے یا ایک سے زائد بارہوئی ہے؟	ONLY ONCE ایک بار 1<br>MORE THAN ONCE ایک سے زیادہ 2<br>REFUSED انکار کردیا 99	

NO.	QUESTIONS AND FILTERS	CODING CATEGORIES	SKIP
**SECTION 8. FERTILITY PREFERENCES & CONTRACEPTION**			
801	CHECK 545: سوال نمبر 545 چیک کریں<br>NEITHER HE OR SHE STERILIZED ☐ ↓<br>کبھی بھی میاں یا بیوی نے اسٹرلائز نہیں کروایا	HE OR SHE STERILIZED ☐<br>کبھی بھی میاں یا بیوی نے اسٹرلائز کروایاہو	→ Section 9
802	CHECK 420: سوال نمبر 420 چیک کریں<br>PREGNANT ☐ ↓<br>حمل سے ہے	NOT PREGNANT OR UNSURE ☐<br>حمل سے نہیں ہے/ حمل کا تعین نہیں	→ 804
803	Now I have some questions about the future. After the child you are expecting now, would you like to have another child, or would you prefer not have any more children?<br>اب آپ سے مستقبل کے کچھ سوالات پوچھونگی - اس بچے کی ولادت کے بعد بھی آپ دوسرا بچہ کرنا چاہیں گی یا آپ کی خواہش ہو گی کہ مزید بچے نہ ہوں ؟	HAVE ANOTHER CHILD ایک اور بچہ چاہیئے 1<br>NO MORE اور بچے نہیں چاہیئے 2<br>UNDECIDED/DON'T KNOW ابھی فیصلہ نہیں کیا /معلوم نہیں 88<br>REFUSED انکار کردیا 99	→ 805<br><br>→ 811
804	Now I have some questions about the future. Would you like to have (a/another) child, or would you prefer not to have any (more) children?<br>اب آپ سے مستقبل کے کچھ سوالات پوچھونگی - کیا آپ آئندہ ایک یا زیادہ بچہ پیدا کرنا چاہیں گی یا آپ یہ چاہیں گی کہ اور بچے نہ ہوں ؟	HAVE (A/ANOTHER) CHILD ایک اور بچہ/ اور بچے ہوں 1<br>NO MORE/NONE اور نہیں /کوئی نہیں 2<br>SAYS SHE CAN'T GET PREGNANT مجھے حمل نہیں ہو سکتا 3<br>UNDECIDED/DON'T KNOW کوئی فیصلہ نہیں کیا /معلوم نہیں '88<br>REFUSED انکار کردیا 99	 → 807<br>→ 812<br>→ 810
805	CHECK 420: سوال نمر 420 چیک کریں<br>NOT PREGNANT OR UNSURE ☐ حمل نہیں ہے /حمل کا تعین نہیں ہے ↓<br>a) How long would you like to wait from now before the birth of (a/another) child? اب سے لے کر آپ ایک اور بچہ کی پیدائش میں کتنا وقفہ چاہتی ہیں؟<br>PREGNANT ☐ حمل سے ہیں ↓<br>b) After the birth of the child you are expecting now, how long would you like to wait before the birth of another child? اس بچے کی ولادت کے بعد آپ دوسرے بچہ کی پیدائش تک کتنا وقفہ چاہتی ہیں؟	MONTHS مہینے 1 ☐☐<br>YEARS سال 2 ☐☐<br>SOON/NOW جلدی/ ابھی 93<br>SAYS SHE CAN'T GET PREGNANT حمل سے نہیں ہو سکتی 94<br>OTHER دیگر وضاحت کریں 96<br>________ (SPECIFY)<br>DON'T KNOW معلوم نہیں 98<br>REFUSED انکار کردیا 99	 <br>→ 810<br>→ 812<br>→ 810
806	CHECK 420: سوال نمر 420 چیک کریں<br>NOT PREGNANT OR UNSURE ☐ ↓<br>حمل نہیں ہے /حمل کا تعین نہیں ہے	PREGNANT ☐<br>حمل سے ہیں	→ 811
807	CHECK 544: USING A CONTRACEPTIVE METHOD? سوال نمر 544 چیک کریں مانع حمل طریقہ<br>NOT CURRENTLY USING ☐ ↓<br>ابھی استعمال نہیں کر رہی ہیں	CURRENTLY USING ☐<br>ابھی استعمال کر رہی ہیں	→ 812

SECTION 8. FERTILITY PREFERENCES & CONTRACEPTION

NO.	QUESTIONS AND FILTERS	CODING CATEGORIES	SKIP
808	CHECK 805: سوال نمر 805 چیک کریں<br><br>'24' OR MORE MONTHS INTEND TO SPACE OR '02' OR MORE YEARS ☐<br>24یااس سے زیادہ مہینے کو وقفہ سمجھے یا 02 یا اس سے زیادہ سال سمجھے۔<br><br>NOT ASKED ☐<br>نہیں پوچھیں	'00-23' MONTHS INTEND TO LIMIT OR '00-01' YEAR ☐<br>-0-23مہینے کے وقفے کا ارادہ یا 0<br>1 سال	→ 811
809	CHECK 804: سوال نمر 804 چیک کریں<br><br>WANTS TO HAVE A/ANOTHER CHILD ☐<br>ایک اور یا اس سے زیادہ بچے کرنا چاہتی ہوں<br><br>WANTS NO MORE/ NONE ☐<br>اور نہیں چاہیے /کوئی نہیں<br><br>a) You have said that you want (a/another) child, but not until later...<br>Any other reason?<br><br>b) You have said that you do not want any (more) children. Can you tell me why you are not using a method to prevent pregnancy?<br>Any other reason?<br><br>آپ نے کہا کہ (a) اب آپ جلد کوئی بچہ نہیں کرنا چاہتی تو پھر آپ حمل سے بچنے کوئی طریقہ استعمال کیوں نہیں ک[illegible]؟<br>کوئی اور وجہ<br><br>آپ نے کہا کہ آپ کوئی(b) اور بچہ نہیں کرنا چاہتی تو کیا آپ بتائیں گی کہ حمل روکنے کا کوئی طریقہ کیو ں استعمال نہیں کر رہی ہیں ؟<br>کوئی اور وجہ<br><br>RECORD ALL REASONS MENTIONED.<br>تمام وجوہات کی نشاندہی کریں ۔	**FERTILITY-RELATED REASONS** **فرٹیلٹی سے وابستہ وجوہات**<br>NOT HAVING SEX جنسی تعلق نہیں ہے 2<br>INFREQUENT SEX کبھی کبھار جنسی تعلق ہے 3<br>MENOPAUSAL/HYSTERECTOMY مینوپوزل 4<br>CAN'T GET PREGNANT حمل نہیں ہو سکتا 5<br>NOT MENSTRUATED SINCE LAST BIRTH آخری بچے کی ولادت کے بعد ماہواری نہیں ہوئی 6<br>BREASTFEEDING بچے کو اپنا دودھ پلا رہی ہوں 7<br>UP TO GOD/FATALISTIC خدا کے اوپر ہے/مہلک ہے 8<br><br>**OPPOSITION TO USE** **استعمال کے حق میں نہیں**<br>RESPONDENT OPPOSED جواب دہندہ حق میں نہیں 9<br>HUSBAND OPPOSED شوہر حق میں نہیں 10<br>OTHERS OPPOSED دیگر لوگ حق میں نہیں 11<br>RELIGIOUS PROHIBITION مذہبی طور پر منع ہے 12<br>STIGMATIZING TO USE IN COMMUNITY برادری کی روایت سے منع ہے 13<br><br>**LACK OF KNOWLEDGE** **معلومات کی کمی ہے**<br>KNOWS NO METHOD کوئی طریقہ معلوم نہیں. 14<br>KNOWS NO SOURCE کوئی ذریعہ معلوم نہیں 15<br><br>**METHOD-RELATED REASONS** **طریقوں سے وابستہ وجوہات**<br>SIDE EFFECTS/HEALTH CONCERNS منفی اثرات /صحت کے خدشات ہیں 16<br>LACK OF ACCESS/TOO FAR پہنچ میں نہیں /بہت دور ہے 17<br>METHOD COSTS TOO MUCH قیمت زیادہ ہے 18<br>VISIT TO CLINIC COSTS TOO MUCH کلینک تک پہنچنے کا خرچ زیادہ ہے 19<br>CLINIC OPERATING HOURS LIMITED کلینک کا دورانیہ کم ہے 20<br>NO DOCTOR OR PROVIDER AVAILABLE ڈاکٹر /سہولت کار مہیا نہیں 21<br>PREFERRED METHOD NOT AVAILABLE پسندیدہ طریقہ دستیاب نہیں 22<br>NO METHOD AVAILABLE کوئی طریقہ دستیاب نہیں 23<br>INCONVENIENT TO USE استعمال میں دقت ہوتی ھے 24<br>INTERFERES WITH BODY'S NORMAL PROCESSES جسم کے نارمل عوامل کو متاثر کرتا ہے 25<br>26<br><br>OTHER دیگر وضاحت 27<br>(SPECIFY)<br>DON'T KNOW معلوم نہیں 88<br>REFUSED انکار کردیا 99	

SECTION 8. FERTILITY PREFERENCES & CONTRACEPTION			
NO.	QUESTIONS AND FILTERS	CODING CATEGORIES	SKIP
810	CHECK 544: USING A CONTRACEPTIVE METHOD? سوال نمر 544 چیک کریں مانع حمل طریقہ استعمال کررہی ہے؟<br>NOT ASKED نہیں پوچھا ☐ ↓ / NO, NOT CURRENTLY USING ابھی استعمال نہیں کر رہی ہیں ☐ ↓ / YES, CURRENTLY USING ہاں ابھی استعمال کر رہیں ہیں ☐ → 812		→ 812
811	Do you think you will use a contraceptive method to delay or avoid pregnancy at any time in the future?<br>کیا آپ سمجھتی ہیں کہ آپ مستقبل میں مانع حمل طریقہ کار وقفہ کرنے یا حمل کو روکنے کے لیے استعمال کریں گی؟	YES ہاں 1<br>NO نہیں 2<br>DON'T KNOW معلوم نہیں 88<br>REFUSED انکار کردیا 99	
812	CHECK 410 & 412: سوال نمبر 410 /412 چیک کریں<br>HAS LIVING CHILDREN ☐ ↓ / NO LIVING CHILDREN ☐ ↓<br>a) If you could go back to the time you did not have any children and could choose exactly the number of children to have in your whole life, how many would that be?<br>اگر آپ سے کہیں کہ اس وقت جب آپ کے بچے نہیں تھے اور آپ سے کہا جاتا کہ آپ مستقبل میں کتنے بچے کرنا پسند کرتی تو ان کی تعداد کتنی ہوتی؟<br>b) If you could choose exactly the number of children to have in your whole life, how many would that be?<br>اگر میں کہوں کہ بچوں کی وہ تعداد بتائیں جو آپ اپنی پوری ازدواجی زندگی میں کرنا چاہتی تھیں ، تو ان کی تعداد کتنی ہوتی؟<br>PROBE FOR A NUMERIC RESPONSE.<br>نمبرز میں جواب کے لئے مزید پوچھیں۔	NONE نہیں 0<br>NUMBER نمبر ☐☐<br>DON'T KNOW معلوم نہیں 88<br>REFUSED انکار کردیا 99	NONE → 814<br>DON'T KNOW → 814<br>REFUSED → 814
813	How many of these children would you like to be boys how many would you like to be girls and for how many would it not matter if it's a boy or a girl?<br>کل تعداد میں کتنے لڑکے اور لڑکیاں کرنا چاہتی تھیں یا آپ کو اس سے کوئی فرق نہیں کہ لڑکا ہو یا لڑکی؟	BOYS لڑکے / GIRLS لڑکیاں / EITHER دونوں<br>NUMBER نمبر ☐☐ ☐☐ ☐☐<br>DON'T KNOW معلوم نہیں 88<br>REFUSED انکار کردیا 99	
814	CHECK 544: USING A CONTRACEPTIVE METHOD?<br>سوال نمر 544 چیک کریں مانع حمل طریقہ استعمال کررہی ہے؟<br>CURRENTLY USING ابھی استعمال کر رہی ہے ☐ ↓ / NOT CURRENTLY USING ابھی استعمال نہیں کر رہی ہے ☐ → 817		→ 817
815	Does your husband know that you are using a method of family planning?<br>کیا آپ کے شوہر کو معلوم ہے کہ آپ خاندانی منصوبہ بندی کے طریقے استعمال کر رہی ہیں ؟	YES ہاں 1<br>NO نہیں 2<br>DON'T KNOW معلوم نہیں 88<br>REFUSED انکار کردیا 99	NO / DON'T KNOW / REFUSED → 818

SECTION 8. FERTILITY PREFERENCES & CONTRACEPTION

NO.	QUESTIONS AND FILTERS	CODING CATEGORIES	SKIP
816	Now I have some questions about your current situation. Would you say that using contraception is mainly your decision, mainly your husband's decision, or did you both decide together?<br>کیا آپ یہ کہیں گی کہ مانع حمل طریقہ کو استعمال کرنے کا فیصلہ صرف آپ کا ہے یا آپ کے شوہر یا دونوں نے مل کر کیا ہے؟	MAINLY RESPONDENT بدبندہ 1<br>MAINLY HUSBAND شوہر 2<br>JOINT DECISION دونوں نے مل کر 3<br>OTHER دیگر وضاحت کریں 96<br>(SPECIFY)<br>DON'T KNOW معلوم نہیں 88<br>REFUSED انکار کردیا 99	→ 818
817	Now I have some questions about your current situation Would you say that not using contraception is mainly your decision, mainly your husband's decision, or did you both decide together?<br>کیا آپ یہ کہیں گی کہ مانع حمل طریقہ کو استعمال نہ کرنے کا فیصلہ صرف آپ کا ہے یا آپ کے شوہر یا دونوں نے مل کر کیا ہے۔	MAINLY RESPONDENT بدبندہ 1<br>MAINLY HUSBAND شوہر 2<br>JOINT DECISION دونوں نے مل کر 3<br>OTHER دیگر وضاحت کریں 96<br>(SPECIFY)<br>DON'T KNOW معلوم نہیں 88<br>REFUSED انکار کردیا 99	
818	Does your husband want the same number of children that you want, or does he want more or fewer than you want?<br>کیا آپ کے شوہر بھی اتنے ہی بچے کرنا چاہتے تھے جتنے آپ کرنا چاہتی تھی؟	SAME NUMBER اتنےنمبرہی 1<br>MORE CHILDREN زیادہ بچے 2<br>FEWER CHILDREN کم بچے 3<br>DON'T KNOW معلوم نہیں 88<br>REFUSED انکار کردیا 99	
819	If you were to not use/since you are not using any family planning method, how likely do you think it is that you will become pregnant during the next year?<br>چونکہ آپ کوئی خاندانی منصوبہ بندی کا طریقہ استعمال نہیں کر رہی تھی تو آپ کو کیا لگتا ہے کہ آپ اگلے سال حاملہ ہو جائیں گی ؟	LIKELY ممکن ہے 1<br>UNSURE/NEUTRAL غیر یقینی ہے/کوئی رائے نہیں 2<br>UNLIKELY ناممکن ہے 3<br>REFUSED انکار کردیا 99	
820	CHECK 544: USING A CONTRACEPTIVE METHOD?<br>سوال نمر 544 چیک کریں مانع حمل طریقہ استعمال کررہی ہے؟<br>CURRENTLY USING ☐ ابھی استعمال کر رہی ہیں ↓<br>NOT ASKED OR NOT CURRENTLY USING ☐ پوچھا نہیں/ابھی استعمال نہیں کر رہی		→ 825
821	If you were to continue to use your family planning method, how likely do you think it is that you would become pregnant?<br>اگر آپ اپنے خاندانی منصوبہ بندی کے طریقے استعمال کرتی ہیں تو کیا پھر بھی اس بات کا امکان ہے کہ آپ کبھی بھی حاملہ ہو سکتی ہیں ؟	LIKELY ممکن ہے 1<br>UNSURE/NEUTRAL غیر یقینی ہے/کوئی رائے نہیں 2<br>UNLIKELY ناممکن ہے 3<br>REFUSED انکار کردیا 99<br>DON'T KNOW معلوم نہیں 88	
822	Different methods of family planning vary in how effective or ineffective they are in preventing pregnancy. How effective do you think that your family planning method is in preventing pregnancy?<br>حمل روکنے کے کئی طریقے ہیں جو پراثر بھی ہیں اور بے اثر بھی ہیں آپ کیا سمجھتی ہیں کہ آپ کے زیر استعمال طریقہ کتنا پراثر ہے حمل روکنے میں؟<br>READ OPTIONS OUT LOUD.<br>تمام جوابات کو پڑھ کر سنائیں	EFFECTIVE بے حد پر اثر ہے 1<br>SOMEWHAT EFFECTIVE کسی حد تک پر اثر ہے 2<br>UNSURE/NEUTRAL غیر یقینی ہے/کوئی رائے نہیں 3<br>SOMEWHAT INEFFECTIVE کسی حد تک بے اثر ہے 4<br>INEFFECTIVE بے اثر ہے 5<br>REFUSED انکار کردیا 99	

SECTION 8. FERTILITY PREFERENCES & CONTRACEPTION

NO.	QUESTIONS AND FILTERS	CODING CATEGORIES	SKIP
823	Different methods of family planning vary in how convenient they are to use. How convenient is it to use your family planning method?<br>خاندانی منصوبہ بندی کے طریقے کچھ آسان اور کچھ مشکل ہیں آپ کو کیا لگتا ہے کہ آپ کا زیر استعمال طریقہ کتنا آسان ہے؟<br>READ OPTIONS OUT LOUD.<br>تمام جوابات کو پڑھ کر سنائیں	CONVENIENT آسان ہے 1<br>SOMEWHAT CONVENIENT کسی حد تک آسان ہے 2<br>UNSURE/NEUTRAL غیر یقینی ہے/کوئی رائے نہیں 3<br>SOMEWHAT INCONVENIENT کسی حد تک مشکل ہے 4<br>INCONVENIENT مشکل ہے 5<br>REFUSED انکار کردیا 99	
824	Women may experience side-effects or discomfort while using a family planning method. How often do you experience any side-effect(s) or discomfort in use of your family planning method?<br>کبھی کبھی ان طریقوں کے منفی اور کچھ تکلیف دہ اثرات ہوتے ہیں آپ کو کیا لگتا ہے کہ آپ کا زیر اثر استعمال طریقہ کتنا منفی یا تکلیف دہ ہے	NEVER کبھی نہیں 1<br>SOMETIMES کبھی کبھار 2<br>OFTEN اکثر 3<br>ALL THE TIME ہر بار/ہمیشہ 4<br>DON'T KNOW معلوم نہیں 88<br>REFUSED انکار کردیا 99	
825	CHECK 420: سوال نمبر 420 چیک کریں<br>NOT PREGNANT OR UNSURE ☐<br>حمل نہیں ہے یا حمل کا ابھی تعین نہیں ↓	PREGNANT ☐ ———————————→<br>حمل سے ہیں	→ 830
826	If you were to get pregnant within the next year, would you look forward to telling your husband?<br>اگر آپ اگلے سال حمل کرنا چاہتی ہیں تو کیا آ پ اپنے شوہر سے اس سلسلے میں بات کرنا پسند کریں گی؟	YES ہاں 1<br>NO نہیں 2<br>DON'T KNOW معلوم نہیں 88<br>REFUSED انکار کردیا 99	
827	If you were to get pregnant within the next year, would you look forward to telling your parents you were pregnant?<br>اگر آپ اگلے سال حمل کرنا چاہتی ہیں تو کیا آ پ اپنے والدین کو بتانا پسند کریں گی؟	YES ہاں 1<br>NO نہیں 2<br>PARENTS DECEASED والدین کا انتقال ہو چکا ہے 3<br>DON'T KNOW معلوم نہیں 88<br>REFUSED انکار کردیا 99	
828	If you were to get pregnant within the next year, would you be worried about how to financially support the child?<br>اگر آپ اگلے سال حمل کرنا چاہتی ہیں تو کیاآپ کو اس سلسلے میں کوئی پریشانی ہے کہ آپ اپنے بچے کے اخراجات کس طرح برداشت کریں گی ؟	YES ہاں 1<br>NO نہیں 2<br>DON'T KNOW معلوم نہیں 88<br>REFUSED انکار کردیا 99	
829	If you were to get pregnant within the next year, would you consider terminating the pregnancy?<br>اگر اگلے سال آپ حاملہ ہوجاتی ہیں تو کیا آپ سوچیں گی کہ اس حمل کو ضائع کر دیا جائے؟	YES ہاں 1<br>NO نہیں 2<br>DON'T KNOW معلوم نہیں 88<br>REFUSED انکار کردیا 99	→ Skip to Section 9

SECTION 8. FERTILITY PREFERENCES & CONTRACEPTION			
NO.	QUESTIONS AND FILTERS	CODING CATEGORIES	SKIP
830	When you got pregnant, did you look forward to telling your husband?<br>جب آپ حاملہ ہوگئی توکیا آپ اپنے شوہر کو بتایا تھا ؟	YES ہاں 1<br>NO نہیں 2<br>DON'T KNOW معلوم نہیں 88<br>REFUSED انکار کردیا 99	
831	When you got pregnant with this pregnancy, did you look forward to telling your parents you were pregnant?<br>جب آپ حاملہ گئی تھی توکیا آپ اپنے والدین کو بتایا تھا ؟	YES ہاں 1<br>NO نہیں 2<br>PARENTS DECEASED والدین کا انتقال ہو چکا ہے 3<br>DON'T KNOW معلوم نہیں 88<br>REFUSED انکار کردیا 99	
832	When you got pregnant with this pregnancy, did you worry about how to financially support the child?<br>جب آپ اس حمل سے ہوئیں تو کیا آپ اس بارے میں فکر مند تھیں کہ بچے کے اخراجات کس طرح برداشت کرسکیں گی؟	YES ہاں 1<br>NO نہیں 2<br>DON'T KNOW معلوم نہیں 88<br>REFUSED انکار کردیا 99	
833	When you got pregnant with this pregnancy, did you consider terminating the pregnancy?<br>جب آپ اس حمل سے ہوئیں تو کیا آپ کو یہ خیال آیا کہ اس حمل کو ضائع کردیا جائے؟	YES ہاں 1<br>NO نہیں 2<br>DON'T KNOW معلوم نہیں 88<br>REFUSED انکار کردیا 99	

9. HUSBAND'S BACKGROUND AND WOMEN'S STATUS			
NO.	QUESTIONS AND FILTERS	CODING CATEGORIES	SKIP
902	How old was your husband on his last birthday?<br>آپ کے شوہر کی آخری سالگرہ پر کیا عمر تھی؟	AGE IN COMPLETED YEARS<br>عمر مکمل سالوں میں [ ][ ]	
902B	Can your husband read a phrase/sentence in English?<br>کیا آپ کے شوہر انگریزی میں جملہ /جملے پڑھ سکتے ہیں	YES ہاں 1<br>NO نہیں 2<br>DON'T KNOW معلوم نہیں 88<br>REFUSED انکار کردیا 99	
903a	What is your husband's occupation?<br>That is, what kind of work does he mainly do?<br>آپ کے شوہر کا پیشہ کیا ہے یعنی وہ ذیادہ تر کس طرح کا کام کرتے ہیں؟	AGRICULTURE AND LIVESTOCK زراعت اور مویشی 1<br>DAILY WAGE LABOURER روزآنہ کی مزدوری (دیہاڑی) 2<br>CIVIL SERVANT عام کسی ادارے میں ملازم 3<br>OTHER PRIVATE SERVICE HOLDER دیگر پرائیوٹ سروس ملازمت 4<br>BUSINESS/ PETTY BUSINESS کاروبار /چھوٹا کاروبار 5<br>STUDENT طالب علم 6<br>OTHER (SPECIFY) دیگر وضاحت کریں 88<br>UNEMPLOYED بے روزگار 10<br>REFUSED انکار کردیا 99	
904	Aside from your own house chores, have you done any work in the last seven days?<br>اپنے گھر کے کاموں کے علاوہ، کیا آپ نے گزشتہ سات دنوں میں کوئی کام کیا ہے؟	YES ہاں 1<br>NO نہیں 2<br>REFUSED انکار کردیا 99	YES → 908
905	As you know, some women take up jobs for which they are paid in cash or kind. Others sell things, have a small business or work on the family farm or in the family business.<br>In the last seven days, have you done any of these things or any other work, aside from household chores?<br>آپ جانتی ہیں کہ اکثر خواتین کام کیا کرتی ہیں جس کے لیے ان کو کچھ پیسے یا کچھ بدل ملتا ہے کچھ خواتین چیزیں بنا کر فروخت کرتی ہیں،جبکہ کچھ اپنا چھوٹا سا کاروبار کرتی ہیں یا پھر خاندان کے کاروبار میں یا زمینوں پر کام کرتی ہیں آخری سات دنوں میں آپ نے ان میں سے کوئی کام کیا یا کوئی اور کام کیا ہو گھر یلو کا مو ں کے علاوہ ؟	YES ہاں 1<br>NO نہیں 2<br>REFUSED انکار کردیا 99	YES → 908
906	Although you did not work in the last seven days, do you have any job or business from which you were absent for leave, illness vacation, maternity leave, or any other such reasons?<br>اگرچہ آپ نے پچھلے سات دنوں میں کوئی کام یا کاروبار وغیرہ نہیں کیا لیکن کیا آپ کسی ملازمت پر ہیں جہاں سے آپ نے غیر حاضری کی ہو یا چھٹی پر ہوں یا پھر حمل ہونے کے بعد ملنے والی چھٹیوں پر ہوں یا کوئی بھی اور وجہ؟	YES ہاں 1<br>NO نہیں 2<br>REFUSED انکار کردیا 99	
907	Have you done any work in the last 12 months apart from house hold chores?<br>پچھلے 12 مہینوں میں آپ نے کوئی کام کیا گھر کے کاموں کے علاوہ؟	YES ہاں 1<br>NO نہیں 2<br>REFUSED انکار کردیا 99	NO → 912<br>REFUSED → 912

908	What is your occupation, that is, what kind of work do you mainly do?<br>آپ کا پیشہ کیا ہے یا آپ کس قسم کے کام زیادہ تر کرتی ہیں؟	AGRICULTURE AND LIVESTOCK<br>زراعت اور مویشی 1<br>DAILY WAGE LABOURER<br>روزانہ کی مزدوری (دیہاڑی) 2<br>CIVIL SERVANT عام کسی ادارے میں ملازم 3<br>OTHER PRIVATE SERVICE HOLDER<br>دیگر پرائیوٹ سروس ملازمت 4<br>OTHER GOVERNMENT SERVICE HOLDER<br>دیگر سرکاری ملازمت 5<br>BUSINESS/ PETTY BUSINESS<br>کاروبار / پیٹیٹی کاروبار 6<br>STUDENT طالب علم 7<br>HOUSE WIFE<br>گھریلوں خاتون 8<br>OTHER (SPECIFY) دیگر وضاحت کریں 88<br>REFUSED انکار کردیا 99	
910	Who usually decides how the money you earn will be used: mainly you, mainly your husband, or you and your husband jointly?<br>عام طور پر کون فیصلہ کرتا ہے کہ آپکی آمدن کو کس طرح استعمال کیا جائے ،آپ ،آپ کے شوہر یا دونوں مل کر؟<br>[ONLY ASK IF 908 NOT SKIPPED]<br>اگر 908 اسکپ نہیں کیا ہے تو یہ سوال کریں	RESPONDENT جواب دہندہ 1<br>HUSBAND .... شوہر 2<br>RESPONDENT AND HUSBAND JOINTLY<br>جواب دہندہ اور شوہر دونوں مل کر 3<br>OTHER ______ دیگر وضاحت کریں 6<br>(SPECIFY)<br>REFUSED انکار کردیا 99	
911	Would you say that the money that you earn is more than what your husband earns, less than what he earns, or about the same?<br>کیا آپ یہ کہیں گی کہ آپ کی آمدن آپ کے شوہر کی آمدن سے زیادہ ہے ،کم ہے یا برابر ہے؟<br>[ONLY ASK IF 908 NOT SKIPPED]<br>اگر 908 اسکپ نہیں کیا ہے تو یہ سوال کریں	MORE THAN HIM شوہر سے زیادہ ہے 1<br>LESS THAN HIM شوہر سے کم ہے 2<br>ABOUT THE SAME تقریباً برابر ہے 3<br>HUSBAND DOESN'T شوہر کی کوئی آمدن نہیں<br>BRING IN ANY MONEY کہیں سے پیسہ آتا ہے 4<br>DON'T KNOW معلوم نہیں 8<br>REFUSED انکار کردیا 99	
912	Who usually decides how your husband's earnings will be used: you, your husband, or you and your husband jointly?<br>عام طور پر کون فیصلہ کرتا ہے کہ آپ کے شوہر کی آمدن کس طرح خرچ کی جائے ۔آپ ،آپ کے شوہر یا دونوں مل کر	RESPONDENT جواب دہندہ 1<br>HUSBAND .... شوہر 2<br>RESPONDENT AND HUSBAND JOINTLY<br>جواب دہندہ اور شوہر دونوں مل کر 3<br>HUSBAND HAS NO EARNINGS<br>شوہر کی کوئی آمدن نہیں 4<br>OTHER ______ دیگر وضاحت کریں 6<br>(SPECIFY)<br>REFUSED انکار کردیا 99	
913	Do you feel comfortable expressing your opinions to your husband on working outside the home?<br>کیا آپ گھر سے باہر کام کرنے کے لئے اپنے شوہر کی رائے لیتی ہیں؟	YES ہاں 1<br>NO نہیں 2<br>DON'T KNOW معلوم نہیں 88<br>REFUSED انکار کردیا 99	

914	Do you feel comfortable expressing your opinions to your husband on going out unescorted?<br>کیا آپ گھر سے باہر اکیلے جانے کے لئے اپنے شوہر سے رائے لیتی ہیں؟	YES ہاں 1<br>NO نہیں 2<br>DON'T KNOW معلوم نہیں 88<br>REFUSED انکار کردیا 99	
915	Do you feel comfortable expressing your opinions to your husband on the education and care of your children?<br>کیا آپ اپنے شوہر کو بلا جھجھک بچوں کی تعلیم صحت یا دیگر امور پر رائے دیتی ہیں؟	YES ہاں 1<br>NO نہیں 2<br>DON'T KNOW معلوم نہیں 88<br>REFUSED انکار کردیا 99	
916	Who usually makes decisions about health care for yourself: you or your husband, you and your husband jointly, another member of your household, or someone else?<br>آپ کی صحت سے متعلق فیصلے کرنے میں کون زیادہ اثر انداز ہوتا ہے آپ،آپ کے شوہر، دونوں مل کر یا گھر کا کوئی فرد یا پھر کوئی اور؟	RESPONDENT جواب دہندہ 1<br>HUSBAND شوہر 2<br>RESPONDENT & HUSBAND JOINTLY<br>جواب دہندہ اور شوہر دونوں مل کر 3<br>OTHER MALE گھر کے دوسرے مرد 4<br>OTHER FEMALE گھر کی دوسری عورت 5<br>DON'T KNOW معلوم نہیں 88<br>REFUSED انکار کردیا 99	
917	Who usually makes decisions about making major household purchases?<br>گھر کے لیے اشیاء خریدتے وقت کون فیصلے کرتاہے؟	CODES AS ABOVE اوپر دیئے گئے کوڈ لکھیں-<br>1 2 3 4 88 99	
918	Who usually makes decisions about visits to your family or relatives?<br>عام طور پر رشتہ دار یا عزیزوں سے ملنے ان کے گھر جانے کا فیصلہ کون کرتا ہے ؟	CODES AS ABOVE اوپر دیئے گئے کوڈ لکھیں-<br>1 2 3 4 88 99	
919	Do you own this or any other house either alone or jointly with someone else?<br>آپ کا کوئی ذاتی مکان ہے یا گھر کے کسی دوسرے فرد کا یا کسی کے ساتھ مل کر لیا؟	ALONE ONLY صرف اپنا 1<br>JOINTLY ONLY صرف مشترکہ 2<br>BOTH ALONE AND JOINTLY<br>دونوں مل کر اور مشترکہ 3<br>DOES NOT OWN نہیں ہے 4<br>REFUSED انکار کردیا 99	
920	Do you own any land either alone or jointly with someone else?<br>کیا آپ کسی زمین کی مالک ہیں اکیلے یا کسی کے ساتھ مشترکہ ملکیت؟	ALONE ONLY صرف اپنا 1<br>JOINTLY ONLY صرف مشترکہ 2<br>BOTH ALONE AND JOINTLY<br>دونوں مل کر اور مشترکہ 3<br>DOES NOT OWN نہیں ہے 4<br>REFUSED انکار کردیا 99	
922	In your opinion, is a husband justified in hitting or beating his wife in any of the following situations:<br>کیاآپ کے خیال میں شوہر کا اپنی بیوی کو مارنا یا کوئی جسمانی اذیت دینا ٹھیک ہے جب وہ ایسا کرے؟<br>If she goes out without telling him? اگربتائے بغیر باہر چلی جائے؟<br>If she neglects the children? اگر وہ بچوں کو نظر انداز کرے؟<br>If she argues with him? اگر وہ شوہر سے بحث کرے؟<br>If she refuses to have sex with him? اگر وہ اپنے شوہر کو جنسی ملاپ سے منع کرے؟<br>If she burns the food? اگر اس سے کھانا جل جائے تو؟<br>If she uses a family planng method<br>اگر وہ خاندانی منصو بہ بندی کا کوئی طریقہ استعما ل کر رہی ہے	(see grid below)	

	YES	NO	DK	R
GOES OUT	1	2	8	9
NEGL. CHILDREN	1	2	8	9
ARGUES	1	2	8	9
REFUSES SEX	1	2	8	9
BURNS FOOD	1	2	8	9
use of any family planng method	1	2	8	9

No.	Question	Response codes	Skip
923	Have you heard of these illnesses?<br>کیا آپ نے ان بیماریوں کے بارے میں سننا ہے؟	YES ہاں / NO نہیں / MAY BE شائد / DK معلوم نہیں / Refuse انکار d	
	(1)HIV/AIDS ایچ-آئی-وی ایڈز	1 2 3 8 9	
	(2)Hepatitis B ہیپا ٹائٹس بی	1 2 3 8 9	
	(4)Dysentery دست	1 2 3 8 9	
	(5)Gonorrhea ماہواری کے راستہ کی سوزش اور پیشاب کی جلن	1 2 3 8 9	
	(6)Genital herpes اعضاء تناسل کا انفیکشن /چھالے درد اور خارش	1 2 3 8 9	
	(7)Genital warts داد/سخت دانہ	1 2 3 8 9	
924	Do you think that you have enough information about these types of illnesses?<br>کیا آپ کو لگتا ہے اس طرح کے بیماریوں کے بارے میں آپ کو کافی معلومات ہیں؟	YES ہاں 1<br>NO نہیں 2<br>DON'T KNOW معلوم نہیں 88<br>REFUSED انکار کردیا 99	
925	Apart from AIDS, have you heard about other infections that can be transmitted through sexual contact?<br>ایڈز کے علاوہ بھی آپ نے کبھی کسی ایسے انفیکشن کے بارے میں سننا ہے جو جنسی ملاپ کی صورت میں منتقل ہوتا ہے ؟	YES ہاں 1<br>NO نہیں 2<br>DON'T KNOW/UNSURE معلوم نہیں 88<br>REFUSED انکار کردیا 99	
926	Now I would like to ask you some questions about your health in the last 12 months. During the last 12 months, have you had a disease which you got through sexual contact?<br>پچھلے 12 مہینوں میں آپ کوکوئی ایسی بیماری یا انفیکشن ہوا جو جنسی ملاپ کے نتیجے میں منتقل ہوا ہو؟	YES ہاں 1<br>NO نہیں 2<br>DON'T KNOW/UNSURE معلوم نہیں 88<br>REFUSED انکار کردیا 99	
927	Sometimes women experience a bad-smelling abnormal genital discharge. During the last 12 months, have you had a bad-smelling abnormal genital discharge?<br>بعض اوقات خواتین کے جنسی / جینیاتی جگہ سے بدبو یا غیر معمولی پانی کا اخراج ہوتا ہے۔ کیا آپ کو پچھلے 12 مہینوں میں ایسا کچھ محسوس ہوا ؟	YES ہاں 1<br>NO نہیں 2<br>DON'T KNOW/UNSURE معلوم نہیں 88<br>REFUSED انکار کردیا 99	
928	Sometimes women have small, red or flesh colored bumps and blisters in the private area. During the last 12 months, have you ever had these?<br>بعض اوقات جینیاتی جگہوں پر زخم یا السر ہوجاتا ہے آپ کو پچھلے بارہ مہینوں میں ایسی کوئی تکلیف ہوئی؟	YES ہاں 1<br>NO نہیں 2<br>DON'T KNOW/UNSURE معلوم نہیں 88<br>REFUSED انکار کردیا 99	
929	IF YES ON ANY OF 926/927/928, ASK:<br>The last time you had that condition, did you seek any kind of advice or treatment?<br>اگر سوال 926/927/928 میں سے کسی پر ہاں کا جواب ہے تو یہ سوال کریں<br>آخری بار جب ایسے کسی مسئلے سے دو چار ہوئیں توکیا آپ نے کسی سے مشورہ لیا یا علاج کروایا تھا؟	YES ہاں 1<br>NO نہیں 2<br>DON'T KNOW/UNSURE معلوم نہیں 88<br>REFUSED انکار کردیا 99	2, 88, 99 → 1001

930	Where did you go? آپ کہاں گئیں تھیں؟<br>Any other place? کسی اور جگہ<br><br>PROBE TO IDENTIFY EACH TYPE OF SOURCE.<br><br>IF UNABLE TO DETERMINE IF PUBLIC OR PRIVATE SECTOR, WRITE THE NAME OF THE PLACE.<br><br>NAME OF PLACE(S) جگہ کا نام<br><br>اگر آپکو سمجھ نہ آئے کہ ادارہ سرکاری ہے یا پرائیوٹ تو ادارے کا نام لکھ دیں؟	PUBLIC SECTOR پبلک /سرکاری سیکٹر<br>GOVT. HOSPITAL/RHSC سرکاری ہیلتھ/آر ایچ ایس سی 11<br>RURAL HEALTH CENTER, MCH<br>دیہی ہیلتھ سینٹرMCH 12<br>MCH مدر چائلڈ ہیلتھ 13<br>FAMILY HEALTH CENTRE/FWW /فیملی ہیلتھ سینٹر 14<br>MOBILE SERVICE CAMP موبائل سروس کیمپ 15<br>LADY HEALTH WORKER لیڈی ہیلتھ ورکر 16<br>LH VISITOR لیڈی وزیٹر 17<br>BASIC HEALTH UNIT بنیادی ہیلتھ کنیر 18<br>MALE MOBILIZER مرد موبالائیزر 19<br>FWA ایف ڈبلیو اے 20<br>OTHER PUBLIC (SPECIFY) دیگر (وضاحت سرکاری) 21<br><br>PRIVATE/NGO MEDICAL SECTOR<br>یرائیوٹ /NGO / ہسیتال /کلینک<br>PRIVATE/NGO/HOSPITAL/CLINIC<br>یرائیوٹ /NGO / ہسیتال /کلینک 22<br>PHARMACY, CHEMISTS فارمیسی /کلینک 23<br>PRIVATE DOCTOR یرئیوٹ ڈاکٹر 24<br>HOMEOPATH ہومیو پیتھک 25<br>DISPENSOR/ COMPOUNDER کمپاونڈر 26<br>MOBILE SERVICE CAMP موبائل سروس کیمپ 28<br>OTHER PRIVATE MEDICAL 27<br>دیگر پرائیوٹ میڈیکل (وضاحت کریں)<br>(SPECIFY)<br>OTHER SOURCE دیگر ذرائع<br>SHOP (NOT PHARMACY/CHEMIST) 31<br>دکان (فارمیسی /کمیسٹ نہیں)<br>FRIEND/RELATIVE دوست /رشتہ دار 32<br>HAKIM حکیم 33<br>DAI, TRAD. BIRTH ATTENDANT دائی/ روایتی طریقہ 34<br>OTHER (SPECIFY) دیگر (وضاحت کریں) 96<br>DON'T KNOW معلوم نہیں 88<br>REFUSED انکار کردیا 99	

10. OTHER HEALTH ISSUES			
NO.	QUESTIONS AND FILTERS	CODING CATEGORIES	SKIP
1001	Many different factors can prevent women from getting family planning advice or treatment for themselves. When you want to get family planning advice or treatment, is each of the following a big problem or not?<br>بہت سے عوامل ایسے ہیں جو خواتین کو خاندانی منصوبہ بندی کا مشورہ یا صحت سے متعلق مشورہ یا علاج کی سہولت حاصل کرنے میں مشکلات کا سبب ہوتے ہیں کیا آپ کو ان عوامل میں سے کسی کا سامنا ہے ؟	BIG PROB-LEM بڑا مسئلہ / NOT A BIG PROB-LEM بڑا مسئلہ نہیں	
	Getting permission to go to the doctor?<br>ڈاکٹر کے پاس جانے کے لئے اجازت حاصل کرنے میں؟	PERMISSION TO GO جانے کی اجازت 1 2	
	Getting money needed for advice or treatment?<br>مشورہ یا علاج کے لئے رقم حاصل کرنے میں؟	GETTING MONEY رقم حاصل کرنا 1 2	
	The distance to the health facility?<br>مرکز صحت تک جانے کا فاصلہ؟	DISTANCE فاصلہ 1 2	
	Not wanting to go alone?<br>اکیلے نہیں جانا چاہتی ہیں؟	GO ALONE اکیلے جانا 1 2	
	Not having time because of work?<br>کام کی وجہ سے وقت نہیں ملتا؟	NO TIME DUE TO WORK کام کی وجہ سے وقت نہیں 1 2	
	No one to look after children?<br>بچوں کو دیکھنے کے لئے کوئی نہیں؟	NO CHILDCARE بچوں کی دیکھ بھال نہیں 1 2	
1002	Are you covered by any health insurance?<br>کیا آپ کو کسی انشورنس کے ادارے سے علاج کے سلسلے میں سہولت میسر یاعلاج کے اخراجات مل جا تے ہیں؟	YES ہاں 1<br>NO نہیں 2<br>REFUSED انکار کردیا 99	2 → 1005
1003	What type of health insurance are you covered by?<br>RECORD ALL MENTIONED. تمام ممکنہ جوابات پر نشان لگائیں<br>اگر ہاں، تو کس قسم کی ہیلتھ انشورنس سہولیات آپ کو حاصل ہیں؟	NATIONAL/DISTRICT HEALTH INSURANCE (NHIS) قومی/ڈسٹرکٹ ہیلتھ انشورنس 1<br>HEALTH INSURANCE THROUGH EMPLOYER ہیلتھ انشورنس جہاں کام کرتے ہیں 2<br>MUTUAL HEALTH ORGANIZATION/ COMMUNITY-BASED HEALTH INSURANCE عام ہیلتھ تنظیم/کمیونٹی سطح کی ہیلتھ انشوورنس 3<br>OTHER PRIVATELY PURCHASED COMMERCIAL HEALTH INSURANCE دیگر پرائیویٹ خریدی گئی ہیلتھ انشورنس 4<br>OTHER ________ دیگر وضاحت 96<br>(SPECIFY)<br>REFUSED :.......................... انکار کردیا 99	
PK_10_1	Can I see your insurance card or other evidence of this insurance cover?<br>کیا میں آپ کا انشورنس کارڈ دیکھ سکتی ہوں یا کوئی اور پرچہ جس سے انشورنس کے یقینی ہونےکا پتہ چل سکے۔	Yes, Card seen with current cover ہاں,کارڈ دیکھ لیا جس میں حالیہ طور پر میڈیکل انشورنس 1<br>Yes, Card seen but no current cover ہاں,کارڈ دیکھ لیا مگر اس میں حالیہ طور پر میڈیکل انشورنس کور نہیں ہے۔ 2<br>No, Card not seen نہیں – کارڈ نہیں دیکھا 3<br>REFUSED انکار کردیا 99	

1004	Does your insurance cover any of the following maternity benefits:<br>کیا آپ کی میڈیکل انشورنس زچگی یا ڈیلیوری سے متعلق کسی بھی سہولت کو پورا کرتی ہے؟<br>a) Antenatal health care? دوران حمل معائنہ<br>b) Childbirth health care in a health facility?<br>بچے کی ولادت کی سہولت کسی صحت کے ادارے میں<br>c) Postnatal health care for the mother?<br>بچے کی ولادت کے بعد ماں کو ملنے والی دیکھ بھال /معائنہ<br>d) Postnatal health care for the child?<br>نوازائیدہ کو ملنے والی دیکھ بھال /معائنہ<br>e) Cash benefits during maternity leave?<br>زچگی کے لئے لی گئی چھٹیوں کے دوران ملنی والی نقد رقم<br>f) Family planning methods or services?<br>خاندانی منصوبہ بندی کے<br>g) Abortion services? حمل ضائع کروانے کی سہولت<br>h) Other? دیگر وضاحت کریں	YES ہاں / NO نہیں / DK معلوم نہیں / RF مسترد<br>ANTENATAL 1 2 8 9<br>CHILDBIRTH 1 2 8 9<br>PNC MOTHER 1 2 8 9<br>PNC CHILD 1 2 8 9<br>CASH BENEFITS 1 2 8 9<br>FAMILY PLANNING 1 2 8 9<br>ABORTION 1 2 8 9<br>OTHER 1 2 8 9	
1005	RECORD THE TIME. وقت ریکارڈ کریں	HOUR:................گھنٹے [ ][ ]<br>MINUTES...............منٹس [ ][ ]	

FOLLOW-UP INFORMATION

FOLLOW-UP INFORMATION **دورے کی معلومات**

Thank you for this information. We are planning to conduct follow-up interviews with some women whom we are interviewing now. If you are selected for a follow-up interview, you will be contacted beforehand using the contact information that you have given us, and you will be provided with information on how to participate in the interview.

اس معلومات کے لئے شکریہ۔ ہم ایسی خواتین کے ساتھ اگلا/دوبارہ انٹرویو منعقد کرنا چاہ رہے ہیں۔ اگر آپ کو انٹرویو کے لئے منتخب کیا جاتا ہے تو، ہم آپ کو پہلے مطلع کر کے آپ کا انٹریو لیں گے اور آپ کوبتائینگے کہ کس طرح سےآپ نے انٹرویو میں شامل ہونا ہے

CONTACT 1 **رابطہ نمبر 1**

FULL NAME: ____________________ پورا نام:

RELATIONSHIP TO YOU: ____________________ آپ کے ساتھ رشتہ:

LOCATION OF HOUSE/DIRECTIONS: ____________________ گھر /رہائش کا مقام:

____

____

PHONE NUMBER: ____________________ فون نمبر:

E-MAIL ADDRESS: ____________________

CONTACT 2 **رابطہ نمبر 2**

FULL NAME: ____________________ پورا نام:

RELATIONSHIP TO YOU: ____________________ آپ کے ساتھ رشتہ:

LOCATION OF HOUSE/DIRECTIONS: گھر /رہائش کا مقام:

____

____

____

PHONE NUMBER: ____________________ فون نمبر:

E-MAIL ADDRESS: ____________________ ای میل ایڈریس:

OTHER HOUSEHOLD CONTACT: **دیگر دوسرے گھر کا رابطہ:**

What is the name and phone number of someone else within your household?

آپ کے گھر کے اندر رہنے والے کسی فرد کا نام اور فون نمبر ؟

NAME: ____________________ نام:

PHONE: ____________________ فون نمبر:

RELATIONSHIP TO YOU: ____________________ آپ کے ساتھ رشتہ:

PLANS TO MOVE: **کہیں اور منتقل ہونے کا ارادہ ہے:**

Does your family/household have any plans to move in the next two years? YES NO

کیا اگلے 2 سالوں میں آپ کی فیملی یا گھرانے کا کہیں اور منتقل ہونے کا ارادہ ہے؟ ہاں نہیں

IF YES: Where do you plan to move to? LOCATION ____________________

اگر ہاں : تو آپ کہاں منتقل ہونے کا ارادہ رکھتے مقام

____

____

INSTRUCTIONS: ہدایات:

ONLY ONE CODE SHOULD APPEAR IN ANY BOX. کسی بھی ڈبے میں صرف ایک کوڈ آنا چاہیئے

COLUMN 1 REQUIRES A CODE IN EVERY MONTH.

کالم نمبر 1 میں ہر مہینے کے سامنے جواب کے

(INFORMATION TO BE CODED FOR EACH COLUMN)

(ہر ایک کالم میں معلومات ڈالنی ضروری ہے

COLUMN 1: BIRTHS, PREGNANCIES, CONTRACEPTIVE USE**

پیدائش، حمل ، فیملی پلاننگ کے طریقوں کا استعمال

- B BIRTHS — پیدائش B
- P PREGNANCIES — حمل P
- T TERMINATIONS — اسقاط حمل T

- 0 NO METHOD — فیملی پلاننگ کا کوئی طریقہ کار نہیں 0
- 1 FEMALE STERILIZATION — خاتون کی نس بندی 1
- 2 MALE STERILIZATION — مردوں کی نس بندی 2
- 3 IUD — آئییوڈی 3
- 4 INJECTABLES . — انجیکشن 4
- 5 IMPLANTS . — امپلانٹس 5
- 6 PILL — گولیاں 6
- 7 CONDOM — کونڈوم 7
- 9 EMERGENCY CONTRACEPTION — فوری طور پر حمل ضائع کرنے والی دوا 9
- J METHOD/STANDARD DAYS — کیلنڈر کا طریقہ/دنوں کے حساب کا طریقہ/ J
- K LACTATIONAL AMENORRHEA METHOD — دودھ پلانے کے دوران K
- M WITHDRAWAL — ہٹانے والاطریقہ M
- X OTHER MODERN METHOD — دیگر جدید طریقہ کار X
- Y OTHER TRADITIONAL METHOD — دیگر قدیمی طریقہ کار Y

COLUMN2 DISCONTINUATION OF CONTRACEPTIVE USE

خاندانی منصوبہ بندی کے طریقہ کار کا استعمال ترک کرنے کی وجوہات:

- 0 INFREQUENT SEX/HUSBAND AWAY — کبھی کبھار جنسی تعلق/شوہر دور ہے 0
- 1 BECAME PREGNANT WHILE USING — استعمال کرتے ہوئے حاملہ ہوئی 1
- 2 WANTED TO BECOME PREGNANT — حاملہ ہونے کی خواہش تھی 2
- 3 HUSBAND DISAPPROVED — شوہر کی طرف سے غیر منظور 3
- 4 WANTED MORE EFFECTIVE METHOD — زیادہ مفید طریقہ چاہا 4
- 5 SIDE EFFECTS/HEALTH CONCERNS — مختلف اثرات / صحت کے لئے نقصاندہ 5
- 6 LACK OF ACCESS/TOO FAR — رسائی کا مسئلہ/ تاخیر 6
- 7 COSTS TOO MUCH — اخراجات بہت زیادہ 7
- 8 INCONVENIENT TO USE — استعمال کرنے میں تکلیف ہے 8
- F UP TO GOD/FATALISTIC — خدا کے ہاتھ میں ہے F
- A DIFFICULT TO GET PREGNANT/MENOPAUSAL — حاملہ ہونا مشکل ہے/ماہواری نہیں ہوتی A
- D MARITAL DISSOLUTION/SEPARATION — ازدواجی تحلیل/علیحدگی D
- X OTHER (SPECIFY) — دیگر وضاحت X
- Z DON'T KNOW — معلوم نہیں Z

* Year of fieldwork is assumed to be 2018. For fieldwork beginning in 2019 or 2020 the years should be adjusted.

فیلڈ ورک کا سال 2018 کا فرض کیا جاتا ہے. 2020 یا 2019 میں شروع ہونے والے فیلڈ ورک کے لئے سال کو ایڈجسٹ کیا جانا چاہئے.

** Response categories may be added for other methods, including fertility awareness methods.

دیگر طریقوں کے لئے جوابی زمرہ جات شامل کیے جا سکتے ہیں.

	MONTH		COLUMN 1	COLUMN 2	
	12 DEC	1			
	11 NOV	2			
	10 OCT	3			
	9 SEP	4			
2	8 AUG	5			2
0	7 JUL	6			0
1	6 JUN	7			1
8	5 MAY	8			8
*	4 APR	9			*
	3 MAR	10			
	2 FEB	11			
	1 JAN	12			
	12 DEC	13			
	11 NOV	14			
	10 OCT	15			
	9 SEP	16			
2	8 AUG	17			2
0	7 JUL	18			0
1	6 JUN	19			1
7	5 MAY	20			7
*	4 APR	21			*
	3 MAR	22			
	2 FEB	23			
	1 JAN	24			
	12 DEC	25			
	11 NOV	26			
	10 OCT	27			
	9 SEP	28			
2	8 AUG	29			2
0	7 JUL	30			0
1	6 JUN	31			1
6	5 MAY	32			6
*	4 APR	33			*
	3 MAR	34			
	2 FEB	35			
	1 JAN	36			
	12 DEC	37			
	11 NOV	38			
	10 OCT	39			
	9 SEP	40			
2	8 AUG	41			2
0	7 JUL	42			0
1	6 JUN	43			1
5	5 MAY	44			5
*	4 APR	45			*
	3 MAR	46			
	2 FEB	47			
	1 JAN	48			

END OF THE SURVEY:

You have now reached the end of the interview. Thank you for your time and responses. This has been extremely helpful. As I said in the beginning, the purpose of this discussion was to help us learn about women's health in **Pakistan.** I also want to remind you that all of your responses will remain confidential.

آپ اب انٹرویو کے اختتام پر پہنچ گئے ہیں. آپ کے وقت اور جوابات کے لئے شکریہ. یہ بہت مددگار ثابت ہوا ہے. جیسا کہ میں نے شروع میں کہا، اس بحث کا مقصد پاکستان میں خواتین کی صحت کے بارے میں سیکھنے میں ہماری مدد کرنا تھا. میں بھی آپ کو یاد دلانا چاہتا ہوں کہ آپ کے تمام جوابات محرم رہیں گے

Do you have any further questions for me at this time? **کیا آپ اس وقت کوئی سوال پوچھنا چاہنگی؟**

Thank you again for your help **آپ کی مدد کے لئے دوبارہ آپ کا شکریہ .**

SIGNATURE OF INTERVIEWER:________________________ DATE:________________

انٹرویو لینے والے کے دستخط تاریخ

END. GO TO INTERVIEWER OBSERVATIONS.

اختتام اور انٹرویو لینے والے کے مشاہدات درج کریں

INTERVIEWER'S OBSERVATIONS

TO BE FILLED IN AFTER COMPLETING INTERVIEW

COMMENTS ABOUT INTERVIEW:

COMMENTS ON SPECIFIC QUESTIONS:

ANY OTHER COMMENTS:

EDITOR'S OBSERVATIONS
