# Supplementary material for: Conducting household surveys on reproductive health in urban settings: lessons from Karachi, Pakistan
Source: BMC Med Res Methodol. 2021 Feb 18;21:38. doi: 10.1186/s12874-021-01216-x (PMC7890958; doi:10.1186/s12874-021-01216-x)
Supplement: Supplementary file 2 — Additional file 2:. Retrospective Survey Questionnaire. [file 12874_2021_1216_MOESM2_ESM.pdf]

WILLOWS STUDY - PAKISTAN
WOMEN'S RETROSPECTIVE SURVEY, VERSION 2.0

PAKISTAN
Principal Investigator: Dr Sajid Soofi, The Aga Khan University, Pakistan

INTRODUCTION AND ELIGIBILITY

INTRODUCTION
Hello. My name is ________________ and I am a research assistant from Aga Khan University in Karachi. Along with Harvard University in the United States, I'm conducting a survey to learn about women's health in Pakistan and I would like to invite you to participate. Before we proceed, I would like to confirm your eligibility to be in the study by asking a few questions.

CO1	Do you consent to participate in this study?	YES .... 1<br>NO 2	 →END

CONSENT FORM SIGNATURE

				2	0	1	
DAY		MONTH		YEAR			

ENUMERATOR SIGNATURE
SIGNATURE OF ENUMERATOR CONFIRMS THAT INFORMED CONSENT WAS PROVIDED.

RESPONDENT IDENTIFICATION

NAME AND NUMBER OF DISTRICT ______________________ [ ][ ]

TEHSIL ______________________ [ ][ ]

TOWN/AREA ______________________ [ ][ ]

ENUMERATION AREA NUMBER . . . . [ ][ ][ ][ ][ ][ ][ ][ ][ ][ ]

CLUSTER NUMBER . . . . [ ][ ]

HOUSEHOLD ID NUMBER . . . . [ ][ ]

HOUSEHOLD LOCATION/DIRECTIONS ______________________

____

____

____

DESCRIPTION OF HOUSE/ LANDMARKS ______________________

____

GPS COORDINATES ____________ Latitude ____________ Longitude

RESPONDENT ID NUMBER [ ][ ][ ][ ][ ][ ][ ][ ][ ]

RESPONDENT CONTACT INFORMATION

FULL NAME (INCLUDING COMMON NAME) ______________________

____

PRIMARY PHONE NO. OF WOMAN ______________________

ALTERNATE PHONE NO. OF WOMAN ______________________

E-MAIL OF WOMAN ______________________

FULL NAME (INCLUDE COMMON NAME) OF HUSBAND ______________________

____

PHONE NO. OF HUSBAND ______________________

INTERVIEWER CONTACTS/VISITS

	1	2	3	FINAL CONTACT
DATE	________	________	________	DAY [ ][ ]<br>MONTH [ ][ ]<br>YEAR [ ][ ][ ][ ]
INTERVIEWER'S NAME	________	________	________	INT. NO. [ ][ ][ ]
RESULT*	________	________	________	RESULT* [ ]
NEXT CONTACT: DATE	________	________		TOTAL NUMBER [ ]

TIME	___________	___________		OF CONTACTS	

LANGUAGE OF QUESTIONNAIRE*	LANGUAGE OF INTERVIEW**	NATIVE LANGUAGE OF RESPONDENT**

** URDU=01 PUNJABI=02 SINDHI=03 PUSHTO=04 BALUCHI=05 ENGLISH=06 BARUHI=07 SARAIKI=08 09= OTHERS SPECIFY_______________

	PLEASE ENTER THE LOCATION OF INTERVIEW	HOME .... 1<br>OTHER 2	

INTRODUCTION

In this survey I will ask you questions about your health and well-being. The questions in this survey usually take about 90 minutes. All of the answers you give will be confidential and will not be shared with anyone other than members of our research team. You don't have to be in the survey, but we hope you will agree to answer the questions since your views are important. If I ask you any question you don't want to answer, just let me know and I will go on to the next question or you can stop the interview at any time.

NO.	QUESTIONS AND FILTERS	CODING CATEGORIES	SKIP
001	RECORD THE DATE OF THE INTERVIEW.	DAY . . . . [ ][ ]<br>MONTH . . . . [ ][ ]<br>YEAR . . . . [ ][ ][ ][ ]	
002	RECORD THE TIME INTERVIEW BEGINS.	HOURS . . . . [ ][ ]<br>MINUTES . . . . [ ][ ]	

1. HOUSEHOLD SCHEDULE

						IF AGE 15 OR OLDER	IF AGE 5 YEARS OR OLDER		IF AGE 5- 30YEARS AND 103=1		IF AGE 0-4 YEARS
LINE NO.	USUAL RESIDENTS NAMES	RELATIONSHIP TO WOMAN / RESPONDENT	SEX	YEAR OF BIRTH		MARITAL STATUS	EVER ATTENDED SCHOOL		CURRENT/RECENT SCHOOL ATTENDANCE		BIRTH REGISTRATION
101	102	104	105	106	108	110	111	112	113	114	115
	A. Please give me the names of the persons who usually live in your household, starting with the head of the household.<br><br>AFTER LISTING THE NAMES AND RECORDING THE RELATIONSHIP (104) AND SEX (105) FOR EACH CURRENT MEMBER OF THE HOUSEHOLD, ASK QUESTIONS 113A.	What is the relationship of (NAME) to you?<br><br>SEE CODES BELOW<br><br>PROMPT: "What do you call them? She/he is my..."	Is (NAME) male or female?	In what year was (NAME) born?	How old was (NAME) on his/her last birthday?<br><br>IF 107=2 THEN ASK: How old would (NAME) be today if he/she were	What is (NAME)'s current marital status?<br><br>1 = MARRIED<br><br>2 = DIVORCED/ SEPARATED<br>3 = WIDOWED<br>4 = NEVER-MARRIED<br><br>8 = DON'T KNOW<br>9 = REFUSED	Has /did (NAME) ever attended school?	What is the highest grade level of school (NAME) has attended?<br><br>SEE CODES BELOW.	Did (NAME) attend school at any time during the (2016-2017) school year?	During this/that school year, what grade level [is/was] (NAME) attending?<br><br>SEE CODES BELOW.	Does (NAME) have a birth certificate?<br><br>IF NO, PROBE: Has (NAME)'s birth ever been registered with the civil authority?<br><br>1 = HAS CERTIFICATE (OBSERVED)<br>2 = HAS CERTIFICATE (NOT OBSERVED)<br>3 = REGISTERED<br>4 = NEITHER<br>8 = DON'T KNOW
			M F	YEAR			Y N	LEVEL GRADE	Y N	LEVEL GRADE	
01			1 2				1 2 → NEXT LINE		1 2 → NEXT LINE		
02			1 2				1 2 → NEXT LINE		1 2 → NEXT LINE		
03			1 2				1 2 → NEXT LINE		1 2 → NEXT LINE		
04			1 2				1 2 → NEXT LINE		1 2 → NEXT LINE		
05			1 2				1 2 → NEXT LINE		1 2 → NEXT LINE		
06			1 2				1 2 → NEXT LINE		1 2 → NEXT LINE		
07			1 2				1 2 → NEXT LINE		1 2 → NEXT LINE		
08			1 2				1 2 → NEXT LINE		1 2 → NEXT LINE		
09			1 2				1 2		1 2		

							NEXT LINE		NEXT LINE		
10			1 2				1 2 NEXT LINE		1 2 NEXT LINE		
			M F	YEAR			Y N	LEVELGRADE	Y N	LEVEL GRADE	
11			1 2				1 2 NEXT LINE		1 2 NEXT LINE		
12			1 2				1 2 NEXT LINE		1 2 NEXT LINE		
13			1 2				1 2 NEXT LINE		1 2 NEXT LINE		
14			1 2				1 2 NEXT LINE		1 2 NEXT LINE		
15			1 2				1 2 NEXT LINE		1 2 NEXT LINE		
16			1 2				1 2 NEXT LINE		1 2 NEXT LINE		
17			1 2				1 2 NEXT LINE		1 2 NEXT LINE		
18			1 2				1 2 NEXT LINE		1 2 NEXT LINE		
19			1 2				1 2 NEXT LINE		1 2 NEXT LINE		
20			1 2				1 2 NEXT LINE		1 2 NEXT LINE		

CODES FOR Q. 103: RELATIONSHIP TO YOU

01 = SELF
02 = HUSBAND
03 = SON OR DAUGHTER
04 = SON-IN-LAW OR DAUGHTER-IN-LAW
05 = GRANDCHILD
06 = PARENT
07 = PARENT-IN-LAW
08 = BROTHER OR SISTER
09 = OTHER RELATIVE
10 = ADOPTED/FOSTER/ STEPCHILD
11 = NOT RELATED
12 = GRANDPARENT
13 = BROTHER-IN-LAW / SISTER-IN-LAW
14 = NEICE/NEPHEW
15 = AUNT/UNCLE
16 = COUSIN

GRADE
00 = LESS THAN 1 YEAR COMPLETEI
01 = CLASS 1
02 = CLASS 2
03 = CLASS 3
04 = CLASS 4
05 = CLASS 5
06 = CLASS 6
07 = CLASS 7
08 = CLASS 8
09 = CLASS 9
10 = MATRIC, CLASS 10
11 = CLASS 11
12 = CLASS 12
16 = MASTER'S DEGREE OR MBBS,PhD, MPHIL, BSc (4 YEARS)
88 = Don't know
99 = Refused

113A) Just to make sure that I have a complete listing: are there any other persons such as small children or infants that we have not listed? → ADD TO TABLE

2. HOUSEHOLD CHARACTERISTICS

NO.	QUESTIONS AND FILTERS	CODING CATEGORIES	SKIP
205	Does your household have:	YES NO	
	Electricity?	ELECTRICITY 1 2	
	A radio?	RADIO 1 2	
	A television?	TELEVISION 1 2	
	A cellular phone?	CELL PHONE 1 2	
	HOW MANY CELL PHONES?	NUMBER OF CELL PHONES [ ][ ]	
	A telephone (landline)?	TELEPHONE (LANDLINE) 1 2	
	A refrigerator?	REFRIGERATOR 1 2	
	Almirah/Cabinet?	ALMIRAH/CABINET 1 2	
	Room cooler?	ROOM COOLER 1 2	
	Air Conditioner?	AIR CONDITIONER 1 2	
	Chairs?	CHAIRS 1 2	
	A washing machine?	WASHING MACHINE 1 2	
	Water pump?	WATER PUMP 1 2	
	Bed?	BED 1 2	
	A wall clock?	WALL CLOCK 1 2	
	Sofa?	SOFA 1 2	
	Camera?	CAMERA 1 2	
	Sewing machine?	SEWING MACHIN 1 2	
	Computer?	COMPUTER 1 2	
	Internet connection?	INTERNET CONNECTION 1 2	
206	What type of fuel does your household mainly use for cooking?	ELECTRICITY OR GAS.................................... 1	
		WOOD, CROP RESIDUE, SAWDUST, OR ANIMAL ' 2	
		KEROSENE OR CHARCOAL 3	
		NO FOOD COOKED IN HOUSEHOLD 95	→ ##
		OTHER ____________ (SPECIFY) 96	
		REFUSED... 99	
		REFUSED 99	
207	Is the cooking usually done in the house, in a separate building, or outdoors?	IN THE HOUSE 1	
		IN A SEPARATE BUILDING 2	→ 209
		OUTDOORS 3	→ 209
		OTHER ____________ (SPECIFY) 96	→ 209
		REFUSED 99	
208	Do you have a separate room which is used as a kitchen?	YES.................................... 1	
		NO 2	
		REFUSED 99	
212	How many rooms in this household are used for sleeping?	ROOMS .... [ ][ ]	
		REFUSED 99	
213	Does any member of this household own:	YES NO	
	A watch?	WATCH	
	A bicycle?	BICYCLE 1 2	
	A motorcycle or motor scooter?	MOTORCYCLE/SCOOTER 1 2	
	A motorking?	MOTORKING	
	An animal-drawn cart (oxcart)?	ANIMAL-DRAWN CART	
	A car or truck?	CAR/TRUCK 1 2	
	A boat with a motor?	BOAT WITH A MOTOR 1 2	
	A boat without a motor?	BOAT WITHOUT A MOTOR 1 2	
217	Does someone in your household own your home?	YES 1	
		NO 2	
		DON'T KNOW 88	
		REFUSED 99	

3. RESPONDENT'S BACKGROUND

NO.	QUESTIONS AND FILTERS	CODING CATEGORIES	SKIP
303	In which region or country were you born?	PUNJAB .... 1<br>SINDH .... 2<br>KHYBER PUKHTUNKHAWA .... 3<br>BALOCHISTAN .... 4<br>GILGIT-BALTISTAN .... 5<br>FATA .... 6<br>AZAD KASHMIR .... 7<br>AFGHANISTAN .... 8<br>BANGLADESH .... 9<br>INDIA .... 10<br>OTHER COUNTRY (SPECIFY) 96	
304	For how long you have you lived in this neighborhood?<br>*Enter 888 for don't know; 999 for refused*	MONTHS .... [ ][ ]<br>YEARS .... [ ][ ]<br>DON'T KNOW .... 888<br>REFUSED 999	
305	**CHECK 304:**<br>LESS THAN OR = 5 YEARS [ ] ↓	MORE THAN 5 YEARS [ ]	→307
306	In what area were you living in **January 2013?**	Korangi (Nasir colony/Chakra goth/Gulzar Colon 1<br>Other community in Korangi 2<br>Outside Korangi 3<br>Outside Karachi .... 4<br>OTHER ______ 96<br>(SPECIFY)<br>REFUSED 99	
306A	Please think back to **January 2013.** What was your marital status at this time?	MARRIED 1<br>DIVORCED/ SEPARATED 2<br>WIDOWED 3<br>NEVER-MARRIED 4<br>DON'T KNOW 8<br>REFUSED 9	
306B	What was the highest grade level of education you had completed at the start of 2013?	LESS THAN 1 YEAR COMPLET 0<br>CLASS 1 1<br>CLASS 2 2<br>CLASS 3 3<br>CLASS 4 4<br>CLASS 5 5<br>CLASS 6 6<br>CLASS 7 7<br>CLASS 8 8<br>CLASS 9 9<br>MATRIC, CLASS 10 10<br>CLASS 11 11	

3. RESPONDENT'S BACKGROUND

NO.	QUESTIONS AND FILTERS	CODING CATEGORIES	SKIP
		CLASS 12 ... 12<br>MASTER'S DEGREE OR MBBS,PhD, MPHIL, BSc (4 YEARS) ... 13<br>Don't know ... 88<br>Refused ... 99	
306C	Please think back to **January 2013** and tell me about who you lived with. List the usual residents of your household at this time:<br>MEMBER 1<br>MEMBER 2<br>MEMBER 3<br>MEMBER 4<br>MEMBER 5<br>MEMBER 6<br>MEMBER 7<br>MEMBER 8<br>MEMBER 9<br>MEMBER 10<br>MEMBER 11<br>MEMBER 12<br>MEMBER 13<br>MEMBER 14<br>MEMBER 15<br>MEMBER 16<br>MEMBER 17<br>MEMBER 18<br>MEMBER 19<br>MEMBER 20	NAME / Relationship to you / Sex / Age in 2013	
307	What is your religion?	ISLAM ... 1<br>HINDUISM ... 2<br>CHRISTIANITY ... 3<br>BUDDHISM ... 4<br>TRADITIONAL/SPIRITUALIST ... 5<br>NO RELIGION ... 6<br>OTHER ______ (SPECIFY) ... 9<br>REFUSED ... 99	1 → PK_3_2
PK_3_1	How often do you attend religious services?	MORE THAN ONCE PER WEEK ... 1<br>ONCE PER WEEK ... 2<br>NEARLY EVERY ... 3<br>2-3 TIMES PER M ... 4<br>ABOUT ONCE PER I ... 5<br>SEVERAL TIMES PER ... 6<br>ONCE OR TWICE A ... 7<br>LESS THAN ONCE / ... 8<br>NEVER ... 9<br>REFUSED ... 99	→ 308
PK_3_2	How often do you offer your prayer?<br>**ONLY ASKED IF 307 = 1**	ONCE PER DAY ... 1<br>TWICE PER DAY ... 2<br>THREE TIMES PER DAY ... 3<br>FOUR TIMES PER DAY ... 4<br>FIVE TIMES PER DAY ... 5<br>OCCASIONALLY ... 66<br>NEVER ... 77<br>REFUSED ... 99	
308	What is your tribe or ethnic group?	URDU SPEAKING ... 1<br>SINDHI ... 2<br>PUSHTON ... 3	

3. RESPONDENT'S BACKGROUND

NO.	QUESTIONS AND FILTERS	CODING CATEGORIES	SKIP
		BALUCHI........ 4<br>BARAHVI........ 5<br>SARAIKI........ 6<br>HINDKO........ 7<br>PUNJABI........ 8<br>OTHER, SPECIFY ________ 96<br>REFUSED........ 99	

SECTION 4. REPRODUCTION

NO.	QUESTIONS AND FILTERS	CODING CATEGORIES		SKIP
401	Now I will read you a list of statements, and I would like for you to tell me how many of the statements are true for you. You should not tell me which statements are true, only how many. By giving only the number of statements that are true for you, this will preserve your privacy as I will not know which statements are true for you. After I read the list, please tell me how many of these statements are true for you. First, we will complete an example:<br><br>1. I have three children.<br>2. I am 22 years old.<br>3. I have received antenatal care during a pregnancy.<br>4. I have had a tetanus toxoid vaccination.<br><br>*How many* of these statements are true for you?	NUMBER OF STATEMENTS ....<br>REFUSED	[ ]<br>99	
402	Do you have any questions about how to answer these questions?	YES ....<br>NO	1<br>2	→ ANSWER QUESTIONS
403	Now we will complete the first real list of statements about your health. I will read you the list of statements, and I would like for you to tell me how many of these statements are true for you.<br><br>1. I have heard of an illness called polio<br>2. I have received a medical injection in the past 2 years<br>3. **I have ever smoked cigarettes**<br>4. **I had malaria as a child**<br>5. I have had an induced abortion (ended a pregnancy on purpose) [randomized]<br><br>*How many* of these statements are true for you?	NUMBER OF STATEMENTS ....<br>REFUSED	[ ]<br>99	
404	Here is the next list of statements about your health. Please tell me how many of these statements are true for you.<br><br>1. I have had a cold or flu in the last year<br>2. I have heard of an illness called diabetes (high blood sugar)<br>3. **I know someone who has told me they have high blood pressure**<br>4. I have been diagnosed with cancer<br>5. I have had an induced abortion (ended a pregnancy on purpose) [randomized]<br><br>*How many* of these statements are true for you?	NUMBER OF STATEMENTS ....<br>REFUSED	[ ]<br>99	
405	How old were you when you first got married?	AGE IN COMPLETED YEARS ....<br>DON'T KNOW ....<br>REFUSED	[ ][ ]<br>88<br>99	
406	How old were you when you had sexual intercourse for the very first time?	NEVER HAD SEXUAL INTERCOURSE ....<br>AGE IN YEARS ....<br>FIRST TIME WHEN STARTED LIVING WITH (FIRST) HUSBAND ....<br>DON'T KNOW ....<br>REFUSED	0<br>[ ][ ]<br>95<br>88<br>99	→ END SURVEY
408	Now I would like to ask about all the live births you have had during your life. Have you ever given birth?	YES ....<br>NO ....<br>REFUSED	1<br>2<br>99	 → 420
409	How old were you when you gave birth for the first time?	AGE IN YEARS<br>DON'T KNOW<br>REFUSED	[ ][ ]<br>88<br>99	
410	Do you have any sons or daughters to whom you have given birth who are now living with you?	YES ....<br>NO ....<br>REFUSED	1<br>2<br>99	 → 412
411	How many sons live with you?<br><br>And how many daughters live with you?<br><br>IF NONE, RECORD '00'.	SONS AT HOME ....<br>DAUGHTERS AT HOME	[ ][ ]<br>[ ][ ]	

SECTION 4. REPRODUCTION

NO.	QUESTIONS AND FILTERS<br>IF REFUSED, RECORD '99'	CODING CATEGORIES	SKIP
412	Do you have any sons or daughters to whom you have given birth who are alive but do not live with you?	YES .... 1<br>NO .... 2<br>REFUSED 99	2, 99 → 414
413	How many sons are alive but do not live with you?<br>And how many daughters are alive but do not live with you?<br>IF NONE, RECORD '00'.<br>IF REFUSED, RECORD '99'	SONS ELSEWHERE .... [ ][ ]<br>DAUGHTERS ELSEWHERE [ ][ ]	
414	Have you ever given birth to a boy or girl who was bom alive but later died?<br>IF NO, PROBE: Any baby who cried or showed signs of life but did not survive?	YES .... 1<br>NO .... 2<br>REFUSED 99	2, 99 → 416
415	How many boys have died?<br>And how many girls have died?<br>IF NONE, RECORD '00'.<br>IF REFUSED, RECORD '99'	BOYS DEAD .... [ ][ ]<br>GIRLS DEAD .... [ ][ ]	
416	SUM ANSWERS TO 411, 413, AND 415, AND ENTER TOTAL.<br>IF NONE, RECORD '00'.<br>IF REFUSED, RECORD '99'	TOTAL BIRTHS .... [ ][ ]	
417	CHECK 416:<br>Just to make sure that I have this right: you have had in TOTAL _____ births during your life. Is that correct?<br>YES [ ] ↓ NO [ ] → 411		
418	CHECK 416:<br>ONE OR MORE BIRTHS [ ] ↓ NO BIRTHS [ ] → 420		
420	Now I would like to ask you some questions about pregnancy. Are you pregnant now?	YES .... 1<br>NO .... 2<br>DON'T KNOW .... 88<br>REFUSED 99	2, 88, 99 → if 416 >=1 go to 424<br>if 416=0 go to 425
421	How many months pregnant are you?<br>RECORD NUMBER OF COMPLETED MONTHS.	MONTHS .... [ ][ ]<br>DON'T KNOW .... 88<br>REFUSED 99	
422	When you got pregnant, did you want to get pregnant at that time?	YES .... 1<br>NO .... 2<br>DON'T KNOW .... 88<br>REFUSED 99	1 → PK_4_1
423	Did you want to have a baby later on or did you not want any more children?	LATER .... 1<br>NO MORE/NONE .... 2<br>DON'T KNOW .... 88<br>REFUSED 99	
PK_4_1	When you got pregnant, did your husband want you to get pregnant at that time?	YES .... 1<br>NO .... 2<br>DON'T KNOW .... 88<br>REFUSED 99	
424	Have you given birth within the last six months?	YES .... 1<br>NO .... 2<br>REFUSED 99	
424B	What was the date of the last time you gave birth?<br>PROBE: Date of most recent live birth	MONTH .... [ ][ ]<br>YEAR .... [ ][ ][ ][ ]<br>DON'T KNOW .... 88/8888<br>REFUSED 99/9999	
425	Have you ever had a pregnancy that miscarried, was aborted, or ended in a stillbirth?	YES .... 1<br>NO .... 2<br>REFUSED 99	2, 99 → 501

SECTION 4. REPRODUCTION

NO.	QUESTIONS AND FILTERS	CODING CATEGORIES	SKIP
426	How long ago did the last such pregnancy end?	Days ago [ ][ ]<br>Weeks ago [ ][ ]<br>Months ago [ ][ ]<br>Years ago [ ][ ]<br>DON'T KNOW 888<br>REFUSED 999	
427	How many pregnancies were miscarried, aborted, or ended in a stillbirth?	NUMBER OF PREGNANCY LOSSES [ ][ ]<br>REFUSEC 99	
428	How many pregnancies ended in a stillbirth?	NUMBER OF STILLBIRTHS .... [ ][ ]<br>NONE 00<br>DON'T KNOV .. .. 88<br>REFUSEC 99	
429	How many pregnancies were miscarried?	NUMBER OF MISCARRIAGES .... [ ][ ]<br>NONE 00<br>DON'T KNOV .. .. 88<br>REFUSEC 99	
430	How many pregnancies were terminated by induced abortion? (intentional termination of pregnancy)	NUMBER OF ABORTIONS .... [ ][ ]<br>NONE 00<br>DON'T KNOV .. .. 88<br>REFUSEC 99	00, 88, 99 → 444
431	How long ago was the last induced abortion?<br>(RECORD IN UNIT RESPONDENT USES)	[ ][ ] Days ago<br>[ ][ ] Weeks ago<br>[ ][ ] Months ago<br>[ ][ ] Years ago<br>DON'T KNOV .... 888<br>REFUSEC 999	
435	What was done to have the pregnancy terminated?	SURGERY (OPERATION) 1<br>INJECTION 2<br>TOOK PILLS/MEDICINES/MEDICATION ABORTION (M/ 3<br>INSERTED HERBS OR OBJECT IN WOMB, SPECIFY 4<br>TOOK HOMEMADE MEDICINE, SPECIFY_________ 5<br>OTHER, SPECIFY 96<br>REFUSED 99	2 → 432<br>3 → 437<br>4, 5, 96, 99 → 432
436	What was the name of the surgical procedure used to end the pregnancy?	VACUUM ASPIRATION (MVA or EVA) 1<br>DILATION AND CURETTAGE (D&C)/ SHARP CURETTAGE 2<br>IUD INSERTION 3<br>OTHER, SPECIFY 96<br>DON'T KNOW 88<br>REFUSEC 99	→ 432
437	What kind of medication did you use to end the pregnancy?<br>SHOW PHOTOGRAPH OF EACH METHOD	MISOPROSTOL ALONE (ST MOM/CYTOTEC) 2<br>ORAL CONTRACEPTIVE PILLS 3<br>OTHER PILLS 4<br>OTHER, SPECIFY 88<br>DON'T KNOW 99<br>REFUSED	
438	In total, how many tablets or pills did you take to end the pregnancy?	NUMBER OF PILLS .... [ ][ ]<br>NOT APPLICABLE 77<br>DON'T KNOW 88<br>REFUSED 99	
432	From whom/where did you receive induced abortion services the last time?<br>SELECT ALL THAT APPLY	PUBLIC SECTOR<br>GOVT. HOSPITAL/RHSC ...... 11<br>RURAL HEALTH CENTRE, MCH ...... 12<br>MCH .... 13<br>FAMILY HEALTH/WELFARE CENTF .... 14<br>MOBILE SERVICE CAMP 15	

NO.	QUESTIONS AND FILTERS	CODING CATEGORIES	SKIP
		LADY HEALTH WORKER 16<br>LADY HEALTH VISITOR 17<br>BASIC HEALTH UNIT 18<br>MALE MOBILIZER 19<br>FWA 20<br>OTHER PUBLIC ______ 21<br>(SPECIFY)<br><br>PRIVATE/NGO MEDICAL SECTOR<br>PRIVATE/NGO HOSPITAL/CLINIC .... 22<br>PHARMACY, CHEMISTS .... 23<br>PRIVATE DOCT(......................... 24<br>HOMEOPATH .... 25<br>DISPENSOR/ COMPUNCER 26<br>MOBILE SERVICE CAMP 27<br>OTHER PRIVATE<br>MEDICAL ______ 28<br>(SPECIFY)<br><br>OTHER SOURCE<br>SHOP (NOT PHARMACY/CHEMIST) 31<br>FRIEND/RELATIVE 32<br>HAKIM .... 33<br>DAI, TRAD. BIRTH ATTENDANT .... 34<br><br>OTHER ______ 96<br>(SPECIFY)<br>DON'T KNOW 88<br>REFUSED 99	
433	From whom / where did you learn about this place (primary source of information)?<br><br>Select only one (probe for primary source)	NEIGHBOR 1<br>FRIEND 2<br>MOTHER 3<br>MOTHER-IN-LAW 4<br>HUSBAND 5<br>OTHER RELATIVE 6<br>DOCTOR/PHYSICIAN 7<br>NURSE/MIDWIFE/LHV WORKING AT HEALTH FACILIT 8<br>NURSE/MIDWIFE/LHV MAKING A HOME VISIT 9<br>LHW MAKING A HOME VISIT 10<br>WILLOWS FIELD EDUCATOR MAKING A HOME VISIT 13<br>PHARMACIST 11<br>NO ONE 12<br>OTHER ______ 96<br>(SPECIFY)<br>DON'T KNOW .... 88<br>REFUSED 99	
434	Who influenced you the most when deciding on this abortion?	NEIGHBOR 1<br>FRIEND 2<br>MOTHER 3<br>MOTHER-IN-LAW 4<br>HUSBAND 5<br>OTHER RELATIVE 6<br>DOCTOR/PHYSICIAN 7<br>NURSE/MIDWIFE/LHV WORKING AT HEALTH FACILIT 8<br>NURSE/MIDWIFE/LHV MAKING A HOME VISIT 9<br>LHW MAKING A HOME VISIT 10<br>WILLOWS FIELD EDUCATOR MAKING A HOME VISIT 13<br>PHARMACIST 11<br>NO ONE 12<br>OTHER ______ 96<br>(SPECIFY)<br>DON'T KNOW .... 88<br>REFUSED 99	
440	After termination, did you experience any health problems? (Select all that apply)	HEAVY BLEEDING 1<br>VOMITING/NAUSEA 2<br>ABDOMINAL PAIN 3<br>FEVER 4<br>FOUL-SMELLING DISCHARGE/INFECTION 5<br>NO HEALTH PROBLEMS 6<br>OTHER, SPECIFY ______ 96<br>REFUSED 99	NO HEALTH PROBLEMS →501
439	After the abortion, did you seek care or treatment for any symptoms?	YES 1<br>NO 2<br>REFUSED 99	NO, REFUSED → 444
441	From whom or where did you receive treatment for your symptoms?<br><br>SELECT ALL THAT APPLY	PUBLIC SECTOR<br>GOVT. HOSPITAL/RHSC ...... 11<br>RURAL HEALTH CENTRE, MCH ...... 12<br>MCH .... 13<br>FAMILY HEALTH/WELFARE CENTF .... 14<br>MOBILE SERVICE CAMP .... 15<br>LADY HEALTH WORKER 16<br>LADY HEALTH VISITOR 17<br>BASIC HEALTH UNIT 18<br>MALE MOBILIZER 19<br>FWA 20<br>OTHER PUBLIC ______ 21<br>(SPECIFY)<br><br>PRIVATE/NGO MEDICAL SECTOR	

NO.	QUESTIONS AND FILTERS	CODING CATEGORIES		SKIP
		PRIVATE/NGO HOSPITAL/CLINIC	22	
		PHARMACY, CHEMISTS	23	
		PRIVATE DOCT(	24	
		HOMEOPATH	25	
		DISPENSOR/ COMPUNCER	26	
		MOBILE SERVICE CAMP	27	
		OTHER PRIVATE MEDICAL ________ (SPECIFY)	28	
		OTHER SOURCE		
		SHOP (NOT PHARMACY/CHEMIST)	31	
		FRIEND/RELATIVE	32	
		HAKIM	33	
		DAI, TRAD. BIRTH ATTENDANT	34	
		OTHER ________ (SPECIFY)	96	
		DON'T KNOW	88	
		REFUSED	99	
442	Who influenced you the most when deciding on this place?	NEIGHBOR	1	
		FRIEND	2	
		MOTHER	3	
		MOTHER-IN-LAW	4	
		HUSBAND	5	
		OTHER RELATIVE	6	
		DOCTOR/PHYSICIAN	7	
		NURSE/MIDWIFE/LHV WORKING AT HEALTH FACILIT	8	
		NURSE/MIDWIFE/LHV MAKING A HOME VISIT	9	
		LHW MAKING A HOME VISIT	10	
		WILLOWS FIELD EDUCATOR MAKING A HOME VISIT	13	
		PHARMACIST	11	
		NO ONE	12	
		OTHER (SPECIFY)	96	
		DON'T KNOW	88	
		REFUSED	99	
443	Did you receive any family planning counseling during this visit?	YES	1	
		NO	2	
		DON'T KNOW	88	
		REFUSED	99	
444	**C** FOR EACH PREGNANCY THAT DID NOT END IN A LIVE BIRTH IN JANUARY 2013 **(Col. 1)** OR LATER, ENTER 'T' IN THE CALENDAR IN THE MONTH THAT THE PREGNANCY TERMINATED AND 'P' FOR THE REMAINING NUMBER OF COMPLETED MONTHS OF PREGNANCY.<br><br>IF THERE ARE MORE THAN FOUR PREGNANCIES THAT DID NOT END IN A LIVE BIRTH, USE AN ADDITIONAL QUESTIONNAIRE STARTING ON THE SECOND LINE.			

5. FAMILY PLANNING

NO.	QUESTIONS AND FILTERS	CODING CATEGORIES	SKIP
501	Now I would like to talk about family planning - the various ways or methods that a man and woman can use to delay or avoid a pregnancy. Have you heard of:		
1	Female Sterilization. PROBE: Women can have an operation to avoid having any more children.	YES ..... 1<br>NO ..... 2<br>REFUSED 99	
2	Male Sterilization. PROBE: Men can have an operation to avoid having any more children.	YES ..... 1<br>NO ..... 2<br>REFUSED 99	
3	IUD. PROBE: Women can have a loop or coil placed inside them by a doctor or a nurse	YES ..... 1<br>NO ..... 2<br>REFUSED 99	
4	Injectables. PROBE: Women can have an injection by a health provider that stops them from becoming pregnant for one or more months.	YES ..... 1<br>NO ..... 2<br>REFUSED 99	
5	Implants. PROBE: Women can have one or more small rods placed in their upper arm by a doctor or nurse which can preventy pregnancy for one or more years.	YES ..... 1<br>NO ..... 2<br>REFUSED 99	
6	Pill. PROBE: Women can take a pill every day to avoid becomine pregnant.	YES ..... 1<br>NO ..... 2<br>REFUSED 99	
7	Condom. PROBE: Men can put a rubber sheath on their penis before sexual intercourse to prevent pregnancy.	YES ..... 1<br>NO ..... 2<br>REFUSED 99	
10	Lactational Amenorrhea Method (LAM). PROBE: Women can exclusively breastfeed their children until 6 months of age to avoid pregnancy.	YES ..... 1<br>NO ..... 2<br>REFUSED 99	
11	Rhythm/Calendar Method. PROBE: To avoid pregnancy, women do not have sexual intercourse on the days of the month they think they can get pregnant.	YES ..... 1<br>NO ..... 2<br>REFUSED 99	
12	Withdrawal. PROBE: Men can be careful and pull out before climax.	YES ..... 1<br>NO ..... 2<br>REFUSED 99	
13	Emergency Contraception. PROBE: As an emergency measure, within three days after they have unprotected sexual intercourse, women can take special pills to prevent pregnancy.	YES ..... 1<br>NO ..... 2<br>REFUSED 99	
14	Have you heard of any other ways or methods that women or men can use to avoid pregnancy?	YES ..... 1<br>_______________<br>(SPECIFY)<br>_______________<br>(SPECIFY)<br>NO ..... 2<br>DON'T KNOW ..... 88<br>REFUSED 99	
502	IF "NO" TO ALL OF 501, SKIP<br><br>From whom did you learn about family planning methods?	NEIGHBOR 1<br>FRIEND 2<br>RELATIVE 3<br>HUSBAND 4	

NO.	QUESTIONS AND FILTERS	CODING CATEGORIES		SKIP
	SELECT ALL THAT APPLY	DOCTOR	5	
		NURSE/MIDWIFE WORKING AT HEALTH FACILITY	6	
		NURSE/MIDWIFE MAKING A HOME VISIT	7	
		WILLOWS FIELD WORKER	8	
		OTHER FIELD WORKER MAKING A HOME VISIT	9	
		PHARMACIST	10	
		SCHOOL	11	
		NEWSPAPER OR MAGAZINE OR BOOK	12	
		TELEVISION/RADIO	13	
		INTERNET	14	
		SOCIAL MEDIA	15	
		NO ONE	16	
		BILLBOARD	17	
		OTHER ______ (SPECIFY)	96	
		DON'T KNOW	88	
		REFUSED	99	
503	According to **Pakistani** law, under what condition(s) is it legal to have an induced abortion?<br>CIRCLE ALL THOSE MENTIONED SPONTANEOUSLY BY THE RESPONDENT	On request	1	
		Economic or social reasons	2	
		Foetal impairment	3	
		Rape	4	
		Incest	5	
		Intellectual or cognitive disability of the woman	6	
		Mental Health of the mother	7	
		Physical Health of the mother	8	
		Not legal under any conditions	9	→ 505
		Other ______	96	
		Don't know	88	→ 505
		Refused	99	→ 505
504	According to **Pakistani** law, up to how many weeks of pregnancy is it legal to have an induced abortion?	WEEKS ☐☐		
		DON'T KNOW	88	
		REFUSEI	99	
505	What methods for induced abortion have you ever heard of?<br>SELECT ALL THAT APPLY.	SURGERY/OPERATION	1	
		TABLETS/PILLS/MEDICATION ABORTION (MA)	2	
		HOMEMADE CONCOCTION	3	
		INSERTING HERBS OR OBJECT IN WOMB	4	
		MASSAGE	5	
		INJECTION	7	
		NONE	6	
		OTHER ______ (SPECIFY)	96	
		REFUSED	99	
506	Have you ever been given information about having an induced abortion for an unwanted pregnancy?	YES	1	
		NO	2	→ 508
		DON'T KNOW	88	→ 508
		REFUSED	99	→ 508
507	Where did you get information about induced abortion?<br>SELECT ALL THAT APPLY	NEIGHBOR	1	
		FRIEND	2	
		MOTHER	3	
		MOTHER-IN-LAW	4	
		HUSBAND	5	
		OTHER RELATIVE	6	
		DOCTOR/PHYSICIAN	7	
		NURSE/MIDWIFE/LHV WORKING AT HEALTH FACILITY	8	
		NURSE/MIDWIFE/LHV MAKING A HOME VISIT	9	
		LHW MAKING A HOME VISIT	10	
		WILLOWS FIELD EDUCATOR MAKING A HOME VISIT	13	
		PHARMACIST	11	
		NO ONE	12	
		OTHER ______ (SPECIFY)	96	
		DON'T KNOW	88	
		REFUSEI	99	

NO.	QUESTIONS AND FILTERS	CODING CATEGORIES		SKIP
508	In the past 5 years, since 2013, has any health worker come to your home to talk with you about preventing pregnancy or terminating an unintended pregnancy?	YES .... 1<br>NO .... 2<br>DON'T KNOW .... 88<br>REFUSED 99		2, 88, 99 → 524
509	What organization(s) was/were the health worker(s) from?<br><br>SELECT ALL THAT APPLY	WILLOWS .... 1<br>GOVERNMENT .... 2<br>OTHER, SPECIFY .... 96<br>DON'T KNOW .... 88<br>REFUSED 99		
		A= WILLOWS (509 = 1)	B= ALL OTHERS (509 = 2, 96, 88, 99)	
510	A) How many times did someone come to visit you for this type of visit?<br><br>B) In the last 5 years, how many times did someone come to visit you for this type of visit?	1 TIN .... 1 (SKIP TO 512)<br>2 TIN .... 2<br>3-5 TIMI .... 3<br>MORE THAN 5 TII .... 4<br>DON'T KNOW .... 88<br>REFUSED 99	1 TIMI .... 1 (SKIP TO 512)<br>2 TIMES .... 2<br>3-5 TIME: .... 3<br>MORE THAN 5 TIMES .... 4<br>DON'T KNOW .... 88<br>REFUSEI 99	
511	Did the same person come to visit you more than once?	YES .... 1<br>NO .... 2<br>DON'T KNOW .... 88<br>REFUSED 99	YES .... 1<br>NO .... 2<br>DON'T KNOW .... 88<br>REFUSED 99	
512	A) What gender was the person(s) who came to visit?<br><br>B) What gender was the person(s) who came to the LAST visit?	MALE .... 1<br>FEMALE .... 2<br>BOTH MALE AND FEMALE VISITO 3<br>REFUSED 99	MALE .... 1<br>FEMALE .... 2<br>BOTH MALE AND FEMALE VISIT(3<br>REFUSED 99	
513	A) What topics did the person(s) talk with you about during the home visit?<br>B) What topics did the person(s) talk with you about during the LAST home visit?<br><br>SELECT ALL THAT APPLY<br><br>DO NOT READ RESPONSES ALOUD	PREGNANCY 1<br>FAMILY PLANNING 2<br>ABORTION 3<br>SEXUALLY TRANSMITTED INFECTIONS (STIs) 4<br>PAP SMEAR 5<br>BREAST EXAM 6<br>INFERTILITY 7<br>OTHER ______ (SPECIFY) 96<br>DON'T KNOW 88<br>REFUSED 99	PREGNANCY 1<br>FAMILY PLANNING 2<br>ABORTION 3<br>SEXUALLY TRANSMITTED INFECTIONS (STIs 4<br>PAP SMEAR 5<br>BREAST EXAM 6<br>INFERTILITY 7<br>OTHER ______ (SPECIFY) 96<br>DON'T KNOW 88<br>REFUSED 99	
514	Were you counseled or provided information on using family planning methods?	YES .... 1<br>NO .... 2<br>DON'T KNO\ .... 88<br>REFUSED .... 99<br>(2, 88, 99: SKIP TO 516)	YES .... 1<br>NO .... 2<br>DON'T KNOW .... 88<br>REFUSEI .... 99<br>(2, 88, 99: SKIP TO 516)	
515	Which method(s) were you counseled/given information on?<br><br>CIRCLE ALL MENTIONED.	FEMALE STERILIZATION .... 1<br>MALE STERILIZATION .... 2<br>IUD .... 3<br>INJECTABLES .... 4<br>IMPLANTS .... 5<br>PILL .... 6<br>CONDOM .... 7<br>EMERGENCY CONTRACEPTION 11<br>STANDARD DAYS/ CALENDAR MI 12<br>LACTATIONAL AM .... 13<br>WITHDRAWAL .... 15<br>OTHER MODERN METHOD 16<br>OTHER TRADITIONAL M .... 17<br>NONE .... 18<br>DON'T KNOW/REMEMBER 88<br>REFUSED 99	FEMALE STERILIZATI .... 1<br>MALE STERILIZATIOI .... 2<br>IUD .... 3<br>INJECTABLES .... 4<br>IMPLANTS .... 5<br>PILL .... 6<br>CONDOM .... 7<br>EMERGENCY CONTF .... 11<br>STANDARD DAYS/ CALENDAR M 12<br>LACTATIONAL AMEN .... 13<br>WITHDRAWAL .... 15<br>OTHER MODERN ME .... 16<br>OTHER TRADITIONAI .... 17<br>NONE .... 18<br>DON'T KNOW/REMEMBER 88<br>REFUSED 99	
516	Who else (besides yourself) participated in the information sessions?<br><br>CIRCLE ALL MENTIONED.	HUSBAND .... 1<br>MOTHER .... 2<br>MOTHER IN LAW 8<br>DAUGHTER .... 3<br>SON .... 4<br>FRIEND/NEIGHBO .... 5<br>OTHER RELATIVES .... 9<br>OTHERS .... 6<br>NO ONE ELSE .... 7<br>DON'T KNOW / DON'T REMEMBEI 8	HUSBAND .... 1<br>MOTHER .... 2<br>MOTHER IN LAW 8<br>DAUGHTER .... 3<br>SON .... 4<br>FRIEND/NEIGHBOR .... 5<br>OTHER RELATIVES .... 9<br>OTHERS .... 6<br>NO ONE ELSE .... 7<br>DON'T KNOW / DON'T REMEMBE 8	

NO.	QUESTIONS AND FILTERS	CODING CATEGORIES		SKIP
		REFUSED ... 99	REFUSED ... 99	
517	Did she/he ever show you examples of contraceptive methods?	YES ... 1<br>NO ... 2<br>DON'T KNOW ... 88<br>REFUSED ... 99	YES ... 1<br>NO ... 2<br>DON'T KNOW ... 88<br>REFUSED ... 99	
518	Did she/he ever give you brochures or materials about contraceptive methods?	YES ... 1<br>NO ... 2<br>DON'T KNOW ... 88<br>REFUSED ... 99	YES ... 1<br>NO ... 2<br>DON'T KNOW ... 88<br>REFUSED ... 99	
519	Did she/he ever discuss induced abortion with you?	YES ... 1<br>NO ... 2<br>DON'T KNOW ... 88<br>REFUSED ... 99<br>(SKIP TO 521)	YES ... 1<br>NO ... 2<br>DON'T KNOW ... 88<br>REFUSED ... 99<br>(SKIP TO 521)	
520	Did she/he ever provide brochures or materials about induced abortion to you?	YES ... 1<br>NO ... 2<br>DON'T KNOW ... 88<br>REFUSED ... 99	YES ... 1<br>NO ... 2<br>DON'T KNOW ... 88<br>REFUSED ... 99	
521	Did she/he ever refer you to a health facility for family planning or reproductive health issues?	YES ... 1<br>NO ... 2<br>DON'T KNOW ... 88<br>REFUSED ... 99<br>(SKIP TO 523)	YES ... 1<br>NO ... 2<br>DON'T KNOW ... 88<br>REFUSED ... 99<br>(SKIP TO 523)	
522	Did you go there based on this referral?	YES ... 1<br>NO ... 2<br>DON'T KNOW ... 88<br>REFUSED ... 99	YES ... 1<br>NO ... 2<br>DON'T KNOW ... 88<br>REFUSED ... 99	
523	Do you think the information provided by the field worker(s) was reliable?<br><br>(THIS APPLIES TO THE HEALTH WORKER WHO VISITED HER AT HOME)	YES ... 1<br>SOMEWHAT ... 2<br>NO ... 3<br>DON'T KNOW ... 88<br>REFUSED ... 99	YES ... 1<br>SOMEWHAT ... 2<br>NO ... 3<br>DON'T KNOW ... 88<br>REFUSED ... 99	
524	In the past 5 years, have you visited a health facility for care for yourself (or your children)?	YES ... 1<br>NO ... 2<br>REFUSED ... 99		1 → 526
524B	When was the last time you visited a health facility?<br><br>*If less than 12 months ago, answer must be recorded in months, weeks, or days. Enter 0 days for today.*	[ ][ ] Days ago<br>[ ][ ] Weeks ago<br>[ ][ ] Months ago<br>[ ][ ] Years ago<br>DON'T KI ... 88<br>REFUSEI ... 99		#<br>#
525	Why have you not visited a health facility in the past 5 years?<br><br>(Select all that apply)	TOO FAR ... 1<br>TOO EXPENSIVE ... 2<br>NO NEED ... 3<br>OTHER, SPECIFY ... 96<br>REFUSED ... 99		→ 539
526	During any visit in the past 5 years, did any staff member at the health facility or family welfare centre speak to you about family planning methods?	YES ... 1<br>NO ... 2<br>REFUSED ... 99		2, 99 → 539
527	Which health facility did you receive family planning information from the last time?<br><br>PROBE TO IDENTIFY THE TYPE OF FACILITY.	PUBLIC SECTOR<br>GOVT. HOSPITAL/RHSC ... 11<br>RURAL HEALTH CENTER, MCH ... 12<br>MCH CENTER ... 13<br>FAMILY HEALTH/WELFARE CENTER ... 14<br>HEALTH HOUSE (LHV) ... 15		

NO.	QUESTIONS AND FILTERS	CODING CATEGORIES	SKIP
		BASIC HEALTH UNIT (BHU) 16<br>OTHER PUBLIC ______ 17<br>(SPECIFY)<br>PRIVATE/NGO MEDICAL SECTOR<br>PRIVATE/NGO HOSPITAL/CLINIC .... 21<br>PHARMACY .... 23<br>OTHER PRIVATE<br>MEDICAL ______ 27<br>(SPECIFY)<br>OTHER SOURCE<br>NO SERVICES OBTAINED .... 35<br>OTHER ______ 96<br>(SPECIFY)<br>DON'T KNOW 88<br>REFUSED 99	
527B	What was this health facility called? (Name)	______	
528	Who provided you with this information or counseling (during the last facility visit where you received family planning counseling)?<br><br>SELECT ALL THAT APPLY	DOCTOR .... 1<br>NURSE .... 2<br>MIDWIFE .... 3<br>LADY HEALTH WORKER/OUTREACH/<br>PEER EDUCATOR .... 4<br>PHARMACIST .... 5<br>OTHER ______ 96<br>(SPECIFY)<br>DON'T KNOW .... 88<br>REFUSED 99	
529	Which method(s) were you counseled on (during the last facility visit where you received family planning counseling)?<br><br>CIRCLE ALL MENTIONED.	FEMALE STERILIZATION .... 1<br>MALE STERILIZATION .... 2<br>IUD .... 3<br>INJECTABLES .... 4<br>IMPLANTS .... 5<br>PILL .... 6<br>CONDOM .... 7<br>EMERGENCY CONTRACEPTION 11<br>STANDARD DAYS / CALENDAR METHOD .... 12<br>LACTATIONAL AMEN. METHOD .... 13<br>WITHDRAWAL .... 15<br>OTHER MODERN METHOD______ .... 16<br>OTHER TRADITIONAL METHOD______ .... 17<br>NONE .... 18<br>DON'T KNOW .... 88<br>REFUSED 99	
530	Were you married when you had the last counseling/information session?	YES .... 1<br>NO .... 2<br>REFUSED 99	 2 → 532
PK_5_1	In which language(s) were you counseled the last time?<br><br>SELECT ALL THAT APPLY.	URDU .... 1<br>SINDHI .... 2<br>POSHTU .... 3<br>PUNJABI .... 4<br>BALOCHI .... 5<br>ENGLISH .... 6<br>OTHER .... 96<br>DON'T REMEMBER/DON'T KNOW 88<br>REFUSED 99	
531	Did your husband participate in the counseling/information session (during the last facility visit where you received family planning counseling)?	YES .... 1<br>NO .... 2<br>REFUSED 99	
533	Did the person who counseled you ask questions about your health (during the last facility visit where	YES .... 1<br>NO 2	

NO.	QUESTIONS AND FILTERS	CODING CATEGORIES	SKIP
	you received family planning counseling)?	DON'T KNOW .... 88<br>REFUSED 99	
534	Were you counseled on what would happen if you chose NOT to use family planning (during the last facility visit where you received family planning counseling)?	YES .... 1<br>NO .... 2<br>DON'T KNOW .... 88<br>REFUSED 99	
535	Do you feel that you were given enough information about family planning (during the last facility visit where you received family planning counseling)?	YES .... 1<br>NO .... 2<br>DON'T KNOW .... 88<br>REFUSED 99	
536	Are you satisfied with the FAMILY PLANNING COUNSELING services you received (during the last facility visit where you received family planning counseling)?<br>READ OPTIONS ALOUD	FULLY SATISFIED .... 1<br>SOMEWHAT SATISFIED .... 2<br>NOT SATISFIED .... 3<br>DON'T KNOW .... 88<br>REFUSED 99	
537	Do you think the family planning information provided by this service provider is reliable? (during the last facility visit where you received family planning counseling)?<br>READ OPTIONS ALOUD	YES .... 1<br>SOMEWHAT .... 2<br>NO .... 3<br>DON'T KNOW .... 88<br>REFUSED 99	
538	Would you recommend services from this provider to a friend or a relative in a similar situation as you?	YES .... 1<br>NO .... 2<br>DON'T KNOW .... 88<br>REFUSED 99	
539	Have you ever used anything or tried in any way to delay or avoid getting pregnant?<br>PROMPT for sterilization and traditional methods	YES .... 1<br>NO .... 2<br>REFUSED 99	
539B	Just to confirm, have you been sterilized?	YES .... 1<br>NO .... 2<br>REFUSED 99	2, 99 → 541
540	Which method(s) have you ever used?<br>CIRCLE ALL MENTIONED.<br>PROMPT for sterilization and traditional methods	FEMALE STERILIZATION .... 1<br>MALE STERILIZATION .... 2<br>IUD .... 3<br>INJECTABLES .... 4<br>IMPLANTS .... 5<br>PILL .... 6<br>CONDOM .... 7<br>EMERGENCY CONTRACEPTION 11<br>STANDARD DAYS / CALENDAR / RHYTHM M .... 12<br>LACTATIONAL AMEN. METHOD .... 13<br>WITHDRAWAL .... 15<br>OTHER MODERN METHOD_____ .... 16<br>OTHER TRADITIONAL METHOD_____ .... 17<br>NONE .... 18<br>DON'T KNOW .... 88<br>REFUSED 99	
541	In choosing a contraceptive method, what feature would be most important to you?<br>(SELECT ONE: PROBE FOR MOST IMPORTANT REASON TO THE RESPONDENT)	HOW EFFECTIVE IT IS AT PREVENTING PREGNANCY .... 1<br>CAN BE USED WITHOUT ANYONE ELSE KNOWING .... 2<br>THAT IT PROTECTS AGAINST STIs/HIV .... 3<br>NO RISK OF HARMING HEALTH 4<br>NO EFFECT ON REGULAR MONTHLY BLEEDING 5<br>NO UNPLEASANT SIDE EFFECTS 6<br>EASY TO USE 7<br>EASY TO OBTAIN 8<br>AFFORDABLE 9<br>CAN BE USED FOR A LONG TIME WITHOUT NEED TO VISIT CLINIC OR RE-SUPPLY 10<br>WILL BE ABLE TO GET PREGNANT WHEN I WANT 11<br>OTHER _____ (SPECIFY) 96<br>DON'T KNOW .... 88<br>REFUSED 99	
542	CHECK Q. 539:		

NO.	QUESTIONS AND FILTERS	CODING CATEGORIES	SKIP
	EVER USED FAMILY PLANNING ↓	NEVER USED FAMILY PLANNING □ →	548
543	CHECK Q. 420:<br>NOT PREGNANT OR UNSURE □ ↓	PREGNANT □ →	555
544	Are you or your husband currently doing something or using any method to delay or avoid getting pregnant?<br>PROMPT for sterilization and traditional methods	YES .... 1<br>NO .... 2<br>DON'T KNOW .... 88<br>REFUSEI 99	 547
545	Which method(s) are you or your husband currently using?<br><br>RECORD ALL MENTIONED.	FEMALE STERILIZATION .... 1<br>MALE STERILIZATION .... 2<br>IUD .... 3<br>INJECTABLES .... 4<br>IMPLANTS .... 5<br>PILL .... 6<br>CONDOM .... 7<br><br>EMERGENCY CONTRACEPTION 11<br>STANDARD DAYS / CALENDAR / RHYTHM M .... 12<br>LACTATIONAL AMEN. METHOD .... 13<br>WITHDRAWAL .... 15<br>OTHER MODERN METHOD______ .... 16<br>OTHER TRADITIONAL METHOD______ .... 17<br><br>DON'T KNOW .... 88<br>REFUSED 99	 547
546	Since what month and year have you been using (CURRENT METHOD) without stopping?<br><br>PROBE: For how long have you been using (CURRENT METHOD) now without stopping?	MONTH .... [ ][ ]<br>YEAR .... [ ][ ][ ][ ]<br>DON'T KNOW .... 88/8888<br>REFUSED 99/9999	
547	Are you currently breastfeeding to delay or avoid getting pregnant?	YES .... 1<br>NO .... 2<br>DON'T KNOW .... 88<br>REFUSEI 99	
548	CHECK Q. 424:<br>GAVE BIRTH IN PAST 6 MONTHS □ ↓	DID NOT GIVE GIVE BIRTH IN PAST 6 MONTHS □ →	553
549	Did you breastfeed your most recent baby at any time after giving birth?	YES .... 1<br>NO .... 2<br>REFUSED 99	552
550	Are you still breastfeeding this baby?	YES .... 1<br>NO .... 2<br>REFUSED 99	552
551	Have you given the baby anything but breastmilk since he/she was born?	YES .... 1<br>NO .... 2<br>DON'T KNOW 88<br>REFUSED 99	
552	Has your period returned?	YES .... 1<br>NO .... 2<br>DON'T KNOW 88<br>REFUSED 99	
553	CHECK Q. 539:<br>EVER USED □ ↓	NEVER USED FAMILY PLANNING □ →	579

NO.	QUESTIONS AND FILTERS	CODING CATEGORIES	SKIP
	FAMILY PLANNING		
554	CHECK Q. 544:<br>NOT CURRENTLY USING FAMILY PLANNING ☐ ↓	CURRENTLY USING FAMILY PLANNING ☐ →	556
555	You mentioned that you are not currently using a family planning method. Which method(s) did you use last?<br><br>RECORD ALL MENTIONED.<br><br>IF MORE THAN ONE METHOD MENTIONED, FOLLOW SKIP INSTRUCTION FOR HIGHEST METHOD IN LIST.	FEMALE STERILIZATION ..... 1<br>MALE STERILIZATION ..... 2<br>IUD ..... 3<br>INJECTABLES ..... 4<br>IMPLANTS ..... 5<br>PILL ..... 6<br>CONDOM ..... 7<br><br>EMERGENCY CONTRACEPTION 11<br>STANDARD DAYS / CALENDAR / RHYTHM M ..... 12<br>LACTATIONAL AMEN. METHOD ..... 13<br>WITHDRAWAL ..... 15<br>OTHER MODERN METHOD______ ..... 16<br>OTHER TRADITIONAL METHOD______ ..... 17<br>NONE ..... 18<br>DON'T KNOW ..... 88<br>REFUSED 99	→ 575 (NONE, DON'T KNOW, REFUSED)
556	Where did you obtain [THE LAST/CURRENT METHOD] the last time?<br><br>PROBE TO IDENTIFY THE TYPE OF SOURCE.<br><br>[Only for methods 1-11 in Question 545/555]	PUBLIC SECTOR<br>GOVT. HOSPITAL/RHSC ..... 11<br>RURAL HEALTH CENTRE, MCH ..... 12<br>MCH ..... 13<br>FAMILY HEALTH/WELFARE CENTRE ..... 14<br>MOBILE SERVICE CAMP ..... 15<br>LADY HEALTH WORKER 16<br>LADY HEALTH VISITOR 17<br>BASIC HEALTH UNIT 18<br>MALE MOBILIZER 19<br>FWA 20<br>OTHER PUBLIC ______ 21<br>(SPECIFY)<br><br>PRIVATE/NGO MEDICAL SECTOR<br>PRIVATE/NGO HOSPITAL/CLINIC ..... 22<br>PHARMACY, CHEMISTS ..... 23<br>PRIVATE DOCT ..... 24<br>HOMEOPATH ..... 25<br>DISPENSOR/ COMPUNCER 26<br>MOBILE SERVICE CAMP 27<br>OTHER PRIVATE MEDICAL ______ 28<br>(SPECIFY)<br><br>OTHER SOURCE<br>SHOP (NOT PHARMACY/CHEMIST) 31<br>FRIEND/RELATIVE 32<br>HAKIM ..... 33<br>DAI, TRAD. BIRTH ATTENDANT ..... 34<br><br>OTHER ______ 96<br>(SPECIFY)<br>DON'T KNOW 88<br>REFUSED 99	16–17 → 558<br>19–20 → 558<br>23–27 → 558<br>31–99 → 558
556B	What was the name of the facility<br>[Only for methods 1-11 in Question 545/555]	NAME OF FACILITY ______	
558	How many minutes did it take for you to travel to this (SERVICE PROVIDER) to receive [THE LAST/CURRENT METHOD]?<br><br>[Only for methods 1-11 in Question 545/555]	MINUTES ..... ☐☐☐☐<br><br>REFUSED 99	
559	What mode(s) of transportation did you use to travel to this (SERVICE PROVIDER) to receive [THE LAST/CURRENT METHOD]?<br><br>[Only for methods 1-11 in Question 545/555]	NONE (RECEIVED AT HOME) 1<br>WALK 2<br>BICYCLE 3<br>MOTORCYCLE ..... 4<br>BUS ..... 5<br>CAR / TAXI 6<br>OTHER ..... 96<br>DON'T KNOW 88	

NO.	QUESTIONS AND FILTERS	CODING CATEGORIES	SKIP
		REFUSED 99	
560	How much did you have to pay in total to obtain [THE LAST/CURRENT METHOD]?<br><br>IF NONE ENTER '0000'<br>IF DON'T KNOW ENTER '8888'<br>IF REFUSED ENTER '9999'<br><br>[Only for methods 1-11 in Question 545/555]	COST OF METHOD [ ][ ][ ][ ]<br>COST OF SERVICE [ ][ ][ ][ ]<br>TRANSPORTATION [ ][ ][ ][ ]<br>LOST WAGES [ ][ ][ ][ ]<br>CHILDCARE [ ][ ][ ][ ]	
561	How many minutes did you have to wait at the (SERVICE PROVIDER) before you received [THE LAST/CURRENT METHOD]?<br><br>[Only for methods 1-11 in Question 545/555]<br>IF DON'T KNOW ENTER '888'<br>IF REFUSED ENTER '999'	MINUTES .... [ ][ ][ ]<br>DON'T KNOW .... 8888<br>REFUSED 9999	
562	Did you feel that you got enough information about [THE LAST/CURRENT METHOD]?<br><br>[Only for methods 1-11 in Question 545/555]	YES .... 1<br>NO .... 2<br>DON'T KNOW .... 88<br>REFUSED 99	
563	Did you receive any counseling or information from a health or family planning worker about [THE LAST/CURRENT METHOD]?<br>[Only for methods 1-11 in Question 545/555]	YES .... 1<br>NO .... 2<br>DON'T KNOW .... 88<br>REFUSED 99	 → 576
564	Did the person who counseled you explain how [THE LAST/CURRENT METHOD] works to prevent pregnancy?<br>[Only for methods 1-11 in Question 545/555]	YES .... 1<br>NO .... 2<br>DON'T KNOW .... 88<br>REFUSED 99	
565	Did the person who counseled you on [THE LAST/CURRENT METHOD] explain how to use it?<br><br>[Only for methods 1-11 in Question 545/555]	YES .... 1<br>NO .... 2<br>DON'T KNOW .... 88<br>REFUSED 99	
566	Did the person who counseled you allow you to ask questions?<br>[Only for methods 1-11 in Question 545/555]	YES .... 1<br>NO .... 2<br>DON'T KNOW .... 88<br>REFUSED 99	
567	When you got [THE LAST/CURRENT METHOD], were you told by a health or family planning worker about other methods of family planning that you could use?<br>[Only for methods 1-11 in Question 545/555]	YES .... 1<br>NO .... 2<br>DON'T KNOW .... 88<br>REFUSED 99	
568	When you got [THE LAST/CURRENT METHOD], were you told by a health or family planning worker about potential side effects or problems you might have with the method(s)?<br>[Only for methods 1-11 in Question 545/555]	YES .... 1<br>NO .... 2<br>DON'T KNOW .... 88<br>REFUSED 99	
569	When you got [THE LAST/CURRENT METHOD], were you told what to do if you experienced side effects or problems?<br><br>[Only for methods 1-11 in Question 545/555]	YES .... 1<br>NO .... 2<br>DON'T KNOW .... 88<br>REFUSED 99	
570	When you got [THE LAST/CURRENT METHOD], were you told about any benefits?<br><br>[Only for methods 1-11 in Question 545/555]	YES .... 1<br>NO .... 2<br>DON'T KNOW .... 88<br>REFUSED 99	
571	When you got [THE LAST/CURRENT METHOD], were you told of any disadvantages?<br>[Only for methods 1-11 in Question 545/555]	YES .... 1<br>NO .... 2<br>DON'T KNOW .... 88<br>REFUSED 99	
576	Was the method you wanted available to you?	YES .... 1<br>NO 2	

NO.	QUESTIONS AND FILTERS	CODING CATEGORIES		SKIP
		DON'T KNOW	88	
		REFUSED	99	
577	What method had you wanted at that time?<br><br>RECORD ALL MENTIONED.	FEMALE STERILIZATION<br>MALE STERILIZATION<br>IUD<br>INJECTABLES<br>IMPLANTS<br>PILL<br>CONDOM<br><br>EMERGENCY CONTRACEPTION<br>STANDARD DAYS / CALENDAR /RHYTHM MI<br>LACTATIONAL AMEN. METHOD<br>WITHDRAWAL<br>OTHER MODERN METHOD_____________<br>OTHER TRADITIONAL METHOD_______________<br>NONE<br>DON'T KNOW<br>REFUSED	1<br>2<br>3<br>4<br>5<br>6<br>7<br><br>11<br>12<br>13<br>15<br>16<br>17<br>18<br>88<br>99	
578	What was the MOST important reason you didn't obtain the method you wanted?	METHOD OUT OF STOCK THAT DAY<br>METHOD NOT AVAILABLE AT ALL<br>PROVIDER NOT TRAINED TO PROVIDE METHOD<br>PROVIDER RECOMMENDED ANOTHER METHOD<br>NOT ELIGIBLE FOR METHOD<br>DECIDED NOT TO ADOPT A METHOD<br>DECIDED TO ADOPT ANOTHER METHOD<br>TOO COSTLY<br>HUSBAND / PARTNER REFUSED<br>DIFFICULT TO ACCESS HEALTH CENTER<br>OTHER, SPECIFY _________________________<br>DON'T KNOW<br>REFUSED	1<br>2<br>3<br>4<br>5<br>6<br>7<br>8<br>9<br>10<br>11<br>88<br>99	
572	Did you feel pressured into using [THE LAST/CURRENT METHOD]?	YES<br>NO<br>DON'T KNOW<br>REFUSED	1<br>2<br>88<br>99	
573	Did you feel you could say no to [THE LAST/CURRENT METHOD]?	YES<br>NO<br>DON'T KNOW<br>REFUSED	1<br>2<br>88<br>99	
574	Has the [THE LAST/CURRENT METHOD] caused any problems?	YES<br>NO<br>DON'T KNOW<br>REFUSED	1<br>2<br>88<br>99	
575	Do you feel that you had access to a wide range of family planning methods, or only to a select few?	A WIDE RANG<br>A SELECT FEW<br>DON'T KNOW<br>REFUSED	1<br>2<br>88<br>99	
579	CHECK Q. 539:<br><br>NEVER USED FAMILY PLANNING ☐ ↓	EVER USED FAMILY PLANNING ☐ →		585
580	Do you feel that you have access to a wide range of family planning methods?<br><br>READ OPTIONS OUT LOUD	A WIDE RANG<br>A SELECT FEW<br>NO ACCESS<br>DON'T KNOW<br>REFUSED	1<br>2<br>3<br>88<br>99	
581	Have you ever wanted to use a method of family planning?	YES<br>NO<br>DON'T KNOW<br>REFUSED	1<br>2<br>88<br>99	]<br>→585<br>]
582	Is the method of family planning you would want to use available to you?	YES<br>NO<br>DON'T KNOW<br>REFUSED	1<br>2<br>88<br>99	

NO.	QUESTIONS AND FILTERS	CODING CATEGORIES	SKIP
583	What method do you want or have you wanted most recently?<br><br>RECORD ALL MENTIONED.	FEMALE STERILIZATION .... 1<br>MALE STERILIZATION .... 2<br>IUD .... 3<br>INJECTABLES .... 4<br>IMPLANTS .... 5<br>PILL .... 6<br>CONDOM .... 7<br><br>EMERGENCY CONTRACEPTION 11<br>STANDARD DAYS / CALENDAR / RHYTHM M .... 12<br>LACTATIONAL AMEN. METHOD .... 13<br>WITHDRAWAL .... 15<br>OTHER MODERN METHOD_____________ .... 16<br>OTHER TRADITIONAL METHOD_____________ .... 17<br>NONE .... 18<br>DON'T KNOW .... 88<br>REFUSED 99	
584	What is the MOST important reason you have not obtained the method you wanted?	METHOD OUT OF STOCK 1<br>METHOD NOT AVAILABLE AT ALL 2<br>PROVIDER NOT TRAINED TO PROVIDE METHOD 3<br>PROVIDER RECOMMENDED ANOTHER METHOD 4<br>NOT ELIGIBLE FOR METHOD 5<br>DECIDED NOT TO ADOPT A METHOD 6<br>DECIDED TO ADOPT ANOTHER METHOD 7<br>TOO COSTLY 8<br>HUSBAND/PARTNER REFUSED 9<br>DIFFICULT TO ACCESS HEALTH CENTER 10<br>OTHER, SPECIFY _______________________ 11<br>DON'T KNOW 88<br>REFUSED 99	
585	Does your husband support you in your choices related to contraceptive use?	YES .... 1<br>SOMETIMES .... 3<br>NO .... 2<br>DON'T KNOW .... 88<br>REFUSED 99	
586	CHECK:<br>ANY BIRTH OR PREGNANCY TERMINATION AFTER MONTH AND YEAR OF START OF USE OF CONTRACEPTION IN Q. 546 — YES ☐ NO ☐<br><br>GO BACK TO **546** PROBE AND RECORD MONTH AND YEAR AT START OF CONTINUOUS USE OF CURRENT METHOD (MUST BE AFTER LAST BIRTH OR PREGNANCY TERMINATION).		

6. PREGNANCY AND CHILD HEALTH

601	REVIEW Q417<br>IF THE WOMAN HAS GIVEN BIRTH IN THE LAST 80 MONTHS, ASK HER THE QUESTIONS ABOUT HER LAST BIRTH. IF NOT SKIP THIS SECTION.<br><br>ONE OR MORE BIRTHS IN LAST 80 MO./years ☐ NO BIRTHS 80 MO./5 years ☐ → 701<br><br>Now I would like to ask some questions about your most recent birth(s).	

		LAST BIRTH	NEXT TO LAST BIRTH
602	When you got pregnant with this last/next to last] baby, did you want to get pregnant	YES .... 1 (SKIP TO 605)<br>NO 2	YES .... 1 (SKIP TO 605)<br>NO 2
603	Did you want to have a baby later on, or did you not want any children?	LATER .... 1<br>NO MORE/NONE . 2<br>(SKIP TO 605)	LATER 1<br>NO MORE/NC.... 2<br>(SKIP TO 605)
604	How much longer did you want to wait?<br><br>RECORD IN UNIT RESPONDENT USES	MONTHS 1 ☐☐<br>YEARS 2 ☐☐<br>DON'T KNOW 88	MONTHS ☐☐<br>YEARS ☐☐<br>DON'T KNOW 88
605	Did you see anyone for antenatal care for this pregnancy?	YES .... 1<br>NO .... 2<br>(SKIP TO 611)	YES .... 1<br>NO .... 2<br>(SKIP TO 611)
606	Whom did you see?<br><br>Anyone else?<br><br>PROBE TO IDENTIFY EACH TYPE OF PERSON AND RECORD ALL	**HEALTH PERSONNEL**<br>DOCT........... 1<br>NURSE/MIDWI... 2<br>COM. HEALTH ... 3<br>**OTHER PERSON**<br>TRADITIONAL BIRTH ATTENDA..... 4<br>LHW/LHV 5<br>TRAD. HEALTH PRACTITIONER 6<br>OTHER 7<br>(SPECIFY)<br>NO ONE ASSISTED 10<br>DON'T KNOW 88<br>REFUSED 99	**HEALTH PERSONNEL**<br>DOCTOR 1<br>NURSE....... 2<br>COM. HE..... 3<br>**OTHER PERSON**<br>TRADITIONAL BIRTH .... 4<br>LHW/LHV 5<br>TRAD. HEALTH 6<br>OTHER 7<br>(SPECIFY<br>NO ONE ASSISTED 10<br>DON'T KNOW 88<br>REFUSED 99
607	Where did you receive antenatal care for this pregnancy?<br>Anywhere else?	**HOME**<br>RESPOI......... 1<br>OTHER HOM..... 2<br>**PUBLIC SECTOR**<br>GOVERNMENT .. 11	**HOME**<br>RES......... 1<br>OTH........ 2<br>**PUBLIC SECTOR**<br>GOVERNMI... 11

	PROBE TO IDENTIFY THE TYPE OF SOURCE.<br><br>IF UNABLE TO DETERMINE IF PUBLIC OR PRIVATE SECTOR, WRITE THE NAME OF THE PLACE<br><br>_______________<br>(NAME OF PLACE)	RCH/MCH 18<br>BASIC H......... 12<br><br>OTHER PUBLIC SECTOR<br>_________ 17<br>(SPECIFY)<br><br>**PRIVATE MEDICAL SECTOR**<br>PRIVATE/NGO HOSPITAL/ CLINI......... 21<br>PVT. DOCTOR 22<br>HOMEOPATH 23<br>OTHER PRIVATE:<br>_________ 27<br>(SPECIFY)<br><br>OTHER _________ 96<br>(SPECIFY)<br>REFUSED 99	RCH/MCH 18<br>BAS......... 12<br><br>OTHER PUBLIC SECTOR<br>_________ 17<br>(SPECIFY)<br><br>**PRIVATE MEDICAL SECTOR**<br>PRIVATE/NGO HOSPITAL/ .... 21<br>PVT. DOCTOR 22<br>HOMEOPATH 23<br>OTHER PRIVATE:<br>_________ 27<br>(SPECIFY)<br><br>OTHER _________ 96<br>(SPECIFY)<br>REFUSED 99
608	How many months pregnant were you when you first received antenatal care for this pregnancy?	MONTHS [ ][ ]<br><br>DON'T KNOW 88<br>REFUSED 99	MONTHS [ ][ ]<br><br>DON'T KNOW 88<br>REFUSED 99
609	How many times did you receive antenatal care during this pregnancy (at facility or visit at home by provider)?	NUMBER OF TIMES [ ][ ]<br><br>DON'T KNOW 88<br>REFUSED 99	NUMBER OF TIMES [ ][ ]<br><br>DON'T KNOW 88<br>REFUSED 99
610	Did you ever receive counseling on family planning during your antenatal care?	YES .... 1<br>NO .... 2<br>REFUSED 99	YES 1<br>NO 2<br>REFUSED 99
612	Where did you give birth?	**HOME**<br>RESPOI......... 1<br>OTHER HON..... 2<br><br>**PUBLIC SECTOR**	**HOME**<br>RES......... 1<br>OTH........ 2<br><br>**PUBLIC SECTOR**

	PROBE FOR THE TYPE OF SOURCE.<br><br>IF UNABLE TO DETERMINE IF PUBLIC OR PRIVATE SECTOR, WRITE THE NAME OF THE PLACE.<br><br>______________<br>(NAME OF PLACE)	GOVERNMENT . . 11<br>RCH/MCH 18<br>BASIC I . . . . 12<br><br>OTHER PUBLIC SECTOR<br>______________ 17<br>(SPECIFY)<br><br>**PRIVATE MEDICAL SECTOR**<br>PRIVATE/NGO HOSPITAL/<br>CLINI . . . . 21<br>PVT. DOCTOR 22<br>HOMEOPATH 23<br>OTHER PRIVATE:<br>______________ 27<br>(SPECIFY)<br><br>OTHER ______________ 96<br>(SPECIFY)<br>REFUSED 99	GOVERNMI . . . 11<br>RCH/MCH 18<br>BAS . . . . 12<br><br>OTHER PUBLIC SECTOR<br>______________ 17<br>(SPECIFY)<br><br>**PRIVATE MEDICAL SECTOR**<br>PRIVATE/NGO HOSPITAL/<br>. . . . . 21<br>PVT. DOCTOR 22<br>HOMEOPATH 23<br>OTHER PRIVATE:<br>______________ 27<br>(SPECIFY)<br><br>OTHER ______________ 96<br>(SPECIFY)<br>REFUSED 99
611	Did anyone assist with the delivery of this baby?<br>Who assisted?<br>Anyone else?<br><br>PROBE FOR THE TYPE(S) OF PERSONS(S) AND RECORD ALL MENTIONED.<br><br>IF RESPONDENT SAYS NO ONE ASSISTED, PROBE TO DETERMINE WHETHER ANY ADULTS WERE PRESENT AT THE DELIVERY.	**HEALTH PERSONNEL**<br>DOCTOR . 1<br>NURSE/MIDWIFE 2<br>COM. HEALTH OFFICER/ NURSE . . 3<br><br>**OTHER PERSON**<br>TRAD. BIRTH ATTENDANT/ TBA 4<br>LHW/LHV 5<br><br>TRAD. HEALTH PRACTITIONER 6<br>RELATIVE / NEIGHE 7<br>OTHER<br>______________ 8<br>(SPECIFY)<br><br>NO ONE ASSISTED 10<br>DON'T KNOW 88<br>REFUSED 99	**HEALTH PERSONNEL**<br>DOCT(. . . 1<br>NURSE/MIDWIF 2<br>COM. HEALTH<br>. . . . . 3<br><br>**OTHER PERSON**<br>TRAD. BIRTH<br>4<br>LHW/LHV 5<br><br>TRAD. HEALTH PRACTITIO 6<br>RELATIVE / NEI 7<br>OTHER<br>______________ 8<br>(SPECIFY)<br><br>NO ONE ASSISTEI 10<br>DON'T KNOW 88<br>REFUSED 99
613	Did you have a post-natal care visit for this child at any time in the six weeks after the birth?	YES . . . . 1<br>NO . . . . 2<br>NO, BABY DIED...... 3<br>DON'T KNOW . . 88<br>REFUSE 99	YES 1<br>NO 2<br>NO, BABY DIE 3<br>DON'T . . . . . 88<br>REFUSE 99
614	Did you receive family planning counselling at any post-natal visit?	YES . . . . 1<br>NO . . . . 2<br>DON'T KNOW . . 88<br>REFUSE 99	YES 1<br>NO 2<br>DON'T . . . . . 88<br>REFUSE 99

7. MARRIAGE

	Now I would like to ask you some questions about your partners and unions or marriages. Let me assure you again that your answers are completely confidential and will not be told to anyone. If we should come to any question that you don't want to answer, just let me know and we will go to the next question.<br><br>CHECK FOR THE PRESENCE OF OTHERS. BEFORE CONTINUING, MAKE EVERY EFFORT TO ENSURE PRIVACY.

NO.	QUESTIONS AND FILTERS	CODING CATEGORIES	SKIP
706	Does your husband currently live with you?	YES . . . . 1<br>NO . . . . 2<br>REFUSED 99	1 → 703<br><br>99 → 703
707	How long has your husband been away?<br>RECORD IN UNIT RESPONDENT USES	MONTHS . . . . [ ][ ]<br>WEEKS . . . . [ ][ ]<br>YEARS [ ][ ]<br>DON'T KNOW 88<br>REFUSED 99	
PK_7_1	Does your (husband) have other wives or does he live with other women as if married?	YES . . . . 1<br>NO . . . . 2<br>DON'T KNOW . . . . 8<br>REFUSED 99	2, 8, 99 → PK_7_4
PK_7_2	Including yourself, in total, how many wives does he have?	TOTAL NUMBER OF WIVES . . . [ ][ ]<br>DON'T KNOW . . . . 98<br>REFUSED 99	
PK_7_3	Are you the first, second, ... wife?<br><br>IF REFUSED, ENTER '99'	RANK . . . . [ ][ ]	
PK_7_4	In 2013 did your (husband) have other wives or does he lived with other women as if married?	YES . . . . 1<br>NO . . . . 2<br>DON'T KNOW . . . . 8<br>REFUSED 99	2, 8, 99 → 703
PK_7_5	Including yourself, in total, how many wives did he have in 2013?	TOTAL NUMBER OF WIVES . . . [ ][ ]<br>DON'T KNOW . . . . 98<br>REFUSED 99	
PK_7_6	At that time, were you the first, second, ... wife?<br><br>IF REFUSED, ENTER '99'	RANK . . . . [ ][ ]	
703	Have you been married only once or more than once?	ONLY ONCE . . . . 1<br>MORE THAN ONCE . . . . 2<br>REFUSED 99	

SECTION 8. FERTILITY PREFERENCES

NO.	QUESTIONS AND FILTERS	CODING CATEGORIES	SKIP
802	CHECK 420:<br>PREGNANT ☐ ↓	NOT PREGNANT OR UNSURE ☐	→ 804
803	SKIP if Q539B = 1<br>Now I have some questions about the future. After the child you are expecting now, would you like to have another child, or would you prefer not have any more children?	HAVE ANOTHER CHILD . . . . 1<br>NO MORE . . . . 2<br>UNDECIDED/DON'T KNOW . . . . 88<br>REFUSED 99	→ 805<br>→ 811 (2, 88, 99)
804	SKIP if Q539B = 1<br>Now I have some questions about the future. Would you like to have (a/another) child, or would you prefer not to have any (more) children?	HAVE (A/ANOTHER) CHILD . . . . 1<br>NO MORE/NONE . . . . 2<br>SAYS SHE CAN'T GET PREGNANT . . . . 3<br>UNDECIDED/DON'T KNOW . . . . '88<br>REFUSED 99	 → 807<br>→ 812<br>→ 810
805	SKIP if Q539B = 1<br>CHECK 420:<br>NOT PREGNANT OR UNSURE ☐ ↓ | PREGNANT ☐ ↓<br>a) How long would you like to wait from now before the birth of (a/another) child?<br>b) After the birth of the child you are expecting now, how long would you like to wait before the birth of another child?	MONTHS . . . . 1 [ ][ ]<br>YEARS . . . . 2 [ ][ ]<br>SOON/NOW . . . . 93<br>SAYS SHE CAN'T GET PREGNANT . . . . 94<br>AFTER MARRIAGE . . . . 95<br>OTHER ________________ (SPECIFY) 96<br>DON'T KNOW . . . . 98<br>REFUSED 99	 <br>→ 810<br>→ 812<br>→ 810 (95, 96, 98)
806	CHECK 420:<br>NOT PREGNANT OR UNSURE ☐ ↓	PREGNANT ☐	→ 811
807	CHECK 544: USING A CONTRACEPTIVE<br>NOT CURRENTLY USING ☐ ↓	CURRENTLY USING ☐	→ 812
808	CHECK 805:<br>'24' OR MORE MONTHS INTEND TO SPACE OR '02' OR MORE YEARS ☐ ↓<br>NOT ASKED ☐ ↓	'00-23' MONTHS INTEND TO LIMIT OR '00-01' YEAR ☐	→ 811

SECTION 8. FERTILITY PREFERENCES

NO.	QUESTIONS AND FILTERS	CODING CATEGORIES	SKIP
809	SKIP if Q539B = 1<br>CHECK 804:<br>WANTS TO HAVE A/ANOTHER CHILD ☐ ↓ | WANTS NO MORE/ NONE ☐ ↓<br>a) You have said that you want (a/another) child, but not until later. Can you tell me why you are not using a method to prevent pregnancy?<br>Any other reason?<br>b) You have said that you do not want any (more) children. Can you tell me why you are not using a method to prevent pregnancy?<br>Any other reason?<br>RECORD ALL REASONS MENTIONED.	**FERTILITY-RELATED REASONS**<br>NOT HAVING SEX . . . 2<br>INFREQUENT SEX . . . 3<br>MENOPAUSAL/HYSTERECTOMY . . . 4<br>CAN'T GET PREGNANT . . . 5<br>NOT MENSTRUATED SINCE LAST BIRTH . . . 6<br>BREASTFEEDING . . . 7<br>UP TO GOD/FATALISTIC . . . 8<br>**OPPOSITION TO USE**<br>RESPONDENT OPPOSED . . . 9<br>HUSBAND/PARTNER OPPOSED . . . 10<br>OTHERS OPPOSED . . . 11<br>RELIGIOUS PROHIBITION . . . 12<br>STIGMATIZED TO USE IN COMMUNITY . . . 13<br>**LACK OF KNOWLEDGE**<br>KNOWS NO METHOD . . . 14<br>KNOWS NO SOURCE . . . 15<br>**METHOD-RELATED REASONS**<br>SIDE EFFECTS/HEALTH CONCERNS . . . 16<br>LACK OF ACCESS/TOO FAR . . . 17<br>METHOD COSTS TOO MUCH . . . 18<br>VISIT TO CLINIC SCOSTS TOO MUCH. . . 19<br>CLINIC OPERATING HOURS LIMITED 20<br>NO DOCTOR OR PROVIDER AVAILABLE 21<br>PREFERRED METHOD NOT AVAILABLE . . . 22<br>NO METHOD AVAILABLE . . . 23<br>INCONVENIENT TO USE . . . 24<br>INTERFERES WITH BODY'S NORMAL PROCESSES . . . 25<br>OTHER ________ 96<br>(SPECIFY)<br>DON'T KNOW . . . 88<br>REFUSED . . . 99	
810	CHECK 544: USING A CONTRACEPTIVE<br>NOT ASKED ☐ ↓ NO, NOT CURRENTLY USING ☐ ↓ YES, CURRENTLY USING ☐ → 812		812
811	SKIP if Q539B = 1<br>Do you think you will use a contraceptive method to delay or avoid pregnancy at any time in the future?	YES . . . 1<br>NO . . . 2<br>DON'T KNOW . . . 88<br>REFUSED . . . 99	
812	CHECK 410 & 412:<br>HAS LIVING CHILDREN ☐ ↓ | NO LIVING CHILDREN ☐ ↓<br>a) If you could go back to the time you did not have any children and could choose exactly the number of children to have in your whole life, how many would that be?<br>b) If you could choose exactly the number of children to have in your whole life, how many would that be?<br>PROBE FOR A NUMERIC RESPONSE.	NONE . . . 0<br>NUMBER . . . [ ][ ]<br>________<br>DON'T KNOW . . . 88<br>REFUSED . . . 99	NONE → 814<br><br>DON'T KNOW → 814<br>REFUSED → 814
813	How many of these children would you like to be boys, how many would you like to be girls and for how many would it not matter if it's a boy or a girl?	BOYS / GIRLS / EITHER<br>NUMBER . . [ ][ ] [ ][ ] [ ][ ]<br>OTHER ________ 96<br>(SPECIFY)	

NO.	QUESTIONS AND FILTERS	CODING CATEGORIES	SKIP
		DON'T KNOW .... 88<br>REFUSED 99	
814	CHECK 544: USING A CONTRACEPTIVE<br>CURRENTLY USING ☐ ↓ NOT CURRENTLY USING ☐		→ 817
815	SKIP if Q545 = 2 or if Q539B = 1<br>Does your husband know that you are using a method of family planning?	YES .... 1<br>NO .... 2<br>DON'T KNOW .... 88<br>REFUSED 99	(2, 88, 99) → 818
816	SKIP if Q539B = 1<br>Now I have some questions about your current situation. Would you say that using contraception is mainly your decision, mainly your husband's decision, or did you both decide together?	MAINLY RESPONDENT .... 1<br>MAINLY HUSBAND .... 2<br>JOINT DECISION .... 3<br>OTHER ______ (SPECIFY) 96<br>DON'T KNOW .... 88<br>REFUSED 99	→ 818
817	SKIP if Q539B = 1<br>Now I have some questions about your current situation. Would you say that **NOT** using contraception is mainly your decision, mainly your husband's decision, or did you both decide together?	MAINLY RESPONDENT .... 1<br>MAINLY HUSBAND .... 2<br>JOINT DECISION .... 3<br>OTHER ______ (SPECIFY) 96<br>DON'T KNOW .... 88<br>REFUSED 99	
818	Does your husband want the same number of children that you want, or does he want more or fewer than you want?	SAME NUMBER .... 1<br>MORE CHILDREN .... 2<br>FEWER CHILDREN .... 3<br>DON'T KNOW .... 88<br>REFUSED 99	
819	SKIP if Q539B = 1<br>If you were to not use/since you are not using any family planning method, how likely do you think it is that you will become pregnant during the next year?	LIKELY .... 1<br>UNSURE/NEUTRAL .... 2<br>UNLIKELY .... 3<br>DON'T KNOW .... 88<br>REFUSED 99	
820	CHECK 544: USING A CONTRACEPTIVE<br>CURRENTLY USING ☐ ↓ NOT ASKED OR NOT CURRENTLY USING ☐		→ 825
821	SKIP if Q539B = 1<br>If you were to continue to use your family planning method, how likely do you think it is that you would become pregnant during the next year?	LIKELY .... 1<br>UNSURE/NEUTRAL .... 2<br>UNLIKELY .... 3<br>DON'T KNOW .... 88<br>REFUSED 99	
822	SKIP if Q539B = 1<br>Different methods of family planning vary in how effective or ineffective they are in preventing pregnancy. How effective do you think that your family planning method is in preventing pregnancy?	EFFECTIVE .... 1<br>SOMEWHAT EFFECTIVE .... 2<br>UNSURE/NEUTRAL .... 3<br>SOMEWHAT INEFFECTIV 4	

9. HUSBAND'S BACKGROUND AND WOMEN'S STATUS

NO.	QUESTIONS AND FILTERS	CODING CATEGORIES	SKIP
902	How old was your husband on his last birthday?	AGE IN COMPLETED YEARS [ ][ ]<br>DON'T KNOW 888<br>REFUSED 999	
902B	Can your husband read a phrase/sentence in English?	YES .... 1<br>NO .... 2<br>DON'T KNOW .... 88<br>REFUSED 99	
903	What is your husband's/partner's occupation?<br>That is, what kind of work does he mainly do?	PROFESSIONAL/TECHNICAL 1<br>DAILY WAGE LABORER .... 2<br>CIVIL SERVANT .... 3<br>PRODUCTION/TRANSPOI .... 4<br>DAILY WAGE LABOURER ... 5<br>AGRICULTURE AND LIVE .... 6<br>BUSINESS/ PETTY BUSINESS 7<br>STUDENT .... 8<br>OTHER PRIVATE SERVICE HOLDER 9<br>OTHER GOVERNMENT SERVICE HOLD 10<br>UNEMPLOYED 11<br>OTHER ____________ 96<br>(SPECIFY)<br>REFUSED 99	
904	Aside from your own house chores, have you done any work in the last seven days?	YES .... 1<br>NO .... 2<br>REFUSED 99	1 → 908
905	As you know, some women take up jobs for which they are paid in cash or kind. Others sell things, have a small business or work on the family farm or in the family business.<br>In the last seven days, have you done any of these things or any other work, aside from household chores?	YES .... 1<br>NO .... 2<br>REFUSED 99	1 → 908
906	Although you did not work in the last seven days, do you have any job or business from which you were absent for leave, illness, vacation, maternity leave, or any other such reasons?	YES .... 1<br>NO .... 2<br>REFUSED 99	
907	Have you done any work in the last 12 months?	YES .... 1<br>NO .... 2<br>REFUSED 99	 2 → 912<br>99 → 912
908	What is your occupation, that is, what kind of work do you mainly do?	PROFESSIONAL/TECHNICAL 1<br>DAILY WAGE LABORER .... 2<br>CIVIL SERVANT .... 3<br>PRODUCTION/TRANSPOI .... 4<br>DAILY WAGE LABOURER ... 5<br>AGRICULTURE AND LIVE .... 6<br>BUSINESS/ PETTY BUSINESS 7<br>STUDENT .... 8<br>OTHER PRIVATE SERVICE HOLDER 9<br>OTHER GOVERNMENT SERVICE HOLD 10<br>HOUSE WIFE .... 11<br>OTHER ____________ 96<br>(SPECIFY)<br>REFUSED 99	
909	In 2013, did you have a job or business, or engage in any work?	YES .... 1<br>NO 2	 2 → 911

		REFUSED 99	
910	What was your occupation in 2013, that is, what kind of work were you mainly doing?	PROFESSIONAL/TECHNICAL 1<br>DAILY WAGE LABORER .... 2<br>CIVIL SERVANT .... 3<br>PRODUCTION/TRANSPOI .... 4<br>DAILY WAGE LABOURER ... 5<br>AGRICULTURE AND LIVE .... 6<br>BUSINESS/ PETTY BUSINESS 7<br>STUDENT .... 8<br>OTHER PRIVATE SERVICE HOLDER 10<br>OTHER GOVERNMENT SERVICE HOLD 11<br>HOUSE WIFE .... 9<br>OTHER______________ 96<br>(SPECIFY)<br>REFUSED 99	
911	Who usually decides how the money **you** earn will be used: mainly you, mainly your husband, or you and your husband jointly?	RESPONDENT .... 1<br>HUSBAND .... 2<br>RESPONDENT AND<br>HUSBAND JOINTLY .... 3<br>OTHER ______________ 6<br>(SPECIFY)<br>REFUSED 99	
912	Would you say that the money that you earn is more than what your husband earns, less than what he earns, or about the same?	MORE THAN HIM .... 1<br>LESS THAN HIM .... 2<br>ABOUT THE SAME .... 3<br>HUSBAND DOESN'T<br>BRING IN ANY MONEY .... 4<br>DON'T KNOW .... 8<br>REFUSED 99	
913	Who usually decides how **your husband's** earnings will be used: you, your husband, or you and your husband jointly?	RESPONDENT .... 1<br>HUSBAND .... 2<br>RESPONDENT AND<br>HUSBAND JOINTLY .... 3<br>HUSBAND HAS<br>NO EARNINGS .... 4<br>OTHER ______________ 6<br>(SPECIFY)<br>REFUSED 99	
914	Do you feel comfortable expressing your opinions to your husband/partner on working outside the home?	YES .... 1<br>SOMEWHAT 3<br>NO .... 2<br>DON'T KNC .... 88<br>REFUSED 99	
915	Do you feel comfortable expressing your opinions to your husband/partner on going out unescorted?	YES .... 1<br>SOMEWHAT 3<br>NO .... 2<br>DON'T KNC .... 88<br>REFUSED 99	
916	Do you feel comfortable expressing your opinions to your husband/partner on the education and care of your children?	YES .... 1<br>SOMEWHAT 3<br>NO .... 2<br>DON'T KNC .... 88<br>REFUSED 99	
917	Who usually makes decisions about health care for yourself:	RESPONDENT = 1	

	you, your husband,<br>you and your husband<br>jointly, another member of your household, or someone else?	HUSBAND = 2<br>RESPONDENT & HUSBAND JOINTLY = 3<br>OTHER MALE = 4<br>OTHER FEMALE = 6<br>DON'T KNOW = 88<br>REFUSED = 99<br><br>1 2 3 4 6 88 99
918	Who usually makes decisions about making major household purchases?	1 2 3 4 6 88 99
919	Who usually makes decisions about visits to your family or relatives?	1 2 3 4 6 88 99
920	Do you own this or any other house either alone or jointly with someone else?	ALONE ONLY .... 1<br>JOINTLY ONLY .... 2<br>BOTH ALONE AND JOINTLY......... 3<br>DOES NOT OWN .... 4<br>REFUSEI. 99
921	Do you own any land either alone or jointly with someone else?	ALONE ONLY .... 1<br>JOINTLY ONLY .... 2<br>BOTH ALONE AND JOINTLY......... 3<br>DOES NOT OWN .... 4<br>REFUSED 99

922	In your opinion, is a husband justified in hitting or beating his wife in the following situations:		YES	NO	DK	REF
	If she goes out without telling him?	GOES OUT	1	2	88	99
	If she neglects the children?	NEGL. CHILDREN ...	1	2	88	99
	If she argues with him?	ARGUES	1	2	88	99
	If she uses a family planning method?	USES FP METH	1	2	88	99
	If she refuses to have sex with him?	REFUSES SEX	1	2	88	99
	If she burns the food?	BURNS FOOD.......	1	2	88	99
	If she refuses to do housework?	REFUSES HOUSEWOR	1	2	88	99

924	Have you heard of these illnesses?	YES	NO	M	DK	R
	(1)HIV/AIDS	1	2	3	8	9
	(2)Hepatitis B	1	2	3	8	9
		1	2	3	8	9
	(4)Dysentery	1	2	3	8	9
	(5)Gonorrhea	1	2	3	8	9
	(6)Genital herpes	1	2	3	8	9
	(7)Genital warts	1	2	3	8	9

10. OTHER HEALTH ISSUES

NO.	QUESTIONS AND FILTERS	CODING CATEGORIES	SKIP
1001	Many different factors can prevent women from getting family planning advice or treatment for themselves. When you want to get family planning advice or treatment, is each of the following a big problem or not?<br><br>Getting permission to go to the doctor?<br>Getting money needed for advice or treatment?<br>The distance to the health facility?<br>Not wanting to go alone?<br>Not having time because of work?<br>No one to look after children?	BIG PROB-LEM / NOT A BIG PROB-LEM / DK / R<br>PERMISSION TO GO ... 1 2 88 99<br>GETTING MONEY ..... 1 2 88 99<br>DISTANCE .... 1 2 88 99<br>GO ALONE .... 1 2 88 99<br>NO TIME DUE TO WORK . 1 2 88 99<br>NO CHILDCARE 1 2 88 99	
1002	Are you covered by any health insurance?	YES .... 1<br>NO .... 2<br>DON'T KNOW .... 88<br>REFUSED 99	NO, DON'T KNOW, REFUSED → 1005
1003	What type of health insurance are you covered by?<br>RECORD ALL MENTIONED.	NATIONAL/DISTRICT HEALTH INSURANCE (NHIS) .... 1<br>HEALTH INSURANCE THROUGH EMPLOYER .... 2<br>MUTUAL HEALTH ORGANIZATION/ COMMUNITY-BASED HEALTH INSURANCE .... 3<br>OTHER PRIVATELY PURCHASED COMMERCIAL HEALTH INSURANCE 4<br>OTHER ________ 96<br>(SPECIFY)<br>DON'T KNOW 88<br>REFUSED 99	
PK_10_1	Can I see your insurance card or other evidence of this insurance coverage?	Yes, Card seen with current cover 1<br>Yes, Card seen but no current cover 2<br>No, Card not seen 3<br>REFUSED 99	
1004	Does your insurance cover any of the following maternity benefits:<br><br>a) Antenatal health care?<br>b) Childbirth health care in a health facility?<br>c) Postnatal health care for the mother?<br>d) Postnatal health care for the child?<br>e) Cash benefits during maternity leave?<br>f) Family planning methods or services?<br>g) Abortion services?<br>h) Other?	YES / NO / DK / RF<br>ANTENATAL (ANC) 1 2 8 9<br>CHILDBIRTH 1 2 8 9<br>PNC MOTHER 1 2 8 9<br>PNC CHILD ... 1 2 8 9<br>CASH BENEFITS 1 2 8 9<br>FAMILY PLANNING 1 2 8 9<br>ABORTION 1 2 8 9<br>OTHER 1 2 8 9	
1005	At the start of 2013, were you covered by any health insurance?	YES .... 1<br>NO .... 2<br>DON'T KNOW .... 88<br>REFUSED 99	NO, DON'T KNOW, REFUSED → 1008
1006	At the start of 2013, what type of health insurance are you<br>RECORD ALL MENTIONED.	NATIONAL/DISTRICT HEALTH INSURANCE (NHIS) .... 1<br>HEALTH INSURANCE THROUGH EMPLOYER .... 2<br>MUTUAL HEALTH ORGANIZATION/ COMMUNITY-BASED HEALTH	

		INSURANCE .... 3<br>OTHER PRIVATELY PURCHASED<br>COMMERCIAL HEALTH INSURANCE 4<br>OTHER ______________________ 96<br>(SPECIFY)<br>DON'T KNOW 88<br>REFUSED 99	
1007	At the start of 2013, did your insurance cover any of the benefits:<br><br>a) Antenatal health care?<br>b) Childbirth health care in a health facility?<br>c) Postnatal health care for the mother?<br>d) Postnatal health care for the child?<br>e) Cash benefits during maternity leave?<br>f) Family planning methods or services?<br>g) Abortion services?<br>h) Other?	YES NO DK RF<br>ANTENATAL (ANC) 1 2 8 9<br>CHILDBIRTH 1 2 8 9<br>PNC MOTHER 1 2 8 9<br>PNC CHILD ... 1 2 8 9<br>CASH BENEFITS 1 2 8 9<br>FAMILY PLANNING 1 2 8 9<br>ABORTION 1 2 8 9<br>OTHER 1 2 8 9	
1008	RECORD THE TIME.	HOUR ....<br>MINUTES	

FOLLOW-UP INFORMATION

FOLLOW-UP INFORMATION

Thank you for this information. We are planning to conduct follow-up interviews with some women whom we are interviewing now. If you are selected for a follow-up interview, you will be contacted beforehand using the contact information that you have given us, and you will be provided with information on how to participate in the interview.

CONTACT 1

FULL NAME: ______________________________

RELATIONSHIP TO YOU: ______________________________

FULL ADDRESS: ______________________________

____

____

PHONE NUMBER: ______________________________

____

CONTACT 2

FULL NAME: ______________________________

RELATIONSHIP TO YOU: ______________________________

FULL ADDRESS: ______________________________

____

____

PHONE NUMBER: ______________________________

OTHER HOUSEHOLD CONTACT:

What is the name and phone number of someone else within your household?

NAME: ______________________________

PHONE: ______________________________

RELATIONSHIP TO YOU: ______________________________

PLANS TO MOVE:

Does your family/household have any plans to move in the next two years? YES NO

IF YES: Where do you plan to move to? ADDRESS: ______________________________

____

____

Consent to Willows data linkage

We would like to access information collected by Willows Field Educators during their home visits from 2014-2016. Willows Foundation Field Educators may have visited you over the past few years about referrals related to pregnancy, contraceptive use and other women's health issues. Since most people cannot recall details about each home visit very easily, we are asking for your permission to obtain the following information from the Willows Foundation:

- Information about the number and types of visits made to you by the Willows Field Educators.
- Data about your background information, pregnancy history and contraceptive use history.
- If you were not visited by the Willows Foundation no additional information about you will be obtained, but we would still like to get your permission to check their records.
- This information will be kept private and will not be available to anyone outside of our research team.

YES NO

INSTRUCTIONS:
ONLY ONE CODE SHOULD APPEAR IN ANY BOX.
COLUMN 1 REQUIRES A CODE IN EVERY MONTH.

INFORMATION TO BE CODED FOR EACH COLUMN

COLUMN 1: BIRTHS, PREGNANCIES, CONTRACEPTIVE USE**

B BIRTHS
P PREGNANCIES
T TERMINATIONS

0 NO METHOD
1 FEMALE STERILIZATION
2 MALE STERILIZATION
3 IUD
4 INJECTABLES
5 IMPLANTS
6 PILL
7 CONDOM

9 EMERGENCY CONTRACEPTION
J RHYTHM METHOD/STANDARD DAYS METHOD/CYCLEBE
K LACTATIONAL AMENORRHEA METHOD
M WITHDRAWAL
X OTHER MODERN METHOD
Y OTHER TRADITIONAL METHOD

COLUMN 2: DISCONTINUATION OF CONTRACEPTIVE USE

0 INFREQUENT SEX/HUSBAND AWAY
1 BECAME PREGNANT WHILE USING
2 WANTED TO BECOME PREGNANT
3 HUSBAND/PARTNER DISAPPROVED
4 WANTED MORE EFFECTIVE METHOD
5 SIDE EFFECTS/HEALTH CONCERNS
6 LACK OF ACCESS/TOO FAR
7 COSTS TOO MUCH
8 INCONVENIENT TO USE
F UP TO GOD/FATALISTIC
A DIFFICULT TO GET PREGNANT/MENOPAUSAL
D MARITAL DISSOLUTION/SEPARATION
X OTHER ______________________________
(SPECIFY)
Z DON'T KNOW

* Year of fieldwork is assumed to be 2018. For fieldwork beginning in 2019 or 2020, the years should be adjusted.

** Response categories may be added for other methods, including fertility awareness methods.

			1	2	
	12 DEC	1			
	11 NOV	2			
	10 OCT	3			
	9 SEP	4			
2	8 AUG	5			2
0	7 JUL	6			0
1	6 JUN	7			1
8	5 MAY	8			8
*	4 APR	9			*
	3 MAR	10			
	2 FEB	11			
	1 JAN	12			
	12 DEC	13			
	11 NOV	14			
	10 OCT	15			
	9 SEP	16			
2	8 AUG	17			2
0	7 JUL	18			0
1	6 JUN	19			1
7	5 MAY	20			7
*	4 APR	21			*
	3 MAR	22			
	2 FEB	23			
	1 JAN	24			
	12 DEC	25			
	11 NOV	26			
	10 OCT	27			
	9 SEP	28			
2	8 AUG	29			2
0	7 JUL	30			0
1	6 JUN	31			1
6	5 MAY	32			6
*	4 APR	33			*
	3 MAR	34			
	2 FEB	35			
	1 JAN	36			
	12 DEC	37			
	11 NOV	38			
	10 OCT	39			
	9 SEP	40			
2	8 AUG	41			2
0	7 JUL	42			0
1	6 JUN	43			1
5	5 MAY	44			5
*	4 APR	45			*
	3 MAR	46			
	2 FEB	47			
	1 JAN	48			
	12 DEC	49			
	11 NOV	50			
	10 OCT	51			
	9 SEP	52			
2	8 AUG	53			2
0	7 JUL	54			0
1	6 JUN	55			1
4	5 MAY	56			4
*	4 APR	57			*
	3 MAR	58			
	2 FEB	59			
	1 JAN	60			
	12 DEC	61			
	11 NOV	62			
	10 OCT	63			
	9 SEP	64			
2	8 AUG	65			2
0	7 JUL	66			0
1	6 JUN	67			1
3	5 MAY	68			3
*	4 APR	69			*
	3 MAR	70			
	2 FEB	71			
	1 JAN	72			

END OF SURVEY:

You have now reached the end of the interview. Thank you for your time and responses. This has been extremely helpful. As I said in the beginning, the purpose of this discussion was to help us learn about women's health in Ghana I also want to remind you that all of your responses will remain confidential.

Do you have any further questions for me at this time?

Thank you again for your help.

SIGNATURE OF INTERVIEWER DATE: ______________________

END. GO TO INTERVIEWER OBSERVATIONS.

INTERVIEWER'S OBSERVATIONS

TO BE FILLED IN AFTER COMPLETING INTERVIEW

COMMENTS ABOUT INTERVIEW:

COMMENTS ON SPECIFIC QUESTIONS:

ANY OTHER COMMENTS:

EDITOR'S OBSERVATIONS
